# Targeted long-read sequencing enables comprehensive analysis of the genetic and epigenetic landscape of inherited myopathies

**DOI:** 10.1038/s41467-026-75144-z

**Published:** 2026-07-04

**Authors:** Dennis Yeow, Andre L. M. Reis, Igor Stevanovski, Neysa Njo, Laura I. Rudaks, Bianca R. Grosz, Joanne S. Sy, Leah Kemp, Sanjog R. Chintalaphani, Michael Chin, Marion Stoll, Danqing Zhu, Christina Liang, Katrina A. Morris, Andrew Hannaford, Ehsan Shandiz, Kate E. Ahmad, Shadi El-Wahsh, Stephen W. Reddel, Robert Boland-Freitas, Roula Ghaoui, Stephanie Barnes, Jonathan Sturm, Anna Willard, Mahi Jasinarachchi, Simon Hawke, Neil G. Simon, Lisa Worgan, David Manser, Michel Tchan, Neil C. Griffith, Ryan L. Davis, Michael C. Fahey, Carolyn M. Sue, Pamela A. McCombe, Karl Ng, Marina L. Kennerson, Pak Leng Cheong, Kishore R. Kumar, Ira W. Deveson

**Affiliations:** 1 Neurology Department, Concord Repatriation General Hospital, Sydney, NSW Australia; 2 Faculty of Medicine and Health, University of Sydney, Sydney, NSW Australia; 3 Translational Neurogenomics Group, Garvan Institute of Medical Research, Sydney, NSW Australia; 4 Neurodegenerative Service, Princes of Wales Hospital & Neuroscience Research Australia, Sydney, NSW Australia; 5 Genomic Technologies Lab, Garvan Institute of Medical Research, Sydney, NSW Australia; 6 Faculty of Medicine and Health, University of New South Wales, Sydney, NSW Australia; 7 Clinical Genetics Unit, Royal North Shore Hospital, Sydney, NSW Australia; 8 Northcott Neuroscience Laboratory, ANZAC Research Institute, Sydney Local Health District, Sydney, NSW Australia; 9 Department of Neuropathology, Royal Prince Alfred Hospital, Sydney, NSW Australia; 10 Molecular Medicine Laboratory, Concord Repatriation General Hospital, New South Wales Health Pathology, Sydney, NSW Australia; 11 Department of Neurology, Royal North Shore Hospital, Sydney, NSW Australia; 12 Department of Neurology, Toowoomba Base Hospital, Toowoomba, QLD Australia; 13 Centre for Clinical Research, University of Queensland, Brisbane, QLD Australia; 14 Department of Neurology, Blacktown Hospital, Sydney, NSW Australia; 15 Department of Neurology, Central Adelaide Local Health Network, Royal Adelaide Hospital, Adelaide, SA Australia; 16 Department of Neurology, Hornsby Ku-ring-gai Hospital, Sydney, NSW Australia; 17 Central Coast Neurosciences Research, Tumbi Umbi, NSW Australia; 18 The University of Newcastle, Newcastle, NSW Australia; 19 Department of Neuroscience, School of Translational Medicine, Alfred Health and Monash University, Melbourne, VIC Australia; 20 Department of Neurology and Neurological Research, St. Vincent’s Hospital Melbourne, Melbourne, VIC Australia; 21 Central West Neurology and Neurosurgery, Orange, NSW Australia; 22 Faculty of Medicine and Health Sciences, Macquarie University, Sydney, NSW Australia; 23 Clinical Genetics Service, Royal Prince Alfred Hospital, Sydney, NSW Australia; 24 Department of Genetic Medicine, Westmead Hospital, Sydney, NSW Australia; 25 Department of Neurology, Liverpool Hospital, Sydney, NSW Australia; 26 Neurogenetics Research Group, Kolling Institute, School of Medical Sciences, Faculty of Medicine and Health, University of Sydney and Northern Sydney Local Health District, Sydney, NSW Australia; 27 Department of Paediatrics, Monash University, Melbourne, VIC Australia; 28 Neurology Department, Royal Brisbane & Women’s Hospital, Brisbane, QLD Australia; 29 Institute of Precision Medicine and Bioinformatics, Sydney Local Health District, Sydney, NSW Australia; 30 ANZAC Research Institute, Sydney Local Health District, Sydney, NSW Australia

**Keywords:** Medical genetics, Neuromuscular disease, DNA sequencing

## Abstract

The genetic variants that cause inherited myopathies vary widely in type, size and sequence context, encompassing small sequence variants, large structural variants, repeat expansions, and more complex events, such as the D4Z4 macrosatellite contraction and hypomethylation that causes facioscapulohumeral muscular dystrophy. Many of these are challenging to characterise using next-generation sequencing and other older molecular technologies. To address this, we developed a targeted long-read sequencing assay and bioinformatics analysis framework that captures the full suite of genes, variants and epigenetic signatures currently implicated in inherited myopathies. Applying this to a cohort of myopathy patients, we demonstrate the analytical validity of our approach and its improved accuracy and resolution compared to existing methods. Our assay led to new genetic diagnoses in 35.5% (11/31) of patients who remained undiagnosed after standard clinical genetic testing. This methodology constitutes a single streamlined assay for comprehensive genetic and epigenetic characterisation of inherited myopathies.

## Abstract (web summary)

Inherited myopathies involve diverse genetic variants that are difficult to detect with standard methods. Here, the authors develop a targeted long‑read sequencing assay that improves variant detection and enables new diagnoses, providing a unified approach for genetic and epigenetic characterisation.

## Introduction

Genetic myopathies (inherited muscle diseases) are a large group of heterogeneous disorders that affect an estimated 1 in 1700–4500 people^1,2^. Molecular characterisation of patients with genetic myopathies is essential to understanding the genetic basis of disease, developing new therapies, and providing patients with an accurate diagnosis, which facilitates disease-specific treatment, prognosis, genetic counselling, family planning, and access to clinical trials^3^. However, the diagnostic process is complicated by several interrelated challenges. Genetic myopathies exhibit high phenotypic heterogeneity, i.e., patients with the same genetic cause may present with different clinical phenotypes, disease severity and age of onset. There is also significant phenotypic overlap between different types of genetic myopathy, i.e., patients with different genetic causes may present with similar clinical features. Finally, the known genetic causes of myopathy are numerous and highly diverse, occurring in a large number of genes (>300 described to date) across the nuclear and mitochondrial genomes. These include single and multi-nucleotide variants (SNVs, MNVs), small insertions and deletions (indels), structural and copy number variants (SVs, CNVs), expanded short (STRs) and variable number tandem repeats (VNTRs), and contractions of the D4Z4 macrosatellite that may cause facioscapulohumeral muscular dystrophy (FSHD)^4^. Epigenetic regulation of gene promoters, STRs or the D4Z4 macrosatellite is also central to the disease mechanism in certain myopathies – methylation profiling can be informative for diagnosis and prognosis in these disorders^4,5^.

In the absence of a single assay that can capture all potential molecular causes, these genetic and clinical complexities limit the ability to diagnose genetic myopathies and understand their pathophysiology^4^. While short read next-generation sequencing (NGS) provides high-throughput assessment of hundreds of myopathy genes, it has limited capacity to resolve repetitive or non-unique genetic loci, phase distantly located genetic variants, identify SVs/CNVs and other complex structural rearrangements, characterise repeat expansions/contractions, and detect epigenetic changes. To date, application of NGS to undiagnosed myopathy cohorts has resulted in diagnostic yields of 21–65%^6–9^. Alternative, lower-throughput, disorder-specific testing methodologies are required to diagnose myopathies refractory to NGS^4,10^. For example, the common genetic myopathies myotonic dystrophy type 1 (DM1) and 2 (DM2) are caused by STR expansions in the *DMPK* and *CNBP* genes, and typically require loci-specific repeat-primed PCR (RP-PCR) or Southern blot (SB) for diagnosis^11^. However, the rapidly increasing number of newly described genetic myopathies caused by STR expansions (e.g., oculopharyngodistal myopathies [OPDM]) lack readily accessible testing, as each newly described loci requires design and validation of a gene-specific RP-PCR or SB assay^4^.

FSHD, the third most common genetic myopathy, is especially complex. Genetic diagnosis of FSHD may involve a combination of SB to identify chromosome 4 D4Z4 macrosatellite repeat contractions and detect the FSHD-permissive 4qA haplotype (cf. non-permissive 4qB, 10qA, and 10qB haplotypes) that contribute to FSHD type 1 (FSHD1), NGS to identify SNVs or indels in genes that cause FSHD type 2 (FSHD2), and/or bisulphite sequencing (BSS) to detect D4Z4 hypomethylation, seen in both FSHD1 and FSHD2^12^. Although SB was the gold standard for FSHD1 diagnosis, it is low throughput, labour-intensive, imprecise and may generate false-positive and false-negative results as a result of uncommon, structurally complex D4Z4 alleles^12^. Optical genome mapping and molecular combing are newer techniques that are superior to SB for detection of D4Z4 contractions and SVs, but neither identifies the sequence variants that cause FSHD2 or the abnormal D4Z4 methylation profile seen in FSHD^4,13–15^. As a result, there is a great need for an integrated approach that fully resolves the genetic and epigenetic architecture of FSHD (and genetic myopathies in general) in order to better understand the molecular mechanisms of the disorder and provide patients with accurate, timely diagnoses.

Given these challenges, we reasoned that long-read sequencing (LRS), a newer class of sequencing technology that addresses many of the limitations of NGS, would be particularly well suited to the molecular characterisation of genetic myopathies^4^. LRS enables detection of large, repetitive and/or complex genetic variants and mapping of reads across homologous or repetitive regions of the genome. It also readily phases distant variants to identify compound heterozygous events in the absence of parental sequencing data. Both of the currently available LRS platforms, Oxford Nanopore Technologies (ONT) and Pacific Biosciences (PacBio), are also able to concurrently assess epigenetic changes without the need for additional sample processing^16^. Therefore, by capturing all known genetic and epigenetic alterations in a single test, LRS may better navigate genotype/phenotype heterogeneity and improve genetic myopathy diagnosis.

We previously demonstrated the use of targeted LRS to simultaneously characterise all STR expansions implicated in neuromuscular disease^17^. Here, we build on this approach by developing a targeted LRS assay and integrated bioinformatics framework that represents a single comprehensive assay suitable for diagnostic investigation of all known forms of genetic myopathy. This approach delivers new genetic insights and diagnoses within a cohort of patients with suspected genetic myopathies who have previously received inconclusive or negative results via conventional clinical genetic testing.

## Results

### A targeted ONT LRS assay for genetic myopathies

We developed a targeted ONT LRS assay using adaptive sampling to enrich for a ~60 Mbase target panel encompassing all loci implicated in genetic myopathies (*n* = 331 at the time of panel creation in March 2023). The panel included individual myopathy genes, myopathy-causing STR loci, the mitochondrial genome, and the subtelomeric regions of 4q and 10q relevant to FSHD (Supplementary Data 1). We developed an efficient bioinformatics pipeline to detect, phase and annotate genetic variants of all types, and determine 5-methylcytosine (5mC) DNA methylation frequencies within genomic target regions (see Methods). We also developed a new bioinformatics software package, d4z4ling (read: “dazzling”), that uses LRS data to resolve the genetic and epigenetic contributors to FSHD, described later.

**Fig. 1:**
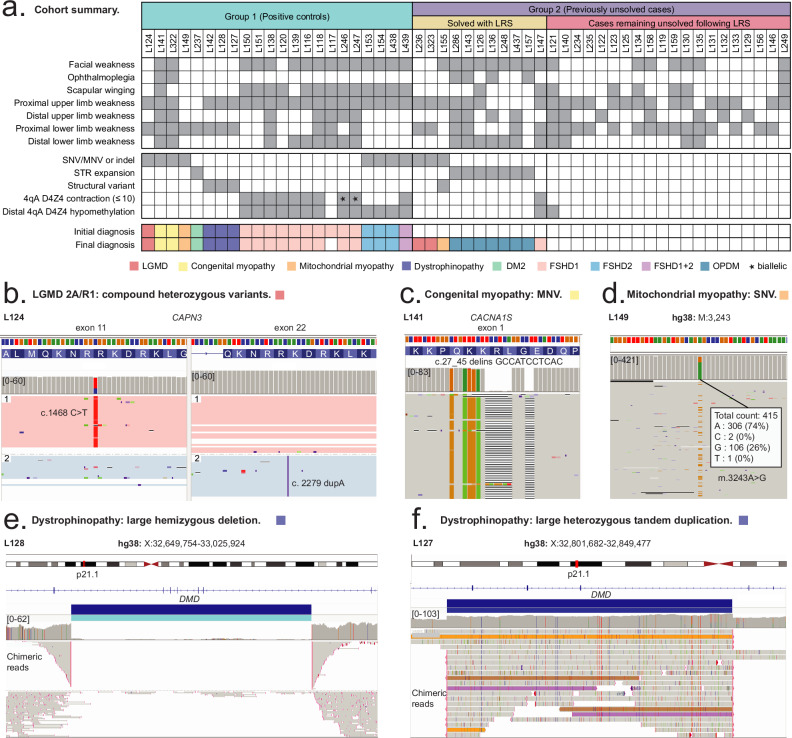
Myopathy cohort characteristics and examples of known genetic variants identified by Oxford Nanopore Technologies long-read sequencing (ONT LRS). **a** Myopathy cohort summary with participants grouped into positive controls, newly solved cases following ONT LRS, and cases remaining unsolved following ONT LRS. The upper matrix summarises the distribution of muscle weakness. The middle matrix summarises the molecular findings contributing to each diagnosis. The lower matrix shows genetic diagnoses before and after ONT LRS. **b** Case L124 (known diagnosis of limb-girdle muscular dystrophy [LGMD] 2A/R1) harbours a missense variant (c.1468C>T) in exon 11 and duplication (c.2279dupA) in exon 22 of *CAPN3* in *trans* (red & blue reads representing different alleles). **c** Case L141 (known diagnosis of congenital myopathy 18) harbours a complex, homozygous, multinucleotide variant (c.27_45delinsGCCATCCTCAC) in exon 1 of *CACNA1S*. **d** Case L149 (known diagnosis of a mitochondrial myopathy) harbours m.3243A>G at a heteroplasmy of 26% in blood. **e** Case L128 (known diagnosis of Duchenne muscular dystrophy) harbours a large deletion of *DMD* exons 3–7. ONT LRS generated chimeric reads spanning the deletion, allowing for identification of the deletion breakpoint and the presence of a small 9 bp insertion at the deletion site: c.93+60377_650-16627delinsACTTGTTTG. **f** Case L127 (manifesting *DMD* carrier) harbours a *DMD* exon 5–7 duplication deletion that was located in tandem (indicated by individual chimeric long reads [examples highlighted in purple and orange] that map across both sides of the duplicated interval) and flanked by a CT dinucleotide sequence: c.649+3234_649+3235ins[CT;264+2035_649+3234;CT].

We applied our LRS assay to 53 participants (Fig. 1a), including those with an established genetic myopathy diagnosis (*n* = 22; ‘Group 1’) (Tables 1, 2) and those with suspected genetic myopathies who remained genetically unsolved or incompletely solved after previous clinical genetic testing (*n* = 31; ‘Group 2’; Supplementary Fig. 1). Participants in Group 2 had a mean disease duration (symptom onset to study recruitment) of 15.5 years. The cohort was processed at a rate of 1–3 participant samples per ONT flow cell, obtaining 18–250-fold average coverage and 16–48 kb read length N_50_ within genomic target regions (Supplementary Fig. 2a–d). An average of 210–1,824-fold coverage was obtained for the mitochondrial genome (Supplementary Fig. 2e).

**Table 1:** ONT LRS results in myopathy patients with prior known genetic diagnoses (excluding FSHD)

Case ID	Diagnosis	Prior Genetic Test	Prior Genetic Results	ONT LRS Results
L124	LGMD A/R1	NGS TGP	NM_000070.3(*CAPN3*):c.1468C>T(;)2279dupA	NM_000070.3(*CAPN3*):c.[1468C>T];[2279dupA]
L141	Congenital myopathy 18	NGS exome-based neuromuscular panel	NM_000069.3(*CACNA1S*):c.[27_45delinsGCCATCCTCAC]; [27_45delinsGCCATCCTCAC]	NM_000069.3(*CACNA1S*):c.[27_45delinsGCCATCCTCAC]; [27_45delinsGCCATCCTCAC]
L322	Congenital myopathy 1B	NGS TGP	NM_000540.3(*RYR1*):c.10348-6C>G(;)12829G>T(;)14524G>A	NM_000540.3(*RYR1*)*:*c.[10348-6C>G;14524G>A];[12829G>T]
L149	Mitochondrial myopathy	m.32432 A > G PCR-RFLP analysis	NC_012920.1(*MT-TL1*):m.3243A>G (42% heteroplasmy in blood)	NC_012920.1(*MT-TL1*):m.3243A>G (26% heteroplasmy in blood)
L237	DM2	*CNBP* repeat-primed PCR	*CNBP* intron 1 CCTG expansion detected (size not determined)	*CNBP* intron 1 (CCTG)_2275–2870_ expansion
L142	DMD	*DMD* MLPA	*DMD* exon 45 deletion detected	NM_004006.3(*DMD*):c.[6439-94713_6614+8075del];[0]
L128	DMD	*DMD* MLPA	*DMD* exons 3–7 deletion detected	NM_004006.3(*DMD*):c.[93+60377_650-16627delinsACTTGTTTG];[0]
L127	Manifesting *DMD* carrier	*DMD* MLPA	*DMD* exons 5–7 duplication detected (assumed to be in tandem)	NM_004006.3(*DMD*):c.649+3234_649+3235ins[CT;264+2035_649+3234;CT];[=]

*DM2* myotonic dystrophy type 2, *DMD* Duchenne muscular dystrophy, *FSHD* facioscapulohumeral muscular dystrophy, *LGMD* limb-girdle muscular dystrophy, *MLPA* multiplex ligation-dependent probe amplification, *NGS* next-generation sequencing, *ONT LRS* Oxford Nanopore Technologies long-read sequencing, *PCR* polymerase chain reaction, *RFLP* restriction fragment length polymorphism, *TGP* targeted gene panel.

Our LRS assay identified previously known causative variants in 21 of 22 participants in Group 1, including SNVs, MNVs, indels, mtDNA variants, STR expansions, SV/CNVs, and D4Z4 macrosatellite contractions, 4qA/B haplotypes and DNA hypomethylation (Fig. 1a, Supplementary Fig. 3; Tables 1, 2). The exception was case L117, for whom a previous diagnosis of FSHD1 was revoked based on findings from LRS data (see below). LRS also provided more complete genetic characterisation than available from previous testing in several cases. For example, the capacity to phase distantly located variants allowed for confirmation that pathogenic variants in *CAPN3* and *RYR1* were biallelic in two cases (L124, L322), in the absence of sequencing data from family members (Fig. 1b; Supplementary Fig. 3a). In case L237, with a diagnosis of DM2, previous RP-PCR identified the presence of a pathogenic CCTG STR expansion in intron 1 of *CNBP* but was unable to size it accurately. LRS reads spanning the STR site confirmed an upper size of 2870 repeat copies, with some evidence of repeat length mosaicism (ranging from 2275–2870 repeats) (Supplementary Fig. 3b). For three dystrophinopathy cases (L142, L128, L127) previously diagnosed using *DMD* multiplex ligation-dependent probe amplification (MLPA), LRS identified these CNVs and resolved precise CNV breakpoints and orientations (Fig. 1e, f; Supplementary Fig. 3c). For example, in case L127, MLPA previously identified the presence of a duplication of *DMD* exons 5–7 but could not determine if the duplicated segment was in tandem, nor its orientation. LRS reads proved the duplication was in tandem and non-inverted (Fig. 1f). This is clinically relevant since extragenic (non-tandem) *DMD* duplications are not expected to cause a dystrophinopathy and are largely benign^18^. Knowledge of exact breakpoints for large SVs/CNVs within *DMD* can also elucidate the mechanisms by which they arise (Supplementary Fig. 4)^19^. For example, in case L128, both ends of the duplicated segment were found to be located within partially homologous LINE-1 retrotransposon elements within introns 4 and 7, indicating it may have arisen from LINE-LINE-mediated nonallelic homologous recombination^20^.

In Group 2, our LRS assay led to a new genetic diagnosis in 35.5% (11/31) (Table 3), and revealed an array of new genetic and epigenetic findings, explored below. LRS also provided important phasing information for previously identified suspicious variants of uncertain significance (VUS) in several of the cases that remained genetically unsolved (Supplementary Note 1). Overall, these results demonstrated the validity of our experimental and analytical approaches, and the capacity of LRS to improve genetic characterisation of patients with myopathies.

### A new method for FSHD analysis

**Fig. 2:**
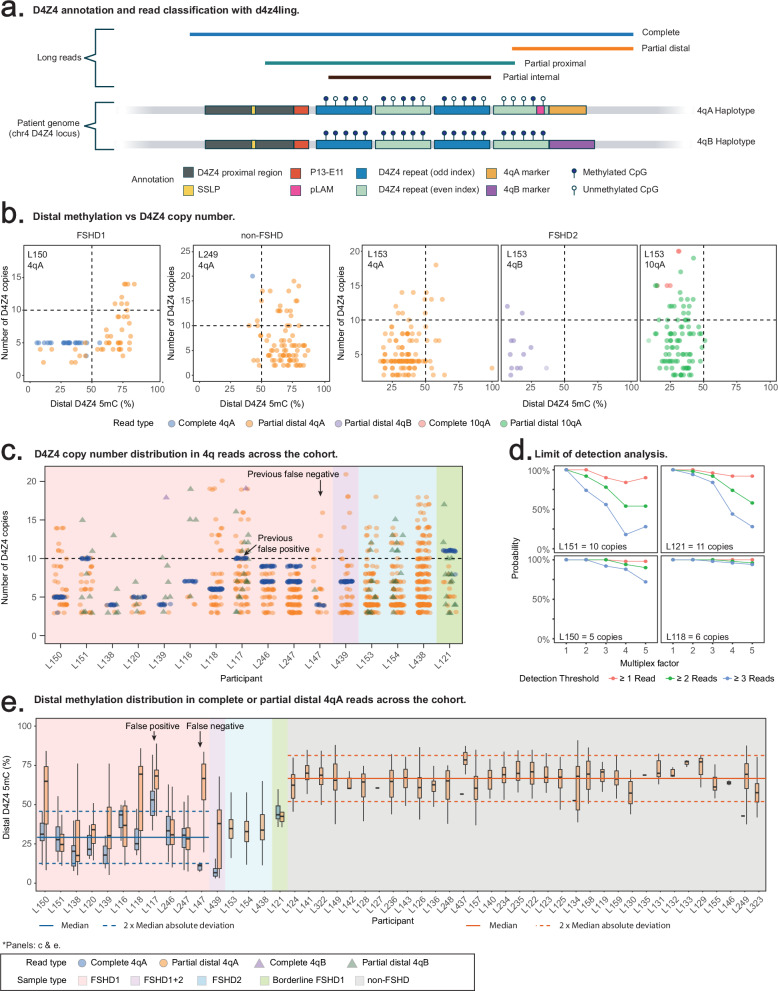
Targeted Oxford Nanopore Technologies long-read sequencing characterises the genetic and epigenetic architecture of facioscapulohumeral muscular dystrophy (FSHD). **a** Chromosome 4 D4Z4 read classification and annotation schematic. Reads are classified as complete, partial distal, partial proximal, or partial internal based on alignment. Reads are annotated with specific sequence features: simple sequence length polymorphism [SSLP], p13-E11, D4Z4 unit, pLAM, and 4qA/4qB markers. 5mC methylation is measured at all CpG sites and per-read distal-most D4Z4 unit average methylation fraction is calculated. **b** Per-read D4Z4 copy number and distal-most D4Z4 unit methylation fraction plots. Dashed lines represent an arbitrary 50% methylation value (vertical) and the 10-copy diagnostic threshold for FSHD1 (horizontal). Representative cases are shown for FSHD1 (L150: one distally-hypomethylated, 5-copy 4qA D4Z4 array allele and one normomethylated, non-contracted 4qA D4Z4 allele), non-FSHD (L249: normomethylated, non-contracted 4qA D4Z4 arrays), and FSHD2 (L153: distally-hypomethylated, non-contracted D4Z4 arrays on 4qA, 4qB and 10qB alleles). **c** Per-read 4q D4Z4 copy number distribution of FSHD patients and special cases (L117, incorrectly diagnosed with FSHD1 by Southern blot [‘false positive’]; L147, where FSHD1 was incorrectly excluded by Southern blot [‘false negative’]; and L121, harbouring a borderline contracted 11-copy D4Z4 array). The dashed horizontal line represents the 10-copy diagnostic threshold for FSHD1. **d** D4Z4 copy number limit of detection analysis. Probability of detecting reads completely spanning the D4Z4 array is plotted against simulated sample multiplex factor in representative individuals with 5-, 6-, 10-, and 11-copy D4Z4 arrays. Curves correspond to detection thresholds requiring ≥ 1, ≥ 2, or ≥ 3 reads. **e** Boxplots of per-read distal-most 4qA D4Z4 unit methylation fraction across the cohort, grouped by diagnosis: FSHD1, FSHD1 + 2, FSHD2, borderline 11-copy D4Z4 array, and non-FSHD cases. Solid and dashed horizontal lines indicate median and ±2 × median absolute deviation methylation fraction, respectively, for complete 4qA reads from FSHD1 cases (blue) and distal 4qA reads from non-FSHD cases (orange). Boxplots show the median (centre line), first and third quartiles (hinges), and the most extreme values within 1.5 × the interquartile range of the hinges (whiskers). The number of reads contributing to each boxplot varied between samples (1 to 294). Source data for (**b**–**e**) are provided in a Source Data file.

An integrated molecular and computational method for analysis of patients with FSHD forms a key component of our assay. Our targeted LRS panel captures the subtelomeric regions of chromosomes 4q, containing the D4Z4 macrosatellite that is contracted in FSHD1, and 10q, containing a highly homologous D4Z4 macrosatellite that does not contribute to FSHD. We developed a DNA shearing and size selection method to maximise yields of ONT reads long enough to span a contracted, pathogenic D4Z4 haplotype (10 copies; >33 kb; see Methods). Our new software package, d4z4ling, analyses reads that fully or partially span the D4Z4 repeat array to determine D4Z4 copy number, DNA methylation status and haplotype (4qA, 4qB, 10qA, 10qB), and identifies other supporting sequence features on each D4Z4 unit (Fig. 2a, b). The genes involved in FSHD2 (*SMCHD1*, *DNMT3B*, *LRIF1*) are simultaneously captured and sequenced for functionally relevant variants. Our LRS assay therefore captures all known genomic and epigenomic features relevant to FSHD.

We first evaluated our method via analysis of 10 cases previously diagnosed with FSHD1 by SB, 3 cases of FSHD2 (from a single family) and 1 case of FSHD1 + 2. We detected a pathogenic D4Z4 repeat contraction (≤10 repeats) in *cis* with a permissive 4qA haplotype in 9/10 FSHD1 cases (Table 2; Fig. 2c). The remaining case (L117) was shown not to harbour a contracted D4Z4 array on a 4qA allele (see below). The D4Z4 repeat number identified by d4z4ling matched the D4Z4 repeat number estimated by SB exactly in 4/10 cases and was within 2 repeats of all SB estimates (Table 2). The margin of error in the D4Z4 repeat number count estimated by SB increases progressively with increasing D4Z4 repeat size (reported by our local diagnostic laboratory as ± 2 kb for a 16.3 kb fragment and ± 4.2 kb for a 45.3 kb fragment). In contrast, d4z4ling provides a precise readout of the D4Z4 repeat number supported by multiple independent ‘complete’ sequencing reads. The observed discrepancies are therefore consistent with imprecision in SB size estimates. To assess minimum sequencing requirements to confidently detect D4Z4 contractions, we conducted a downsampling experiment using data from four cases with 5-, 6-, 10- and 11-copy D4Z4 alleles (see Methods). For all cases, complete 4qA D4Z4 reads were detected with >90% confidence at a multiplexing rate of 3 samples per flow cell. Reducing this to 2 samples per flow cell was necessary to ensure 100% confidence for the 10- and 11-copy arrays (Fig. 2d, Supplementary Fig. 5). While performance may vary based on the specifics of an individual case, these results indicate that reliable FSHD profiling can be achieved when running 2–3 samples per flow cell, thus establishing a streamlined and comprehensive strategy with sufficient data output for confident detection of D4Z4 repeat contractions.

**Table 2:** Genetic & epigenetic variants from ONT LRS in a cohort of patients with prior diagnoses of FSHD

Case ID	Diagnosis before ONT LRS	Prior genetic results		ONT LRS results				Diagnosis following ONT LRS
		D4Z4 copy number (Haplotype)^*^	Other genetic results	D4Z4 copy number (Haplotype)	Methylation pattern	Mean (S.D.) Contracted Allele SSLP Length, bp	Other genetic result	
L150	FSHD1	5	-	5 (4qA)	FSHD1	160.83 (1.77)	-	FSHD1
L151	FSHD1	9	-	10 (4qA)	FSHD1	161.4 (1.53)	-	FSHD1
L138	FSHD1	4 (4qA)	-	4 (4qA)	FSHD1	160.6 (2.27)	-	FSHD1
L120	FSHD1	6	-	5 (4qA)	FSHD1	163.5 (2.52)	-	FSHD1
L139	FSHD1	4 (4qA)	-	4 (4qA)	FSHD1	161 (2.00)	-	FSHD1
L116	FSHD1	6	-	7 (4qA)	FSHD1	160.5 (2.17)	-	FSHD1
L117	FSHD1	10 (4qA)	-	10 (10qA)	Normal	174.91 (1.14)	-	Not FSHD
L118	FSHD1	5	-	6 (4qA)	FSHD1	160.86 (2.12)	-	FSHD1
L246^†^	FSHD1	7 & 9	FSHD1 methylation pattern on BSS	7 (4qA),9 (4qA)	FSHD1 (biallelic)	161.24 (2.54), 160.8 (3.49)		FSHD1
L247^†^	FSHD1	6 & 8	FSHD2 methylation pattern on BSS	7 (4qA), 9 (4qA)	FSHD1 (biallelic)	160.22 (2.32), 160.17 (2.62)		FSHD1
L153^‡^	FSHD2	>10	NM_015295.3(*SMCHD1*):c.[4347-129A>G];[=] on srWGS; FSHD2 methylation pattern on BSS	In *cis* D4Z4 duplication (4qA)	FSHD2	-	NM_015295.3(*SMCHD1*):c.[4347-129A>G];[=]	FSHD2
L154^‡^	FSHD2	>10	NM_015295.3*(SMCHD1*):c.[4347-129A>G];[=] on SS; FSHD2 methylation pattern on BSS	In *cis* D4Z4 duplication (4qA)	FSHD2	-	NM_015295.3(*SMCHD1*):c.[4347-129A>G];[=]	FSHD2
L438^‡^	FSHD2	>10	Assumed to have same *SMCHD1* variant as daughters (L153, L154)	In *cis* D4Z4 duplication (4qA)	FSHD2	-	NM_015295.3(*SMCHD1*):c.[4347-129A>G];[=]	FSHD2
L439	FSHD1 + 2	7	NM_015295.3(*SMCHD1*):c.2604-10_2615del on exome sequencing	7 (4qA)	FSHD1 + 2	158.63 (4.32)	NM_015295.3(*SMCHD1*):c.[2604-10_2615del];[=]	FSHD1 + 2

*BSS* bisulphite sequencing, *FSHD* facioscapulohumeral muscular dystrophy, *ONT LRS* Oxford Nanopore Technologies long-read sequencing, *S.D.* standard deviation, *SB* Southern blot, *SS* Sanger sequencing, *srWGS* short-read whole genome sequencing, *SSLP* short sequence length polymorphism.

^*^Southern blot of *Eco*RI/*Bln*I double-digested DNA separated by pulse-field gel electrophoresis hybridised with p13E-11 probe; for cases L138, L139 and L117, Southern blot of *Hind*III-digested DNA hybridised with a 4qA probe

^†^siblings.

^‡^siblings (L153, L154) and mother (L438).

In addition to D4Z4 contractions, FSHD1 cases showed the expected methylation profile with hypomethylation of the distal-most D4Z4 unit on the contracted 4qA D4Z4 repeat array compared to participants without FSHD (median ± median absolute deviation [MAD] methylation fraction 29% ± 8.29% vs. 68% ± 7.58%, respectively) and normal methylation of the distal-most D4Z4 unit on the other, non-contracted allele (Fig. 2e). The distal-most D4Z4 unit median methylation fraction ± 2 × MAD yields distinct, non-overlapping ranges for the two groups: 12.42–45.58% for FSHD1 vs. 52.84–83.16% for non-FSHD (Fig. 2e). In the family of three patients with FSHD2, in addition to detecting a previously identified intronic *SMCHD1* variant (Supplementary Fig. 6a) that had been shown to be pathogenic by an external research laboratory, we observed distal D4Z4 unit hypomethylation on both 4q alleles (Fig. 2e); median methylation fraction fell within the previously identified median ± 2 × MAD range seen in FSHD1 patients. In a case of FSHD1 + 2 (L439), our assay identified both the known 7-copy D4Z4 contraction on a 4qA haplotype and *SMCHD1* variant (Supplementary Fig. 6b). Both 4q D4Z4 alleles were hypomethylated, similar to FSHD2 patients, but the contracted 4q D4Z4 allele was more severely hypomethylated than the non-contracted allele (Fig. 2e, Supplementary Fig. 6c), highlighting the compounding effects of the two pathogenic variants in this participant. Our data also recapitulate known relationships between D4Z4 copy number, D4Z4 methylation level and age of symptom onset in FSHD1 (Supplementary Fig. 7a–c)^5,21^.

### Enhanced genetic and epigenetic characterisation of FSHD

Our new FSHD analysis method provided a more complete, high-resolution survey of FSHD loci than previously possible, resulting in several new, revoked and/or refined diagnoses and new genetic insights amongst our cohort.

**Fig. 3:**
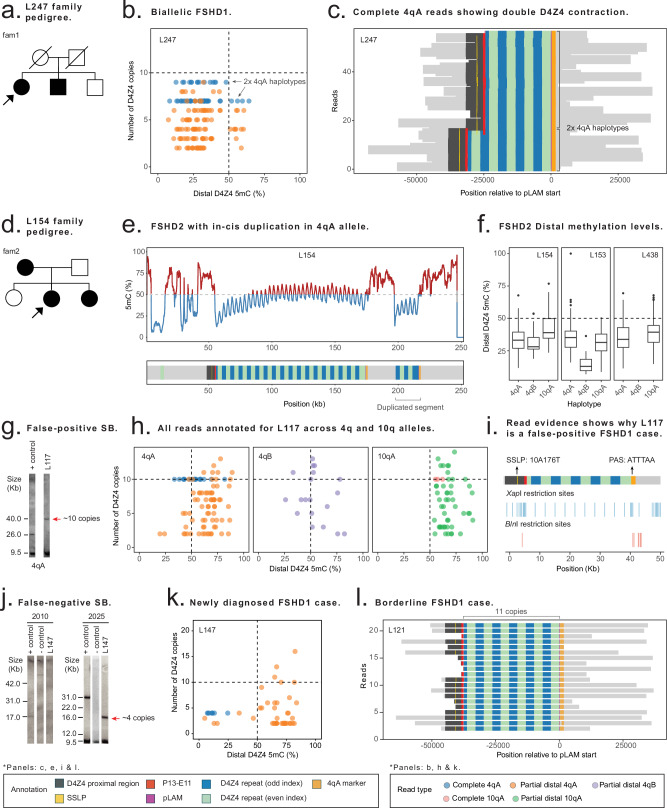
Targeted Oxford Nanopore Technologies long-read sequencing (ONT LRS) clarifies diagnoses in complex facioscapulohumeral muscular dystrophy (FSHD) cases. **a** Pedigree for cases L246 and L247. **b** Per-read 4qA D4Z4 copy number and distal-most D4Z4 unit methylation fraction plot for case L247. **c** Pile-up of complete 4qA reads from case L247 aligned to the pLAM sequence. **d** Pedigree of cases L154, L153 and L438. **e** 5mC methylation profile across the Hifiasm-assembled D4Z4 array duplication contig (bottom schematic), coloured by ‘higher’ (red) or ‘lower’ (blue) methylation with respect to an arbitrary 50% methylation value (dashed line). **f** Boxplots of distal-most D4Z4 unit methylation fraction in cases L154, L153, and L438. The dashed line represents an arbitrary 50% methylation value. Boxplots show the median (centre line), first and third quartiles (hinges), and most extreme values within 1.5 × the interquartile range of the hinges (whiskers). The number of reads contributing to each boxplot varied between samples (12 to 242). **g** Historical ‘positive’ 4qA-specific Southern blot (SB) in case L117. **h** Per-read D4Z4 copy number and distal-most D4Z4 unit methylation fraction plot for case L117 across all allele haplotypes showing 10-copy D4Z4 array reads apparently mapping to both 4qA and 10qA D4Z4 loci. **i** 10-copy D4Z4 array read schematic from case L117 annotated with key features. Presence of 4q-like (*Xap*I-sensitive, *Bln*I-resistant) D4Z4 units but a 176 bp sequence length polymorphism (SSLP) and non-canonical ATTTA polyadenylation signal (PAS) characterises 10A176T, an uncommon 10qA allele. **j** Historical ‘negative’ linear gel electrophoresis SB and, following diagnosis by ONT LRS, ‘positive’ pulsed-field gel electrophoresis SB in case L147. **k** Per-read 4qA D4Z4 copy number and distal-most 4qA D4Z4 unit methylation fraction plot for case L147. **l** Pile-up of complete 4qA reads from case L121 aligned to the pLAM sequence. Symbols for (**a**, **d**): square, male; circle, female; filled-in, affected; arrow, proband; diagonal line, deceased. For (**b**, **h**, **k**), dashed lines represent an arbitrary 50% methylation value (vertical) and the 10-copy diagnostic threshold for FSHD1 (horizontal). For (**g**, **j**), each set of control and proband lanes was run in non-adjacent lanes of a gel and have been rearranged to remove irrelevant lanes. Source data for (**b**, **e**–**h**, **j**, **k**) are provided in a Source Data file.

In cases L246 and L247 (Fig. 3a), siblings with FSHD1, previous SB results not only identified two D4Z4 contraction lengths, but these lengths were also apparently discordant between siblings (7 & 9 D4Z4 repeats in L246; 6 & 8 D4Z4 repeats in L247). Additionally, results of previous BSS performed by an external research laboratory^22^ were also discordant, being reported as consistent with FSHD1 in L246 and FSHD2 in L247. LRS data resolved these discrepancies, showing that both siblings harboured biallelic, hypomethylated D4Z4 contractions of 6 and 8 repeats, both on 4qA alleles (Fig. 3b, c). The discrepancy in D4Z4 repeat number on SB between the siblings presumably relates to the known error margin of SB. The misdiagnosis of L247 as having FSHD2 by BSS and the discrepancy between the BSS results of the siblings may reflect the inability of BSS to assess allele-specific methylation levels in the presence of two 4qA alleles and/or that the diagnostic methylation thresholds of the BSS assay were not calibrated for the uncommon occurrence of biallelic FSHD1 D4Z4 contractions. Complex cases such as these highlight the utility of our assay, which is able to concurrently assess allele-specific D4Z4 contraction size, 4q haplotype and D4Z4 methylation, compared to typical SB or BSS assays that provide only partial genetic or epigenetic characterisation of the D4Z4 locus.

In cases L153, L154, and L438, two daughters and a mother with FSHD2 (Fig. 3d), in addition to detecting the known *SMCHD1* variant and FSHD2 methylation pattern, LRS identified that all three also harboured permissive 4qA alleles containing a duplicated D4Z4 repeat array, with a duplicated D4Z4 segment that was 5-copies in length and hypomethylated (Fig. 3e, f). Such in *cis* D4Z4 array duplications have been reported previously in FSHD, with the size of the individual duplicated D4Z4 arrays thought to determine whether the duplication by itself causes autosomal dominant FSHD1 or whether an additional variant in *SMCHD1* is required, causing digenic FSHD2^23^. Using an empirical formula determined by Lemmers et al.^24^, the in *cis* duplication event in the family studied here is predicted to require an additional FSHD2 variant to cause FSHD.

Our analysis also clarified several cases where SB results either led to incorrect diagnoses or were inconclusive. Case L117 previously had SB showing a 10-copy D4Z4 contraction apparently located on a permissive 4qA allele and was diagnosed with FSHD1 (Fig. 3g). However, our LRS data unexpectedly showed this participant did not have a D4Z4 contraction on a 4qA allele nor distal D4Z4 hypomethylation (Fig. 2e), but rather a 10-copy D4Z4 contraction on a rare 10qA haplotype known as 10A176T (Fig. 3h, i). The 10A176T haplotype, present in 2.5% of the general European population, contains chromosome 4-like restriction enzyme sites and is theorised to be derived from an ancestral chromosome 4 to chromosome 10 translocation event^25,26^. The presence of a D4Z4 contraction on this 10A176T allele appears to have generated a false-positive SB result in case L117. An alternative genetic myopathy diagnosis was not identified, but our results clearly indicate this participant did not have FSHD. Subsequent review of the clinical phenotype also revealed the presence of clinical features atypical for FSHD (symmetric weakness, presence of brisk reflexes), further supporting the inaccuracy of the previous FSHD diagnosis.

Case L147, recruited as an unsolved Group 2 case, had a pattern of weakness consistent with FSHD but SB (following linear gel electrophoresis; LGE) had previously shown the absence of a D4Z4 repeat contraction (Fig. 3j). Our LRS assay showed this participant harboured a distally-hypomethylated 4-copy D4Z4 contraction on a permissive 4qA allele, consistent with FSHD1 (Fig. 3k). Subsequently, repeat SB (following pulsed-field gel electrophoresis; PFGE) was performed, confirming a contraction of 16 kb (~4 D4Z4 repeats) (Fig. 3j). The cause for the initial negative SB may relate to technical limitations of LGE-SB compared to PFGE-SB. As a result of the new diagnosis, case L147 and his family were offered genetic counselling specific to the autosomal dominant inheritance pattern of the disease, which was not previously apparent as both parents were unaffected, and referral for participation in specific FSHD1 clinical trials, which require a secure genetic diagnosis for enrolment.

Case L121 had a typical FSHD phenotype but a previous SB showed a 4qA allele with an estimated 11 D4Z4 units, which is not thought to cause FSHD1. Given the known error margin for SB D4Z4 array sizing, it was suspected that the participant might actually have a 10-copy D4Z4 array and the patient was considered to have ‘possible FSHD1’. However, our LRS data confirmed an 11-copy D4Z4 array on a permissive 4qA haplotype, supporting the original SB findings (Fig. 3l). The distal-most D4Z4 unit of the 11-copy 4qA D4Z4 array was mildly hypomethylated (Fig. 2e), while the other 4q allele (which was a 4qB haplotype) was not hypomethylated. This pattern and the absence of any disease-causing variants in *SMCHD1*, *DNMT3B* and *LRIF1* argued against a diagnosis of FSHD2. No alternative genetic explanation for the participant’s FSHD-like phenotype was identified. We then sequenced the brother of L121, who also presented with an FSHD-like pattern of muscle weakness, and again identified an 11-copy 4qA D4Z4 array with mild distal hypomethylation (Supplementary Fig. 7d). Based on current FSHD diagnostic criteria, these siblings remain genetically undiagnosed. Whether 11-copy D4Z4 repeat 4qA alleles, which are imprecisely measured using SB and currently considered non-pathogenic, may actually occasionally cause FSHD1, perhaps with incomplete penetrance, requires further study. LRS analysis and careful phenotyping of a larger number of cases with 11-copy D4Z4 repeats may help to clarify this issue.

### Enhanced genetic and epigenetic characterisation of OPDMs

**Fig. 4:**
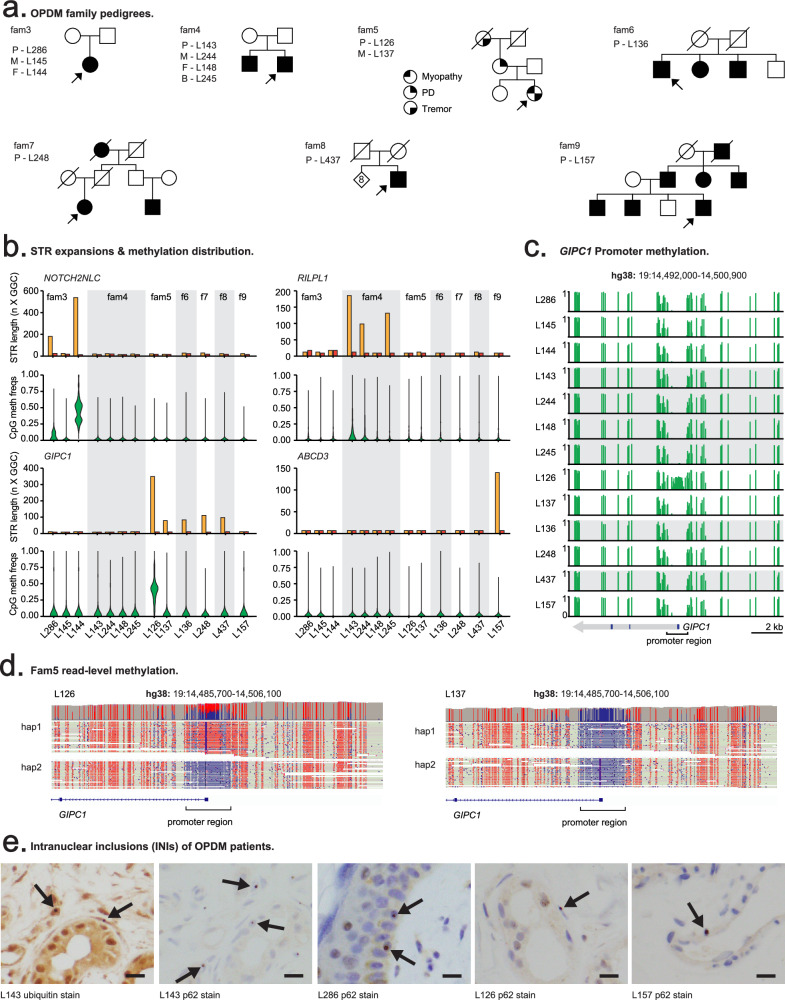
Targeted Oxford Nanopore Technologies long-read sequencing (ONT LRS) characterises the genetic and epigenetic architecture of oculopharyngodistal myopathy (OPDM). **a** Pedigrees of OPDM families diagnosed by ONT LRS. Symbols: square, male; circle, female; filled-in, affected; arrow, proband; diagonal line, deceased. IDs for the proband (P), mother (M), father (F) and brother (B) are shown for family members who underwent ONT LRS. **b** GGC•CCG short tandem repeat (STR) lengths and CpG methylation fractions across *NOTCH2NLC*, *RILPL1*, *GIPC1*, and *ABCD3* in individuals from OPDM families. In the top panels, allele-specific STR repeat lengths are represented separately (yellow = haplotype 1, orange = haplotype 2). In the bottom panels, violin plots represent the density of per-read methylation values. **c** CpG methylation across the *GIPC1* promoter in all OPDM family members. Vertical bars indicate methylation levels at different CpG sites. **d** Genome browser view of *GIPC1* read-level methylation in case L126, a participant with OPDM2 due to a *GIPC1* (GGC)_217-570_ STR expansion, and case L137, the mother of case L126, who has Parkinson’s disease but no myopathy and a *GIPC1* (GGC)_76-85_ STR expansion. Reads are phased into haplotypes 1 and 2. CpG sites are coloured by methylation state (red = methylated, blue = unmethylated). **e** Anti-ubiquitin and anti-p62 immunohistochemistry of skin biopsies showing intranuclear inclusions (arrows) in a range of cell types (epithelium, adipocytes, fibroblasts, pericytes) in OPDM cases. Scale bar = 20 μm. Source data for (**b**) are provided in a Source Data file.

Out of the 11 new diagnoses established by our LRS assay, 7 were OPDM, a group of disorders caused by non-coding GGC•CCG STR expansions, typically located in the 5′ untranslated region. Four patients were diagnosed with *GIPC1*-related OPDM (OPDM2) and one each with *NOTCH2NLC*-related (OPDM3), *RILPL1*-related (OPDM4) and *ABCD3*-related (OPDM5) OPDM (Table 3; Fig. 4, Supplementary Fig. 8a). Skin biopsies were subsequently performed in 4 of these cases, and all showed the presence of p62- and ubiquitin-positive intranuclear inclusions (INIs), typical of OPDM (Fig. 4e; Supplementary Methods). Although our analysis approach is suitable for identifying novel STR motifs and/or interruptions, all expanded alleles detected here were canonical ‘GGC’ repeats lacking interruptions. Our results demonstrate that OPDM may be a major contributor to currently undiagnosed genetic myopathies.

**Table 3:** New myopathy diagnoses identified by ONT LRS

Case ID	Clinical phenotype	Age of onset range (Years)	Disease duration (Years)	ONT LRS results	Final diagnosis
L236	Limb girdle weakness	31–35	8	NM_000426.4(*LAMA2*):c.[2749+4_2749+15del];[2749+4_2749+15del]	LGMD R23
L323	Limb girdle weakness	21–25	28	NM_000070.3(*CAPN3*):c.[1435A>G];[2440-75C>G]	LGMD 2A/R1
L155	Ptosis, limb girdle weakness	41–45	2	NM_080916.3(*DGUOK*):c.[708-1G>A];[759T>G] and multiple large mtDNA deletions	*DGUOK*-related multiple mtDNA deletion myopathy
L286	Oculopharyngodistal & facial weakness	16–20	14	*NOTCH2NLC* 5′ UTR (GGC)_130–621_ expansion	OPDM3
L143	Oculopharyngodistal & facial weakness	26–30	9	*RILPL1* 5′ UTR (GGC)_191_ expansion	OPDM4
L126	Fascioscapuloperoneal weakness and essential tremor	31–35	3	*GIPC1* 5′ UTR (GGC)_217–570_ expansion, hypermethylated	OPDM2
L136	Oculopharyngodistal & facial weakness	46–50	19	*GIPC1* 5′ UTR (GGC)_85_ expansion	OPDM2
L248	Oculopharyngodistal & facial weakness	31–35	5	*GIPC1* 5′ UTR (GGC)_112_ expansion	OPDM2
L437	Facial, pharyngeal & distal limb weakness	56–60	10	*GIPC1* 5′ UTR (GGC)_99_ expansion	OPDM2
L157	Ptosis, mild facial weakness & ophthalmoplegia	21–25	19	*ABCD3* 5′ UTR (CCG)_170_ expansion	OPDM5
L147	Facioscapulohumeral weakness	1–5	38	4-copy D4Z4 contraction on a 4qA allele and FSHD1 methylation pattern	FSHD1

*FSHD* facioscapulohumeral muscular dystrophy, *LGMD* limb-girdle muscular dystrophy, *mtDNA* mitochondrial DNA, *ONT LRS* Oxford Nanopore Technologies long-read sequencing, *OPDM* oculopharyngodistal myopathy, *UTR* untranslated region.

Case L286 (fam3 in Fig. 4a) had a GGC expansion in *NOTCH2NLC* of variable length (range 130–621 repeats), exceeding the size of healthy controls (Supplementary Fig. 8a), and skin biopsy showed numerous p62/ubiquitin-positive INIs (Fig. 4e), consistent with a diagnosis of OPDM3. The participant’s father (L144), a Cook Island Māori participant who did not have myopathy, was found to harbour a very large GGC expansion, again with variable length (range 273–905). The variable length of the *NOTCH2NLC* STR expansions are consistent with somatic instability/mosaicism that has been described in some patients^27^. While the STR expansion and the surrounding *NOTCH2NLC* promoter region in L286 exhibited DNA methylation levels similar to non-OPDM3 participants and healthy controls, LRS identified that the same region in L144 exhibited allele-specific hypermethylation of the *NOTCH2NLC* promoter region (Fig. 4b and Supplementary Fig. 8b). This phenomena of asymptomatic parents harbouring extremely long ([GGC]_>400_), hypermethylated expansions in *NOTCH2NLC* having children with shorter, but still pathological, GGC expansions that are not hypermethylated, is well documented in *NOTCH2NLC*-related OPDM3 and neuronal intranuclear inclusion disease (NIID)^27,28^. Contraction of the GGC STR expansion size across generations occurs more commonly following paternal inheritance^27^, as was the case here. Case L286 and her asymptomatic father both also harboured a heterozygous expansion of the normal 29–31 × 99 bp VNTR in exon 5 of *PLIN4* to 39 × 99 bp (Supplementary Fig. 9). *PLIN4* VNTR expansions have been described to cause a vacuolar myopathy, often with prominent distal weakness^29^. The significance of the VNTR expansion in this family is unclear given that OPDM3 better accounts for the phenotype and that the father is asymptomatic. *PLIN4* alleles expanded to at least 38 × 99 bp were also detected among population reference data from the 1000 Genomes Project Long-read Sequencing Consortium (1KGP-LRSC)^30^, suggesting incomplete penetrance (Supplementary Note 2).

Cases L126, L136, L248 and L437 (fam5, fam6, fam7 and fam8 in Fig. 4a, respectively) were found to have GGC STR expansions (217–570, 85, 112 and 99 repeats, respectively) in *GIPC1*. The previously reported pathogenic range for *GIPC1* GGC expansions was 73–164, indicating that cases L136, L248 and L437 can be confidently diagnosed with OPDM2^31^. Similar to *NOTCH2NLC*, extremely long, hypermethylated STR expansions in *GIPC1* ([GGC]_>500_) have been found in asymptomatic fathers of OPDM2 patients who themselves have typical *GIPC1* repeat expansion lengths^31^. We observed allele-specific DNA hypermethylation of the large variable-length (GGC)_217–570_ expansion and surrounding *GIPC1* promoter region in L126, compared to all other samples (Fig. 4b–d). Based on the clinical presentation and presence of INIs on skin biopsy (Fig. 4e), we diagnosed case L126 with OPDM2. This would seem to expand the pathogenic STR length for *GIPC1* to at least 217 repeats and demonstrates that OPDM may still manifest in cases of hypermethylated GGC repeats. Interestingly, in 1KGP-LRSC population reference data we identified a single individual (HG00653) with an even larger *GIPC1* expansion (549 copies) also exhibiting allele-specific hypermethylation, assumed to be non-symptomatic (Supplementary Fig. 8a). The mother of case L126 (L137), did not have myopathy, but did have typical levodopa-responsive Parkinson’s disease and was found to harbour a non-hypermethylated (GGC)_76–85_ expansion in *GIPC1* (Fig. 4b) but skin biopsy did not identify any INIs. This highlights the instability of the *GIPC1* repeat across generations and also aligns with recent reports linking shorter *GIPC1* STR expansions with Parkinson’s disease^32,33^. The *GIPC1* STR expansions in cases L248 (New Zealand Māori ancestry) and L437 (mixed New Zealand Māori, Tongan and European Australian ancestry) occurred within a haplotype, consisting of 7 SNPs in or near the *GIPC1* locus, that was previously identified in 100% of a Japanese cohort of 10 OPDM2 patients (Supplementary Table 1)^34^. This haplotype was present in only 11.58% of 2,324 alleles in a Japanese reference population and was thus suggested to be in linkage disequilibrium with the *GIPC1* STR expansion. Cases L126 (European Australian) and L136 (Chinese) only harboured 6 of the 7 SNPs on their respective *GIPC1* STR expansion alleles, perhaps suggesting that, compared to the *GIPC1* allele of the two participants with New Zealand Māori ancestry, these alleles may have a more distant shared ancestral origin with the Japanese OPDM2 STR expansion allele (Supplementary Table 1).

Case L143 (fam4 in Fig. 4a) was found to have a (GGC)_191_ expansion in *RILPL1* consistent with OPDM4 and skin biopsy identified the typical INIs (Fig. 4e, Supplementary Fig. 8a). His brother (L245), who had ocular and facial weakness without limb or pharyngeal involvement and had been labelled as having chronic progressive external ophthalmoplegia (CPEO), a type of mitochondrial myopathy, was recruited to this study. LRS showed the brother harboured a (GGC)_131_ expansion in *RILPL1* and no pathogenic variants in any of the nuclear or mtDNA genes that cause CPEO, indicating he also had OPDM4. Their mother (L244), who was of Papua New Guinean and Ni-Vanuatu ancestry, did not have myopathy or any other neurologic features but was found to harbour a (GGC)_98_ expansion in *RILPL1*, falling short of the previously reported pathogenic size range of 130–197^35^. The mother’s STR allele represents an intermediate range expansion that does not cause symptoms in itself but is prone to further expansion in subsequent generations, as seen in this family. The *RILPL1* STR and promoter regions were not hypermethylated in any of these individuals.

Case L157 (fam9 in Fig. 4a), only presented with ptosis and subtle facial weakness and ophthalmoplegia but had a family history of an autosomal dominant OPDM phenotype. He was identified to have a (CCG)_170_ expansion in *ABCD3* (Fig. 4b, Supplementary Fig. 8a) and was subsequently identified to be related to one of the recently published European Australian OPDM5 families^36^.

### Further genetic diagnoses in previously unsolved cases

**Fig. 5:**
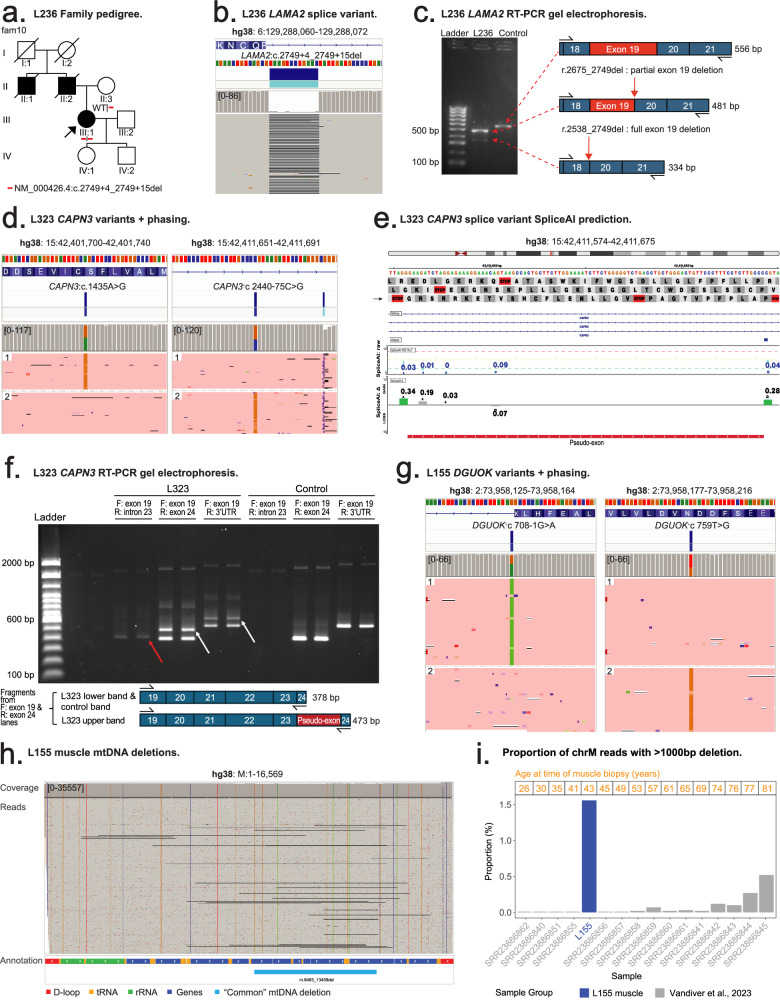
Additional new genetic diagnoses made using Oxford Nanopore Technologies long-read sequencing (ONT LRS). **a** Pedigree of case L236. Symbols: square, male; circle, female; filled-in, affected; arrow, proband; diagonal line, deceased. **b** Genome browser view of a homozygous intronic *LAMA2* variant, c.2749+4_2749+15del, in case L236. **c** *LAMA2* reverse transcription polymerase chain reaction (RT-PCR) confirmed absence of a normal splice product and generation of two novel splice products (in-frame truncation and complete skipping of exon 19) in case L236 but not in a control. RT-PCR was run twice with concordant results. **d** Genome browser view of *CAPN3* variants, c.1453A>G and c.2440-75C>G, in *trans* in case L323. **e** SpliceAI predicts *CAPN3*:c.2440-75C>G may create a new donor splice site (Δ0.28) one base-pair downstream and also activate an upstream cryptic acceptor site (Δ0.33), together resulting in inclusion of a 95 bp pseudoexon into the *CAPN3* transcript between the penultimate and final exons. The new pseudo-exon is predicted to translate into a novel 20 amino acid sequence (arrow) followed by a premature stop codon. **f** *CAPN3* RT-PCR using two combinations of forward (F) and reverse (R) primers identified the presence of a novel splicing product (white arrows) ~95 bp longer than the wild-type product in case L323 but not in a control. RT-PCR using a reverse primer to intron 23 identified a product in case L323 (red arrow) but not in the control. RT-PCR was run twice with concordant results. **g** Genome browser view of biallelic *DGUOK* variants, c.708-1G>A and c.759T>G, in case L155. **h** Genome browser view of multiple mtDNA deletions (black lines) in muscle-derived DNA from case L155. The location of a “common” 4.77 kb mtDNA deletion (m.8483_13459del) is indicated for reference. **i** Proportion (%) of muscle-derived mtDNA reads containing a > 1000 bp deletion out of the total number of mtDNA reads > 1000 bp in length from case L155 and 15 healthy individuals from Vandiver et al. Samples are ordered by increasing age at the time of muscle biopsy. Source data for (**c,** **f,** **i**) are provided in a Source Data file.

In addition to OPDM and FSHD, our LRS assay led to three new genetic diagnoses involving standard small variants that were missed or incompletely characterised during previous testing. In case L236, who had a limb girdle pattern of myopathy and an apparently autosomal dominant pattern of inheritance (Fig. 5a), our LRS assay identified a homozygous intronic variant in *LAMA2*, NM_000426.4:c.2749+4_2749+15del, affecting the extended donor splice site of intron 19 (Fig. 5b). Sanger sequencing confirmed the homozygous status of the variant in the proband and that the proband’s mother was a heterozygous carrier (Supplementary Fig. 10a). The proband’s affected father and paternal uncle were deceased and unable to be tested. The reason this variant was not identified by the previous NGS targeted gene panel, which was performed by an external laboratory and included *LAMA2*, is unclear but may relate to inadequate coverage of this intron-exon boundary. The variant (rs2482293091) is present in one South Asian individual on gnomAD v4.1.0 and has previously been reported as a VUS in ClinVar (RCV003133994.3 and RCV004577033.1). It was predicted by SpliceAI to result in loss of the donor site (Δ0.99) and use of a cryptic donor splice site 78 bp upstream (Δ0.55; Supplementary Fig. 10b). Targeted RT-PCR from participant skin-derived fibroblasts (Supplementary Methods) confirmed absence of the normal *LAMA2* splicing product and presence of two aberrant splicing products (Fig. 5c). The predominant product resulted from use of the cryptic upstream donor site resulting in an in-frame 75 bp deletion-insertion, p.Gly892_Pro917delinsAla – this same mis-splicing event, alternatively labelled as p.Glu892Ala del893_917, was previously identified in a patient with a different variant affecting the same donor splice site ('c.2750+2insT')^37^. Complete skipping of exon 19 was the other, less frequent, mis-splicing event, which would result in a frameshift and presumed nonsense-mediated decay of the transcript. We considered this intronic *LAMA2* variant to be likely pathogenic (PM2, PM3_supporting, PVS1_strong [RNA]) and diagnosed case L236 with *LAMA2*-related limb-girdle muscular dystrophy (LGMD) type R23. Review of a previous muscle MRI and subsequent brain MRI identified features consistent with LGMD R23 (Supplementary Fig. 10c, d)^37^. The apparent dominant inheritance in this family was presumed to reflect pseudodominance, a phenomenon where an autosomal recessive disorder appears to follow an autosomal dominant pattern of inheritance. Pseudodominance has previously been described in *LAMA2*-related muscular dystrophy^37^. The homozygosity of the variant in L236, in the absence of consanguinity, was attributed to identity by descent – both parents’ families came from the same small city in India. As a result of the new diagnosis, the patient and her family were provided with genetic counselling pertaining to autosomal recessive inheritance, overriding the previous erroneous assumption of autosomal dominant inheritance.

In case L323, a patient with a LGMD phenotype, prior NGS using a targeted gene panel (including *CAPN3*) through an external laboratory was reported as not showing any causative genetic variants. Our ONT LRS assay identified a known pathogenic missense variant (NM_000070.3:c.1435A>G) and an intronic VUS (NM_000070.3:c.2440-75C>G) in *CAPN3* that were in *trans* (Fig. 5d). The intronic variant was likely not identified by the prior targeted gene panel analysis due to its intronic location, while the missense variant may have been identified but perhaps not reported as it is not one of the few *CAPN3* variants known to cause autosomal dominant disease by itself. The intronic variant is absent from gnomAD v4.1.0 and has previously been reported as a VUS in ClinVar (RCV002225229.2) in an Australian myopathy patient along with another pathogenic missense variant (different to that seen in L323) and muscle biopsy findings consistent with a calpainopathy. SpliceAI suggested the variant may alter splicing (Fig. 5e) and targeted RT-PCR of blood from L323 (Supplementary Methods) confirmed that it resulted in inclusion of a 95 bp pseudoexon from intron 23 into the *CAPN3* transcript, between the penultimate and final exons (Fig. 5f, Supplementary Fig. 11a). The pseudoexon contains a premature termination codon located <50 bp from the last (pseudo)exon-exon boundary and is thus predicted to escape nonsense-mediated decay, resulting in a C-terminally modified protein where the final 8 amino acids, normally encoded by the final exon, are replaced by a novel 20 amino acid sequence, p.(Trp814Glyfs20), encoded by the pseudoexon (Fig. 5e). Canonical splice site variants of this splice junction (e.g. c.2440-1G>A) are known to cause disease through loss of the C-terminal 8 amino acids (p.Trp814*), part of the penta-EF-hand domain^38^. This penta-EF-hand domain is important for CAPN3 oligomierisation and titin-binding^39^. Therefore, we considered the NM_000070.3:c.2440-75C>G as likely pathogenic (PM2, PM3, PVS_moderate [RNA]) and diagnosed case L323 with *CAPN3*-related LGMD 2A/R1. A pattern of muscle involvement consistent with LGMD 2A/R1 was present on muscle MRI (Supplementary Fig. 11b, c)^40^.

In case L155, a patient with ptosis, myopathy and a muscle biopsy showing features of a mitochondrial myopathy (COX-negative and ragged red fibres), prior NGS identified a likely pathogenic splice acceptor variant (NM_080916.3:c.708-1G>A) and a novel missense VUS (NM_080916.3:c.759T>G, p.Asn253Lys) in *trans* in *DGUOK*. Biallelic pathogenic variants in *DGUOK* cause a mitochondrial disorder with secondary mtDNA depletion and/or deletions (typically identified in muscle DNA)^41,42^. The missense VUS was absent from gnomAD and had inconclusive in silico pathogenicity scores (REVEL 0.4, AlphaMissense 0.54, MetaLR 0.78). It is located between two nearby likely pathogenic/pathogenic missense variants, c.749T>C (p.Leu250Ser) and c.763G>T (p.Asp255Tyr)^43,44^. ONT LRS on DNA from participant blood and skeletal muscle (Supplementary Methods) identified the two *DGUOK* variants in *trans* (Fig. 5g) in both samples and the presence of multiple large mtDNA deletions in muscle only (Fig. 5h, Supplementary Fig. 12). Although mtDNA deletions accumulate in normal muscle with aging^45,46^, the proportion of mtDNA reads containing a large (>1000 bp) deletion in L155 muscle (1.6%; 1542/99010 reads) was more than 3-fold higher compared to that seen in LRS data from healthy human skeletal muscle (<0.5%; Fig. 5i) sourced from the National Center for Biotechnology Information Sequence Read Archive^47,48^. No potentially disease-causing variants were identified in any of the other nuclear genes known to cause multiple mtDNA deletion syndromes (e.g., *TYMP*, *TK2*, etc.). Although the missense variant remains a VUS using strict ACMG criteria (PM2, PM3, PP4-supporting), L155 was diagnosed with a multiple mtDNA deletion mitochondrial myopathy syndrome, which we considered likely due to the biallelic *DGUOK* variants.

## Discussion

Our targeted ONT LRS assay and analysis framework provides thorough characterisation of the wide range of genetic and epigenetic variation that causes genetic myopathies. We identified a new genetic diagnosis in more than a third of patients who remained undiagnosed after previous standard genetic testing. FSHD and OPDM were the most common new diagnoses that were either missed or misdiagnosed by previous clinical genetic testing. Our assay also improved the precision of previously known genetic diagnoses and identified several false-positive and false-negative diagnoses that could be overturned using LRS data. The new diagnoses achieved in this study resolved prolonged diagnostic odysseys, spanning years to decades (mean disease duration of 14 years for the *n* = 11 newly solved cases) and facilitated disease-specific management (e.g., genetic counselling, clinical trial participation, etc.). We anticipate further improvements in diagnostic yield as increasing availability of LRS population reference data from initiatives such as the 1KGP LRS Consortium^30^ facilitates improved filtering and functional annotation of repetitive, structural and complex variations – an important unsolved challenge. In addition to improved diagnosis, the high resolution and comprehensive nature of LRS data also shed new light on the molecular basis of disease. For example, our FSHD analysis method observed the compounding effects of ‘double’ variants in a case of FSHD1 + 2. Similarly, our results help to refine the observed pathogenic range for STR expansions in patients with OPDM and characterise the influence of DNA methylation in this disorder.

We showed that LRS is capable of comprehensively assessing the complex genetic and epigenetic architecture of FSHD. Our open source software package, d4z4ling, which is compatible with any LRS data type, provides accurate D4Z4 repeat copy number assessment, single-molecule methylation profiling, and detailed haplotype resolution. While ONT-based methods have previously been applied to FSHD1^49–52^, our approach is the first to demonstrate the ability of adaptive targeted LRS to characterise FSHD1, FSHD2, FSHD1 + 2 and all other myopathic loci concurrently, simplifying the diagnostic process in the face of genotypic and phenotypic heterogeneity. The concurrent genetic and epigenetic assessment of FSHD allows our assay to detect and also validate VUSs relevant to FSHD. For example, identification of a novel variant in an FSHD2-related gene (e.g., *SMCHD1*) along with a pattern of D4Z4 hypomethylation consistent with FSHD2 may provide variant validation without the need for further investigations. Our assay also facilitates better examination of diagnostic edge cases – although our identification of two siblings with an FSHD phenotype and 11-copy 4qA D4Z4 repeat arrays with D4Z4 hypomethylation is clearly insufficient to challenge the well-established FSHD1 diagnostic threshold of ≤10 D4Z4 repeats, it does highlight the need to study further cases with D4Z4 contractions close to this threshold to better understand FSHD disease pathogenesis.

OPDM accounted for more than half of the new diagnoses identified by our LRS assay, which is perhaps unsurprising given the current lack of clinically accredited genetic testing options for any of the OPDM STR expansions in Australia. The OPDM phenotype was described in the 1970s, but the causative GGC•CCG STR expansions were only identified in the last 7 years^31,35,36,53,54^. These STR expansions were initially described predominantly in East Asian cohorts, whereas the most recently identified locus (*ABCD3*) was found in OPDM patients of European ancestry^36^. Our LRS assay was not only able to concurrently identify the presence of STR expansion across multiple loci but also characterise the methylation status of these loci, characterise mosaicism of the STR expansion length, and phase the STR expansions with surrounding SNPs to identify common haplotypes. Our results expand knowledge of the ethnic backgrounds in which different OPDM types occur. The finding of a *NOTCH2NLC* GGC expansion causing OPDM3 that originated from a Cook Island Māori family extends the recent description of *NOTCH2NLC* GGC expansion-related NIID in this ethnic population^55^. To date, *GIPC1*-related OPDM2 has only been reported in East Asian patients. For the first time, we report *GIPC1* OPDM2 in patients of European Australian and New Zealand Māori ancestry. The sharing of uncommon SNPs with a Japanese OPDM2 haplotype suggests the possibility of a common ancestral haplotype that may predispose to expansion of the *GIPC1* STR. *RILPL1* GGC expansions, which cause OPDM4, have only ever been described in OPDM patients of Chinese ancestry and were even absent in a large Japanese OPDM cohort^56^. Here, we report the finding of OPDM4 in a family of mixed Papua New Guinean and Ni-Vanuatu ancestry. An OPDM phenotype was previously reported in a set of Papua New Guinean siblings, many years prior to identification of the causative STR expansions^57^; in light of our findings, it is interesting to speculate whether that family may have had OPDM4. Similarly, it would be of interest to determine if ‘Simbu ptosis’, a familial disorder apparently endemic to the Eastern Highlands of Papua New Guinea, consisting of myogenic ptosis that may be accompanied by ophthalmoplegia, facial weakness, nasal voice and foot drop (all features of OPDM)^58^, also represents OPDM4.

Despite our approach yielding clear benefits in terms of improved molecular characterisation of genetic myopathies, our study has several limitations. The use of adaptive targeted ONT LRS, compared to whole-genome LRS (ONT or PacBio), represents a 2–4-fold more cost-effective assay on a per participant basis, but is restricted to the programmed target loci. This limits detection of SVs and CNVs to those fully contained within a given target region, and precludes discovery of new myopathy genes or analysis of genes identified subsequent to the time of sequencing. Fortunately, unlike traditional targeted NGS panels, which require redesign of molecular probes or primers used for target enrichment, the in silico panel used to guide our ONT adaptive sampling assay can be updated simply by adding new genomic coordinates to a digital file. For example, we were able to rapidly add in the *ABCD3* loci during the course of our study following its identification as the cause for OPDM5 in 2024^36^. Nonetheless, whole–genome LRS may be preferable in some contexts, although we note that obtaining sufficient read length and read depth for reliable diagnosis of FSHD may become cost-prohibitive. Next, pre-study diagnostic workup varied widely between participants in Group 2 of our cohort (e.g., some had a targeted NGS panel, others had prior exome or genome NGS, etc.), making it difficult to precisely quantify the added diagnostic yield of ONT LRS on top of each of these individual NGS assays. However, this situation reflects the heterogeneity of a real-world cohort of unsolved genetic myopathy patients and here we have shown that ONT LRS increases overall diagnostic yield and accuracy compared to the collective group of ‘standard’ genetic testing methodologies. Finally, data from previously diagnosed patients in ‘Group 1’ were used during the development of our bioinformatics framework, most notably d4z4ling, and to demonstrate its performance. For this reason, we have not reported formal performance metrics such as sensitivity or specificity for d4z4ling, and rigorous analytical validation using an independent validation cohort is planned.

In conclusion, we describe a new targeted ONT LRS assay capable of assessing the full range of genetic and epigenetic variants implicated in the genetic myopathies and demonstrate its utility for diagnosis of patients with these conditions. Our method provides new insights into the complexity of this heterogeneous group of disorders, resolving challenging cases and revising erroneous diagnoses made by older molecular techniques. Our results demonstrate that clinical adoption of LRS will simplify diagnostic algorithms, improve diagnostic rates, and advance scientific understanding of the genetic myopathies.

## Methods

### Participant recruitment

This study followed local and national research governance regulations and was conducted in accordance with the Declaration of Helsinki regarding research involving human participants. The protocols for this study were approved by the St Vincent’s Hospital Human Research Ethics Committee (HREC/2019/ETH12538) and Royal Children’s Hospital Melbourne Human Research Ethics Committee (HREC/95179/RCHM-2023). All participants provided written informed consent, which included consent to publication of de-identified demographic and clinical data. Adult (age ≥18 years) participants were recruited from patients referred to Concord Repatriation General Hospital and Neuroscience Research Australia from March 2023 to September 2025. A positive control cohort, Group 1, consisted of patients with genetically confirmed myopathies specifically selected to represent a range of different genetic and epigenetic variant types. An undiagnosed cohort, Group 2, consisted of consecutively recruited participants that met the following criteria: (1) history, examination and investigations (creatine kinase, neurophysiology, muscle imaging and/or muscle biopsy) suggestive of and concordant for a myopathic process; (2) myopathy was the predominant neurologic phenotype; (3) the treating/referring clinician assessed that a genetic cause for the myopathy was more likely that an acquired aetiology; and (4) the participant remained genetically unsolved (or incompletely solved) after appropriate conventional clinical genetic testing as determined by the treating/referring clinician. Where relevant, family members of probands were recruited for segregation studies. Participants were not compensated for study participation. Reported pedigrees have been modified to protect participant anonymity while retaining instructive relationship information.

### Targeted ONT LRS

Peripheral blood samples were obtained and high-molecular weight (HMW) genomic DNA was extracted using the Nanobind CBB kit (PacBio, Cat# 102-301-900) or PanDNA kit (PacBio, Cat# 102-260-000). HMW genomic DNA was sheared to ~40–50 kb fragment size using the Diagenode Megaruptor 3 DNA shearing system (speed 27), treated with the Short-read Eliminator kit (PacBio, Cat# 102-208-300) to deplete fragments <10 kb, then visualised on an Agilent Femto Pulse using the Genomic DNA 165 kb Kit. ONT libraries were prepared from ~4 μg of sheared HMW genomic DNA using a ligation prep (SQK-NBD114.24). Between 1–3 participant samples were barcoded and pooled into one library and loaded on an ONT PromethION R10.4.1 flow cell (FLO-PRO114M) and sequenced on either a PromethION 2 Solo or PromethION 48 instrument.

Readfish^59^ (v0.0.10dev2) was used for live target selection or rejection utilising a custom panel of 331 loci associated with genetic myopathies (Supplementary Data 1). These loci were chosen based on a literature review performed in January 2023, focusing on genetic myopathic disorders. This was supplemented by a review of existing myopathy gene panels in PanelApp Australia^60^ (‘green’ and ‘amber’ genes) and external diagnostic genomic laboratories (Supplementary Data 2) – genes/loci that were not thought to produce a predominant myopathy were excluded from our custom panel. The *ABCD3* locus was added to the panel during the course of this study, such that it was sequenced in 26 out of the 53 proband samples. Gene targets were encoded relative to the T2T-chm13 (v2) reference genome and ReadFish was executed with the following configurations: config_name = ‘dna_r10.4.1_e8.2_400bps_5khz_fast_prom’; min_chunks = 0; max_chunks = 16; single_on = ‘stop_receiving’; multi_on = ‘stop_receiving’; single_off = ‘unblock’; multi_off = ‘unblock’; no_seq = ‘proceed’; no_map = ‘proceed’. Libraries were run for 72 hours, with nuclease flushes and library reloading performed at approximately 24-hour and 48-hour time points. Although the open-source software Readfish was used in our study, users may instead prefer ONT’s ‘adaptive sampling’ application provided within the MinKNOW interface, which is functionally comparable.

### LRS data processing

After completing a targeted sequencing run, raw ONT sequencing data were converted to BLOW5 format^61^ and base-called with Buttery-eel^62^ (a wrapper to enable BLOW5 input for ONT’s Dorado basecaller), using the ‘super-accuracy’ model (SUP) with 5mC methylation calling enabled. SUP basecalling was performed after the sequencing run on an external high-performance computing facility because live basecalling during sequencing may compete with Readfish (or MinKNOW adaptive sampling) for compute resources, negatively impacting target enrichment. Conversion of raw sequencing data to BLOW5 format prior to basecalling allows the user to enjoy the enduring performance advantages over ONT’s native POD5 format^63^. However, this is optional and the user may instead choose to basecall their POD5 files directly, using Dorado.

Base-called data was then processed using Pipeface (https://github.com/leahkemp/pipeface), a NextFlow pipeline for LRS data analysis. Pipeface has been tested on both whole-genome sequencing and adaptive sampling data, and is fully compatible with both ONT and PacBio long-read sequencing platforms. In this study, we used Pipeface to execute minimap2^64^ (v2.28-r1209) for alignment to the GRCh38 (hg38) reference assembly, Clair3^65^ (v1.0.9) and Deepvariant (v1.80) for calling small variants, sniffles2^66^ (v2.6.0) and cuteSV^67^ (v2.1.1) for calling SVs, WhatsHap^68^ (v2.3) for phasing, and minimod^69^ (v0.3.0) for DNA methylation profiling. Small variants and SVs were functionally annotated using the Variant Effect Predictor (VEP)^70^ (v112). All variants were filtered to exclude events outside the programmed target regions, including SVs not fully contained within a single target. We did not use a standalone CNV caller, meaning CNV detection was limited to large duplications or deletions identified by Sniffles or CuteSV.

### Secondary analysis and interpretation

Pipeface produces various standard output files (VCF, BAM, etc.) for downstream analysis. To facilitate variant visualisation, filtering, and interpretation, we used an R Shiny application, called PuzzleApp, designed for exploration of LRS data in standard formats. PuzzleApp is operated through a GUI with all parameters/filters adjusted flexibly on a case-by-case basis. As a general guide, small variants were prioritised for further consideration based on the following criteria: (i) those annotated in ClinVar with a clinical significance of ‘pathogenic’, ‘likely pathogenic’ or ‘uncertain’; (ii) variants predicted to cause a frameshift; (iii) missense variants with a REVEL score > 0.5 and classified as ‘pathogenic’, ‘likely pathogenic’, ‘uncertain’ or without an assigned clinical significance; (iv) variants with high or moderate impact that are similarly classified as ‘pathogenic’, ‘likely pathogenic’, ‘uncertain’ or lacking an assigned clinical significance in ClinVar and (v) variants predicted to alter splicing, defined as having a SpliceAI delta score > 0.2. All pre-selected variants were within MANE Select transcripts and either were not found in gnomADv4 or had an allele frequency of less than 0.01, resulting in a list of rare and potentially causative variants. SVs were prioritised based on: (i) intersection with protein-coding genes; (ii) predicted transcript-level consequences (e.g. exon overlap, intronic, untranslated region, upstream/downstream effects); (iii) absence from gnomAD SV database or presence at allele frequency of less than 0.01; (iv) absence of similar or identical SVs within 1KGP-LRSC reference data or present at <0.01 AF (i.e. <2/149 individuals at time of initial analysis); (v) identical or similar SVs found in ClinVar with a clinical significance of ‘pathogenic’, ‘likely pathogenic’ or ‘uncertain’. SV matching against gnomAD, 1KGP-LRSC and our own cohort was performed to detect identical or related variants using the following criteria: for DEL, INV and DUP, a minimum 50% reciprocal overlap was required; for INS, the distance between left breakpoints was required to be less than 50% of the length of the smaller insertion, and the length of the smaller insertion was required to be greater than 50% of the length of the larger insertion. Applying these filtering criteria returned an average of 7.55 ± 6.52 candidate SVs per patient, as summarised in Supplementary Data 3. We note that the criteria for prioritising variants and SVs are tailored for our targeted LRS assay, which covers ~2% of the genome. Users performing whole-genome LRS may prefer to use more stringent criteria (e.g. a lower allele frequency threshold for de-prioritising common variants) to reduce the number of candidate variants for review.

STR genotyping was performed by LongTR^71^ (v1.0), using a set of pre-defined STR sites known to be implicated in genetic myopathies (Supplementary Data 1). All STR sites were also manually inspected in the IGV genome browser to ensure the validity of expansions detected and confirm none were missed. The *PLIN4* exon 5 VNTR was also genotyped using LongTR, and the resulting VCFs were queried (bcftools query -f ‘[%SAMPLE\t%GB\n]’) to extract the GB field, which reports base-pair differences from the reference allele. Samples showing a marked deviation from the hg38 reference were reviewed in IGV to confirm the presence and boundaries of the insertion. To assess the potential protein impact, an alternative MANE Select transcript incorporating the expanded VNTR was generated and translated using ExPASy^72^.

For OPDM-associated STR loci, allele sizes were estimated by running LongTR per-sample on each locus, extracting the ALT sequence from the resulting VCF, and determining repeat copy number using Tandem Repeat Finder (TRF; parameters: 2 7 7 80 10 50 500) in streaming mode (-ngs). Only TRF hits matching the expected repeat motif for each locus were retained. For comparison, allele sizes in 1KGP-LRSC were derived from a pre-existing TRGT-genotyped VCF (1KGP.500.adotto.trgt.vcf.gz). Allele size distributions across both cohorts were visualised as violin plots on a log10 scale, with the shaded region indicating the reported pathogenic expansion range for each locus, as reported in STRchive (https://strchive.org/).

The Pipeface pipeline outputs phased 5mC methylation frequencies, obtained using minimod^69^, for all CpGs within programmed target regions in BED and WIG format. Specific gene promoter regions with known epigenetic disease signatures were then assessed individually for evidence of allele-specific hypermethylation using a custom script and manually visualised in the IGV genome browser, where relevant. A custom analysis method was also used to agnostically flag gene promoters that were methylation outliers in one or more samples, relative to the overall cohort; however, this analysis did not identify any informative differentially methylated genes beyond those already known to be implicated in OPDM.

Mitochondrial variants were called and annotated using the Mitoverse mtDNA-server 2^73^ with a detection limit of 0.1 variant allele fraction (VAF), with heteroplasmy level inferred from the VAF.

### FSHD analysis

We developed a purpose-built, open source software package, d4z4ling (https://github.com/neysa-15/d4z4ling)^74^, to identify and characterise ONT reads selectively enriched with ReadFish^59^ targeting the D4Z4 region on chromosomes 4 and 10, using the T2T-chm13 (v2) reference genome (Supplementary Fig. 13). Reads overlapping the D4Z4 loci were extracted and annotated individually for key features, including the proximal region, short sequence length polymorphism (SSLP), p13-E11 (5′ marker), pLAM (3′ marker), polyadenylation signal (PAS), *Bln*I and *Xap*I restriction enzyme recognition sites and 4qA/4qB-specific probes. Reads were classified as ‘complete’, ‘partial proximal’, ‘partial internal’ or ‘partial distal’ depending on the extent to which they spanned the array. To estimate the number of D4Z4 repeat units, each read was aligned to a single D4Z4 repeat unit and the number of aligned copies was counted. For samples with complete reads and resolved copy numbers for one or both alleles, reads were phased based on D4Z4 repeat count and either polished into a consensus or assembled using Hifiasm^75^. Haplotypes were inferred based on SNVs and indels in the proximal region, SSLP length, sequence of the distal D4Z4 copy and presence of 4qA/4qB-specific features. The *Bln*I and *Xap*I restriction sites were used as supporting evidence to flag reads that may have been misclassified between the 4q and 10q loci, minimising the risk of misclassifying reads. CpG methylation levels were profiled at the distal-most D4Z4 unit with minimod, enabling integration of structural and epigenetic information at single-molecule resolution to inform FSHD diagnosis. Methylation status of each allele was assessed by assessing the median and MAD of CpG methylation of the distal-most D4Z4 unit. Hypomethylation of the distal-most unit of a contracted 4qA D4Z4 array was considered indicative of FSHD1. Hypomethylation of the distal-most D4Z4 unit on both (non-contracted 4q) alleles was considered indicative of FSHD2, as long as one allele had a 4qA haplotype (i.e., either 4qA/4qA or 4qA/4qB). Comparison of methylation within the cohort was not disaggregated for sex or gender because of the small sample size within each diagnostic category (FSHD1, FSHD2, FSHD1 + 2) in this study.

Confident molecular diagnosis of FSHD1 requires complete reads spanning the full D4Z4 repeat array, enabling both precise repeat copy number estimation and methylation profiling. While ONT offers sufficiently long reads for this purpose, the likelihood of capturing a complete read decreases as the number of D4Z4 repeat units increases. To characterise this limitation, we analysed four samples (with alleles carrying 5, 6, 10 and 11 D4Z4 units; Supplementary Fig. 5) in which both 4q alleles could be resolved by either polishing ‘complete’ reads or de novo assembly. We progressively subsampled the read set in these samples to simulate decreasing sequencing depths. At each depth, we performed 50 random subsampling runs and recorded how often at least one complete read was detected. This allowed us to estimate the probability of detecting a complete read as a function of sequencing depth.

D4z4ling was evaluated in this study using adaptive sampling (ReadFish) ONT data only. While there is no technical limitation in the pipeline that would prevent processing of whole-genome ONT or PacBio data, the primary practical limitation for WGS is achieving sufficient coverage at the D4Z4 locus, which is more reliably obtained with target enrichment (either adaptive sampling or Cas9-based enrichment, described elsewhere^52^). In addition, the ~20 kb read length of PacBio sequencing is likely to be insufficient for effective analysis of the D4Z4 region.

### Reporting summary

Further information on research design is available in the Nature Portfolio Reporting Summary linked to this article.

## Supplementary information

Supplementary Information

Description of Additional Supplementary Files

Supplementary Data 1

Supplementary Data 2

Supplementary Data 3

Reporting Summary

Supplementary Source Data

Transparent Peer Review file

## Source data

Source Data

## Data Availability

De-identified sequence data has been deposited in dbGaP under accession phs004623.v1.p1[https://www.ncbi.nlm.nih.gov/projects/gap/cgi-bin/study.cgi?study_id=phs004623.v1.p1]. This sequence data is available under restricted access in order to protect participant privacy and comply with local research governance regulations. Access can be obtained for research related to inherited/genetic myopathic disorders by following established dbGaP Data Access Request processes and providing evidence of local institutional review board approval and a letter of collaboration with primary study investigator(s). The National Institute of Health Data Access Committee generally reviews requests within 2–4 weeks of submission. Once approved, data access is typically granted for one year with a potential for annual renewal. The Vandier et al^47^. data used in this study are available in the National Center for Biotechnology Information Sequence Read Archive under accession PRJNA945898. Source data are provided with this paper.

## Code availability

D4z4ling^74^ and Pipeface are available at Github (https://github.com/neysa-15/d4z4ling and https://github.com/leahkemp/pipeface). Specific code/scripts used to perform the analysis are available on Zenodo 10.5281/zenodo.19601174.
