# Supplementary material for: Targeted long-read sequencing enables comprehensive analysis of the genetic and epigenetic landscape of inherited myopathies
Source: Nat Commun. 2026 Jul 4;17:8324. doi: 10.1038/s41467-026-75144-z (PMC13470197; doi:10.1038/s41467-026-75144-z)
Supplement: Supplementary file 1 [file 41467_2026_75144_MOESM1_ESM.pdf]

Supplementary Information For

Targeted long-read sequencing enables comprehensive analysis of the genetic and epigenetic landscape of inherited myopathies

Dennis Yeow[1-4*], Andre LM Reis[5,6*], Igor Stevanovski[5], Neysa Njo[5], Laura I Rudaks[1-3,7], Bianca R Grosz[2,8], Joanne S Sy[2,9], Leah Kemp[5], Sanjog R Chintalaphani[5], Michael Chin[10], Marion Stoll[10], Danqing Zhu[10], Christina Liang[2,4,11], Katrina A Morris[1,2,7], Andrew Hannaford[1,2], Ehsan Shandiz[12,13], Kate E Ahmad[11], Shadi El-Wahsh[1,2], Stephen W Reddel[1,2], Robert Boland-Freitas[14], Roula Ghaoui[15], Stephanie Barnes[2,16], Jonathan Sturm[17,18], Anna Willard[19], Mahi Jasinarachchi[20], Simon Hawke[2,21], Neil G Simon[22], Lisa Worgan[2,23], David Manser[2,24], Michel Tchan[2,24], Neil C Griffith[25], Ryan L Davis[2,3,26], Michael C Fahey[27], Carolyn M Sue[4,6], Pamela A McCombe[13,28], Karl Ng[2,11], Marina L Kennerson[2,8,10], Pak Leng Cheong[2,10,29], Kishore R Kumar[1-3,6#], Ira W Deveson[5,6#]

1. Neurology Department, Concord Repatriation General Hospital, Sydney, NSW, Australia
2. Faculty of Medicine and Health, University of Sydney, Sydney, NSW, Australia
3. Translational Neurogenomics Group, Garvan Institute of Medical Research, Sydney, NSW, Australia
4. Neurodegenerative Service, Princes of Wales Hospital & Neuroscience Research Australia, Sydney, NSW, Australia
5. Genomic Technologies Lab, Garvan Institute of Medical Research, Sydney, NSW, Australia
6. Faculty of Medicine and Health, University of New South Wales, Sydney, NSW, Australia
7. Clinical Genetics Unit, Royal North Shore Hospital, Sydney, NSW, Australia
8. Northcott Neuroscience Laboratory, ANZAC Research Institute, Sydney Local Health District, Sydney, NSW, Australia
9. Department of Neuropathology, Royal Prince Alfred Hospital, Sydney, NSW, Australia
10. Molecular Medicine Laboratory, Concord Repatriation General Hospital, New South Wales Health Pathology, Sydney, NSW, Australia
11. Department of Neurology, Royal North Shore Hospital, Sydney, NSW, Australia
12. Department of Neurology, Toowoomba Base Hospital, Toowoomba, QLD, Australia
13. Centre for Clinical Research, University of Queensland, Brisbane, QLD, Australia
14. Department of Neurology, Blacktown Hospital, Blacktown, NSW, Australia
15. Department of Neurology, Central Adelaide Local Health Network, Royal Adelaide Hospital, Adelaide, SA, Australia
16. Department of Neurology, Hornsby Ku-ring-gai Hospital, Sydney, NSW, Australia
17. Central Coast Neurosciences Research, Tumbi Umbi, NSW, Australia
18. The University of Newcastle, Callaghan, NSW, Australia
19. Department of Neuroscience, School of Translational Medicine, Alfred Health and Monash University, Melbourne, VIC, Australia
20. Department of Neurology and Neurological Research, St. Vincent's Hospital Melbourne, Melbourne, VIC, Australia
21. Central West Neurology and Neurosurgery, Orange, NSW, Australia
22. Faculty of Medicine and Health Sciences, Macquarie University, Sydney, NSW, Australia
23. Clinical Genetics Service, Royal Prince Alfred Hospital, Camperdown, New South Wales, Australia
24. Department of Genetic Medicine, Westmead Hospital, Sydney, NSW, Australia
25. Department of Neurology, Liverpool Hospital, Sydney, NSW, Australia
26. Neurogenetics Research Group, Kolling Institute, School of Medical Sciences, Faculty of Medicine and Health, University of Sydney and Northern Sydney Local Health District, Sydney, New South Wales, Australia
27. Department of Paediatrics, Monash University, Melbourne, VIC, Australia
28. Neurology Department, Royal Brisbane & Women's Hospital, Brisbane, QLD, Australia
29. Institute of Precision Medicine and Bioinformatics, Sydney Local Health District, NSW, Australia

* These authors contributed equally
# These authors jointly supervised this work; corresponding authors

Correspondence to:
Kishore R Kumar: k.kumar@garvan.org.au
Ira W Deveson: i.deveson@garvan.org.au

Table of Contents

Supplementary Note 1 ... 3

Supplementary Note 2 ... 4

Supplementary Table 1. ... 5

Supplementary Figure 1. ... 6

Supplementary Figure 2 ... 7

Supplementary Figure 3 ... 9

Supplementary Figure 4 ... 10

Supplementary Figure 5 ... 11

Supplementary Figure 6 ... 12

Supplementary Figure 7 ... 13

Supplementary Figure 8 ... 14

Supplementary Figure 9 ... 16

Supplementary Figure 10 ... 17

Supplementary Figure 11 ... 18

Supplementary Figure 12 ... 19

Supplementary Figure 13 ... 20

Supplementary Methods ... 21

Supplementary References ... 22

Supplementary Note 1

In case L140, prior NGS identified one pathogenic nonsense variant (NM_000687.4:c.346C>T) and one missense VUS (NM_000687.4:c.1213A>G) in *AHCY*, but phasing of variants was uncertain in this participant with a LGMD phenotype. ONT LRS identified these variants were in *trans*, allowing for American College of Medical Genetics and Genomics (ACMG) PM3 criteria to be applied to the missense VUS. However, this was insufficient to upgrade the VUS to likely pathogenic/pathogenic (LP/P). Biallelic variants in *AHCY* typically cause an early-onset, complex, multisystem syndrome involving muscle, liver and the nervous system, although milder, adult-onset presentations, which can include myopathy, have been reported more recently.[1] Patients with *AHCY*-related disorders may have globally increased DNA methylation and/or hypermethylation of specific imprinted genes.[1] Targeted ONT LRS did not identify abnormal methylation across the 331 myopathy loci, nor did subsequent clinical EpiSign testing identify the typical methylation profile of *AHCY*-related disorders. However, it is possible that the more attenuated phenotype seen in adult-onset *AHCY*-related disorders may also correlate to attenuation of the abnormal methylation profile. Assessment of serum S-adenosyl-l-homocysteine and S-adenosyl-l-methionine, which are typically elevated in *AHCY*-related disorders,[2] could be considered to further evaluate the *AHCY* VUS.

In case L146 (Family 29 previously reported by Ghaoui et al.[3]), a patient with a LGMD phenotype, prior NGS identified a missense VUS (NM_213599.3:c.240T>G) and an extended splice acceptor site VUS (NM_213599.3:c.1120-3C>T) in *ANO5*, but phasing was uncertain as family samples were not available. SpliceAI predicted the extended splice acceptor VUS likely results in loss of the acceptor site (Δ0.52) as well as loss of a downstream splice donor site (Δ0.69). Additionally, a set of previously derived data-driven heuristics for interpreting potentially splice-altering variants suggests that there is a 70.4% likelihood of this variant disrupting splicing.[4] ONT LRS identified these variants were in *trans*. Although this was not sufficient to upgrade either VUS to LP/P, the biallelic status of the variants provides impetus to proceed with functional studies of these VUS. *ANO5* is not well expressed in skin so patient-derived fibroblast splicing studies were not performed to evaluate the extended splice acceptor VUS. To this end, splicing studies using patient-derived muscle tissue or development of a custom minigene assay are under consideration.

Supplementary Note 2

Expansions of the normal 29–31 × 99 bp VNTR in exon 5 of *PLIN4* to 37–50 × 99 bp have been described to cause a vacuolar myopathy, often with prominent distal weakness, with onset in the 30–50s.[5-7] Larger repeats are thought to cause an earlier onset and more severe phenotype. The expanded *PLIN4* VNTR sequence seen in case L286 and her asymptomatic father (L144) is 39 × 99 bp in length, which would fall within the previously described pathogenic range (37–50 units reported in STRchive; https://strchive.org/).[8]

Although both *NOTCH2NLC*-related ODPM3 and *PLIN4*-associated myopathy can cause a distal pattern of muscle weakness, OPDM3 is the favoured diagnosis in case L286 given the phenotype of oculopharyngodistal weakness; in particular, ptosis and ophthalmoplegia have not been described in *PLIN4* myopathy to date. The presence of the *PLIN4* VNTR expansion in the father, aged 70, who does not have any myopathic features, may also suggest that this particular 39 × 99 bp VNTR allele may not be pathogenic, or at the very least not fully penetrant. A possible explanation comes from a recent study that identified that, because the individual 99 bp (33 amino acid) repeats units within the *PLIN4* VNTR are not identical, separate to overall VNTR expansion length, the exact amino acid composition produced by specific added 99 bp repeats may modulate the phenotype of *PLIN4*-associated myopathy.[9] In particular, a duplication of a specific contiguous group of 14 × 99 bp repeat units spanning amino acids 491–953 (yielding a 45 × 99 bp VNTR sequence) was found in a patient with later onset and a much milder phenotype compared to cases with the earlier described expansion of a single repeat unit spanning amino acids 326–359 (yielding 40–50 × 99 bp VNTR sequences). The "added" repeat units identified in cases L286 and L144 were neither of these previously described arrangements (Supplementary Fig. 9), and so pathogenicity (and/or penetrance) of the inserted repeats in this case is unclear.

We also evaluated *PLIN4* VNTR alleles among 500 individuals with public LRS data currently available via the 1000 Genomes Project Long-read Sequencing Consortium (1KGP-LRSC). Using a TRGT VCF file and LRS alignments obtained from 1KGP-LRSC, we identified and confirmed by manual inspection three individuals (HG0335, HG02884, HG03072) with alleles expanded to ≥ 35 × 99 bp copies, including one with 38 copies (one copy shorter than L286 and L144). Given all individuals in this cohort are not known to be affected by disease, this provides further evidence of incomplete penetrance for *PLIN4* expansions in the lower end of the pathogenic range. Characterisation of diverse *PLIN4* VNTR expansions/duplications/insertions in larger cohorts will be required to further refine the genotype-phenotype relationship for *PLIN4* myopathy.

Supplementary Table 1. Comparison of GIPC1 SNPs in newly diagnosed OPDM2 patients compared to a known Japanese OPDM2 haplotype

Japanese OPDM2 haplotype SNPs[10] (hg38 coordinates)	**L126** (European Australian)		**L136** (Chinese)		**L248** (New Zealand Māori)		**L437** (New Zealand Māori, European Australian, Tongan)	
	STR expanded allele	Other allele	STR expanded allele	Other allele	STR expanded allele	Other allele	STR expanded allele	Other allele
chr19:14493342 T>A	A	A	A	T	A	A	A	A
chr19:14494246 T>C	C	C	C	C	C	C	C	C
chr19:14494369 G>A	A	G	A	G	A	G	A	G
chr19:14494383 C>T	T	C	T	C	T	T	T	T
chr19:14497761 G>A	G*	G	G*	G	A	G	A	G
chr19:14498125 G>A	A	A	A	G	A	A	A	A
chr19:14498399 A>G	G	G	G	A	G	G	G	G

OPDM2; oculopharyngodistal myopathy type 2; SNPs, single nucleotide polymorphisms
*mismatches with the Japanese OPDM2 haplotype

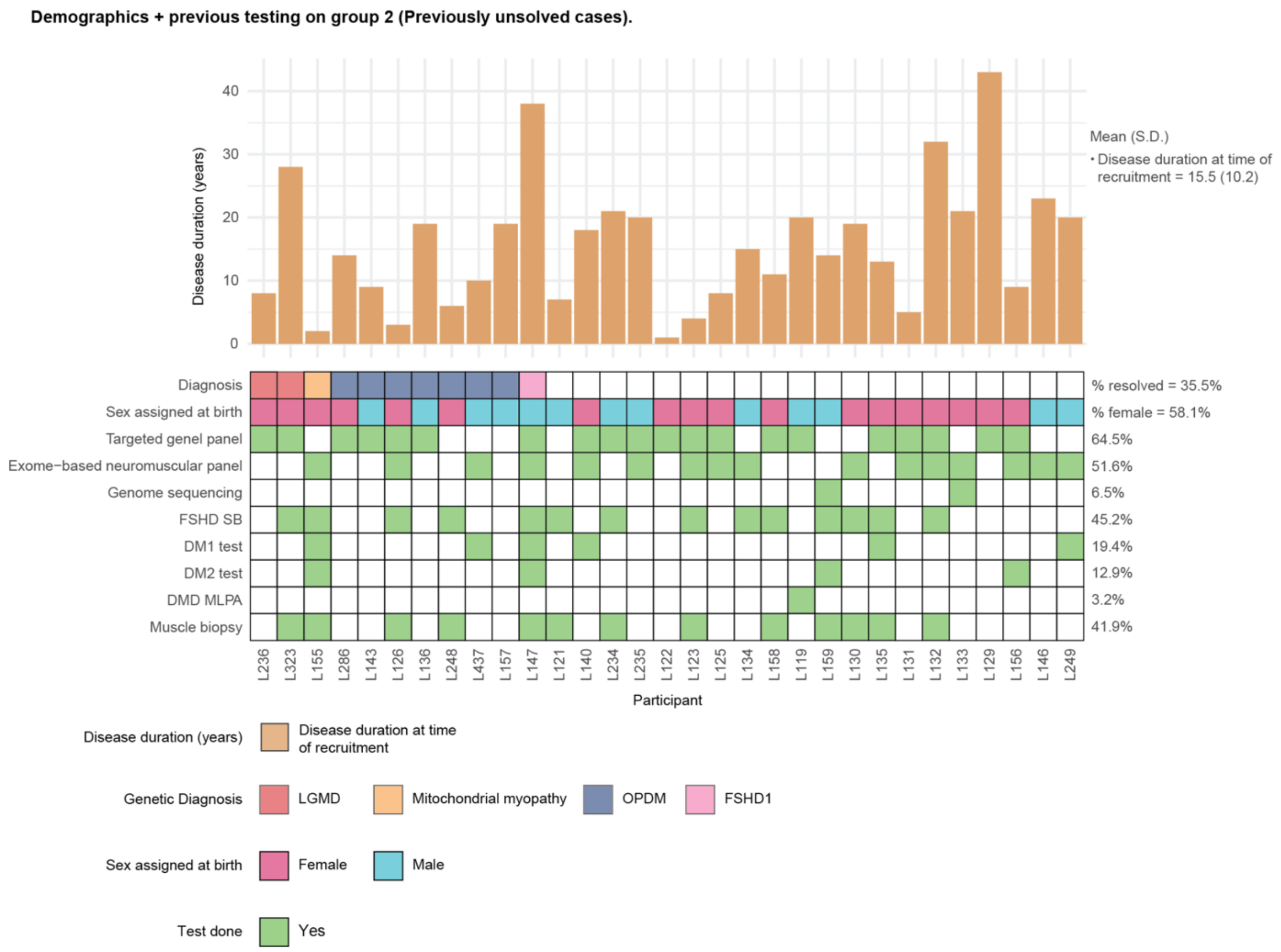

Supplementary Figure 1. Demographics and characteristics of participants with genetically unsolved or incompletely solved myopathies prior to Oxford Nanopore Technologies long-read sequencing (ONT LRS) The upper bar chart shows disease duration for each participant. Mean and standard deviations are reported next to the plot. Coloured boxes in the first row of the matrix indicate the diagnosis for each participant following ONT LRS. Green boxes in the remaining rows indicate previous testing performed in each participant. Source data are provided in a Source Data file.

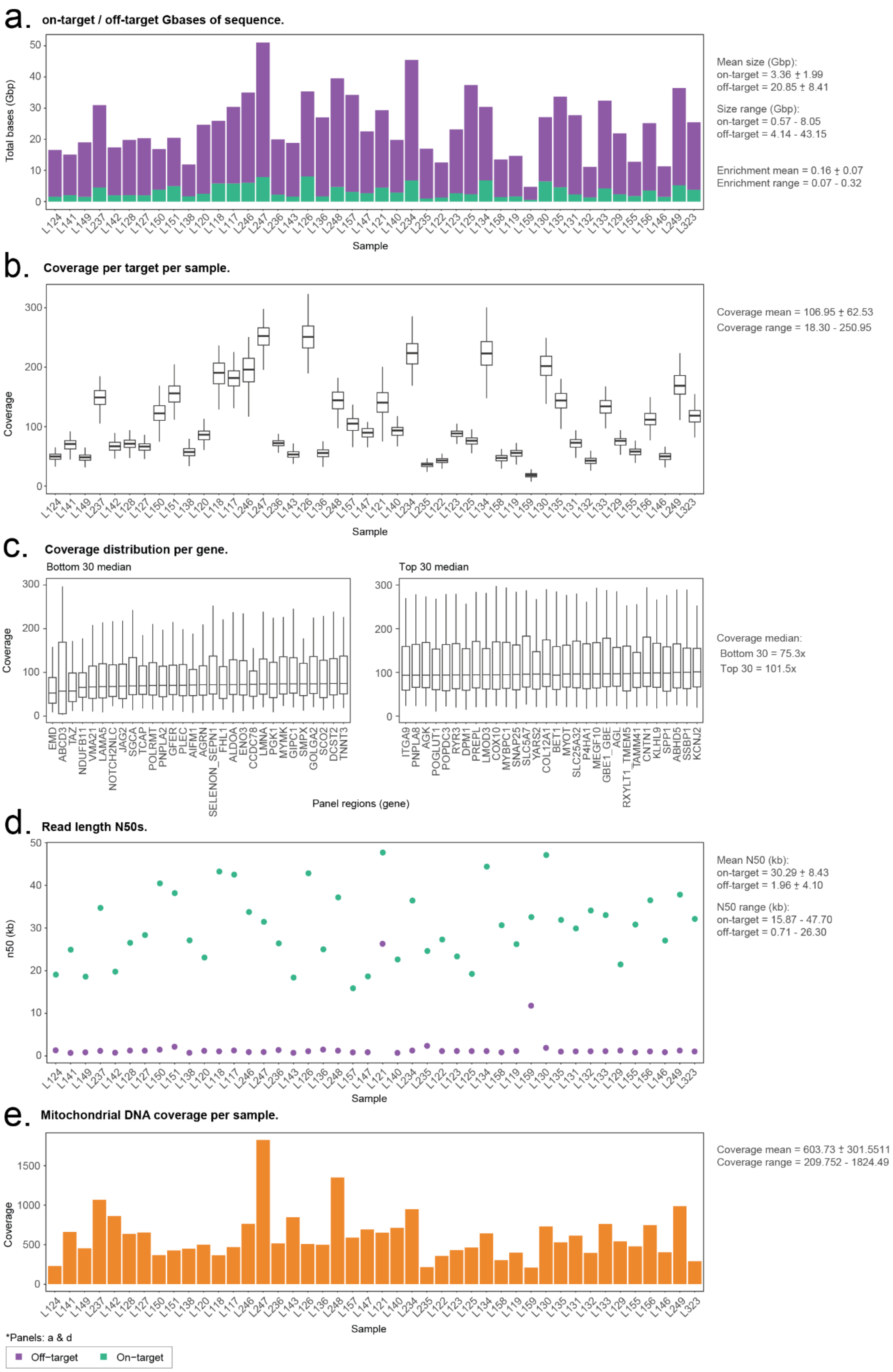

Supplementary Figure 2. Targeted Oxford Nanopore Technologies long-read sequencing (ONT LRS) myopathy panel sequencing metrics.

a Myopathy cohort sequencing yield showing relative proportions of data aligned to the genome within ('on-target'; green) and outside ('off-target'; purple) of programmed target regions. **b** Distribution of sequencing coverage across the cohort. Boxplots summarise the per-target coverage distribution for each individual. Note: variations in read depth between samples were primarily driven by the level of multiplexing used, with some samples sequenced individually and others pooled up to three samples per flow cell (see Methods). There are 328 genes contributing to each boxplot. **c** Boxplots summarise the per-individual coverage distribution for each gene within the target panel. Due to space constraints, only the top 30 and bottom 30 genes (based on median coverage depth) are shown. There are 49 samples contributing to each boxplot. **d** On-target (green) and off-target (purple) read length N50s distribution across the cohort. The presence of off-target reads among the sequencing data reflects the fact that the first ~100-500bp of a given DNA fragment is sequenced before a decision can be made to 'reject' a read originating outside the target region. While these off-target reads are shorter than on-target reads (due to their rejection), they account for the majority of reads produced during the experiment. Note: samples L121 and L159 were affected by a temporary issue that caused targeted sequencing with ReadFish to stall for several hours during the experiment. During this time sequencing proceeded without executing the rejection of off-target reads. The issue was identified and rectified during the experiment but the resulting libraries have a portion of off-target reads larger than that obtained for other samples. **(e)** Mitochondrial DNA coverage obtained for each individual. For (**b**, **c**), boxplots show the median (centre line), first and third quartiles (hinges), and most extreme values within 1.5 × the interquartile range of the hinges (whiskers). Source data are provided in a Source Data file.

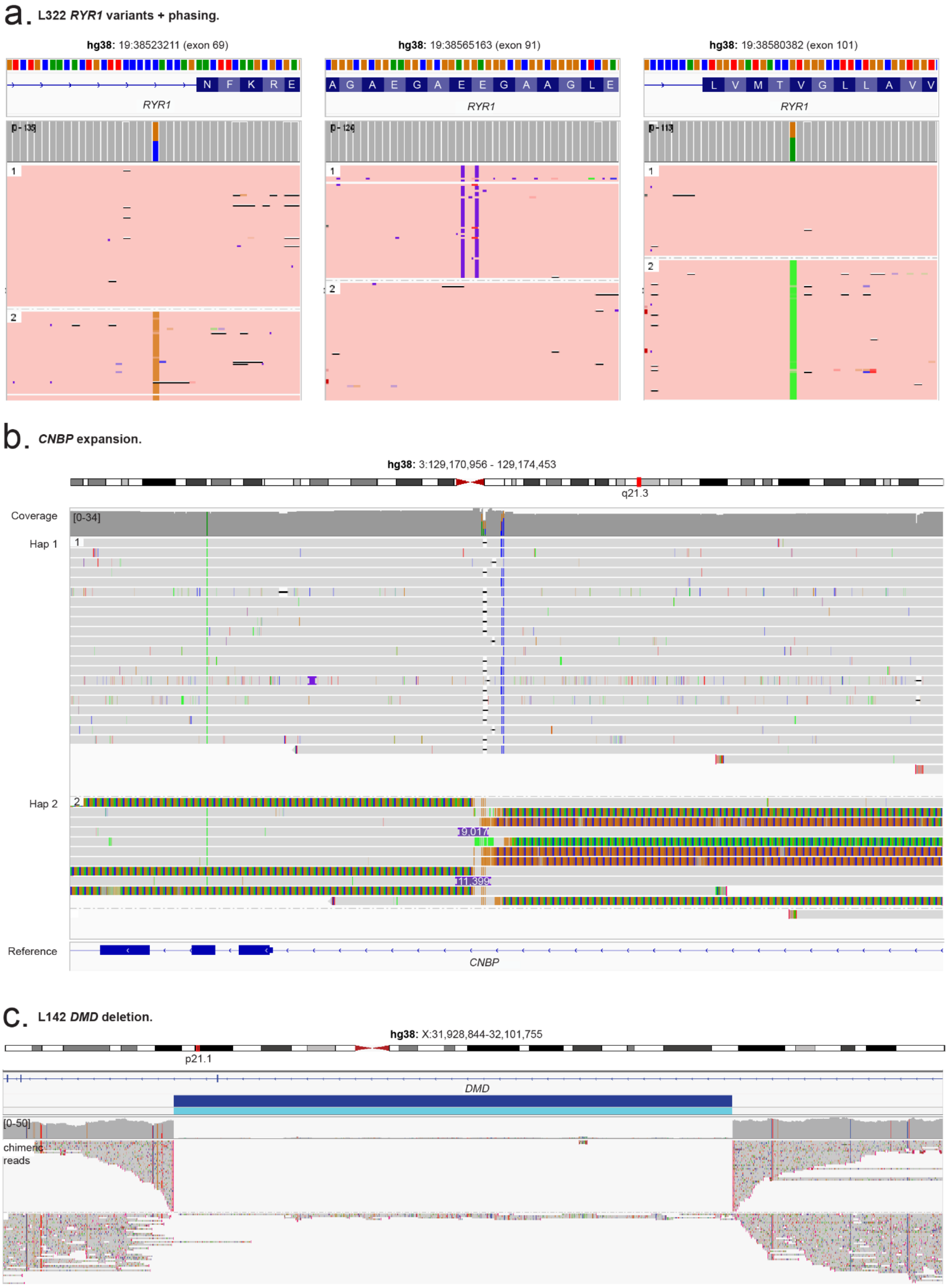

Supplementary Figure 3. Additional positive control genetic variants identified by Oxford Nanopore Technologies long-read sequencing (ONT LRS).

a Case L141 (known diagnosis of congenital myopathy 1B) harbours three known variants in *RYR1*. ONT LRS identified that two variants (c.10348-6C>G and c.14524G>A) were on one allele while the third (c.12829G>T) was on the other allele. Genome browser view shows alignments grouped by haplotype and coloured by 'PS tag' (note: colouring is uniform because all alignments are contained in a single phase block/set). **b** Case L237 (known diagnosis of myotonic dystrophy type 2) harbours a CCTG short tandem repeat expansion in *CNBP* with ONT LRS identifying repeat length mosaicism (2275–2870 repeats). **c** Case L142 (known diagnosis of Duchenne muscular dystrophy) harbours a deletion of *DMD* exon 45. ONT LRS generated chimeric reads spanning the deletion and identified the exact breakpoints of the deletion: c.6439-94713_6614+8075del.

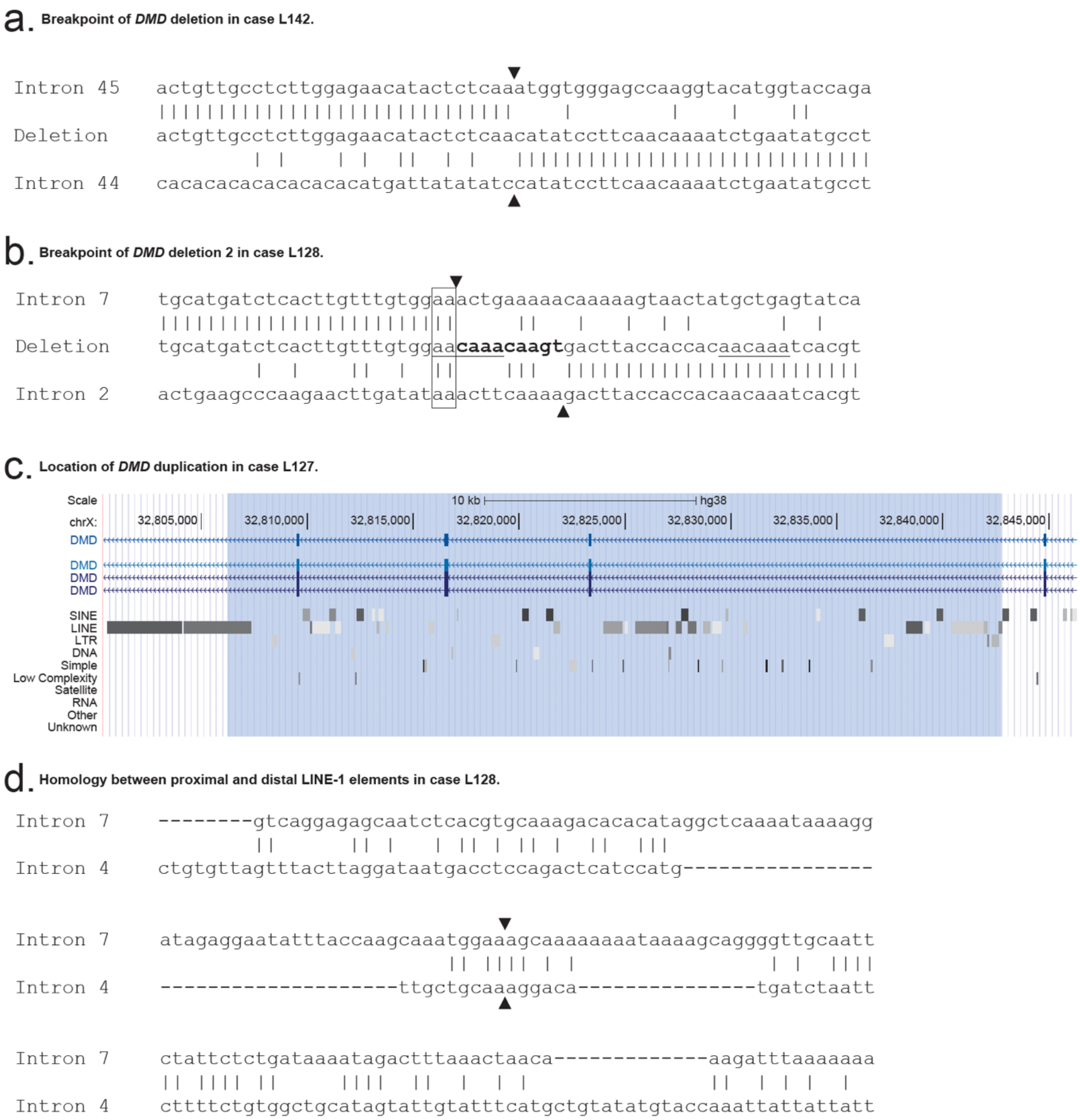

Supplementary Figure 4. **Oxford Nanopore Technologies long-read sequencing (ONT LRS) characterises exact *DMD* copy number variation breakpoint junctions, informing potential mechanism of copy number variant generation.**

a The deletion of exon 45 of *DMD* in case L142 was identified to have a clean breakend (arrowheads), consistent with non-homologous end-joining as a mechanism for the deletion.[11] **b** The deletion of exons 3–7 of *DMD* in case L128 was identified to have a novel 9 bp insertion (bold) at the deletion junction (arrowheads), part of which may be a copy of a nearby 4 bp tract (underlined). One of the deletion breakpoints is also adjacent to a 2 bp region of microhomology (boxed), perhaps indicating microhomology-mediated break induced replication as a possible mechanism.[11] **c** The two ends of the *DMD* exon 5–7 duplication in case L127 were found to be located within LINE-1 segments (highlighted in blue in the UCSC Genome Browser screenshot) indicating this duplication may have resulted from LINE-LINE-mediated nonallelic homologous recombination.[12] **d** The proximal and distal LINE-1 sequences in Case L127 demonstrate homology surrounding the breakpoint (arrowheads).

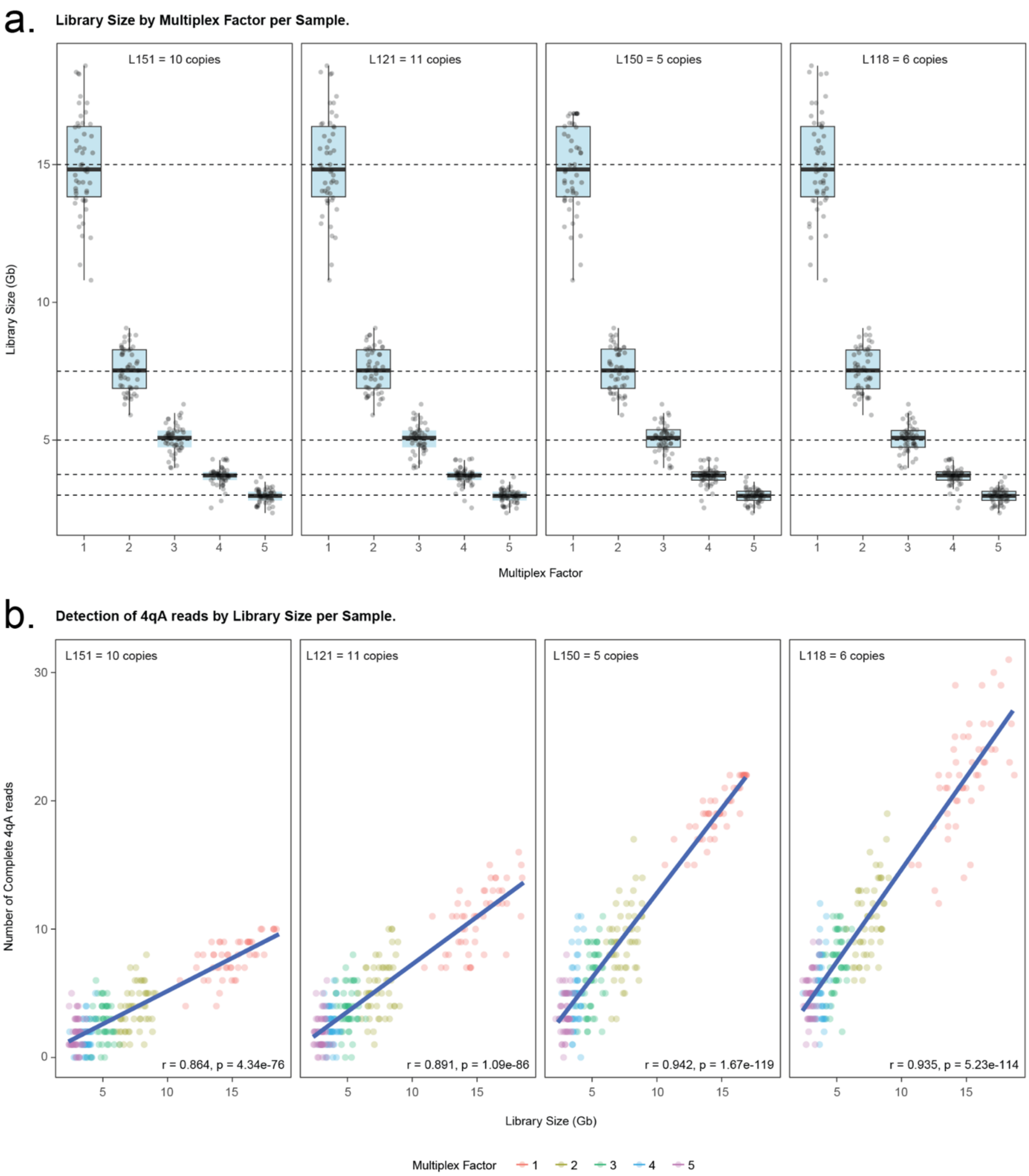

Supplementary Figure 5. **Fascioscapulohumeral muscular dystrophy downsampling experiments.**
a Limit of detection analysis for library size. Each panel shows the library size distribution attained when simulating increasing multiplexing (sequencing the sample together with different numbers of samples) in representative individuals with varying 4qA D4Z4 copy numbers (5, 6, 10 & 11). The boxplots show the median (centre line), first and third quartiles (hinges), and the most extreme values within 1.5 × the interquartile range of the hinges (whiskers). The number of reads contributing to each boxplot varied between samples (50 to 70). **b** Limit of detection analysis for D4Z4 copy number. Each panel shows the number of complete 4qA reads when the library size increases, based on n = 50 independent subsampling repetitions per multiplex factor. Pearson correlation coefficients were calculated to assess the linear association between variables. Corresponding p-values were derived using a two-sided test of no correlation. Source data are provided in a Source Data file.

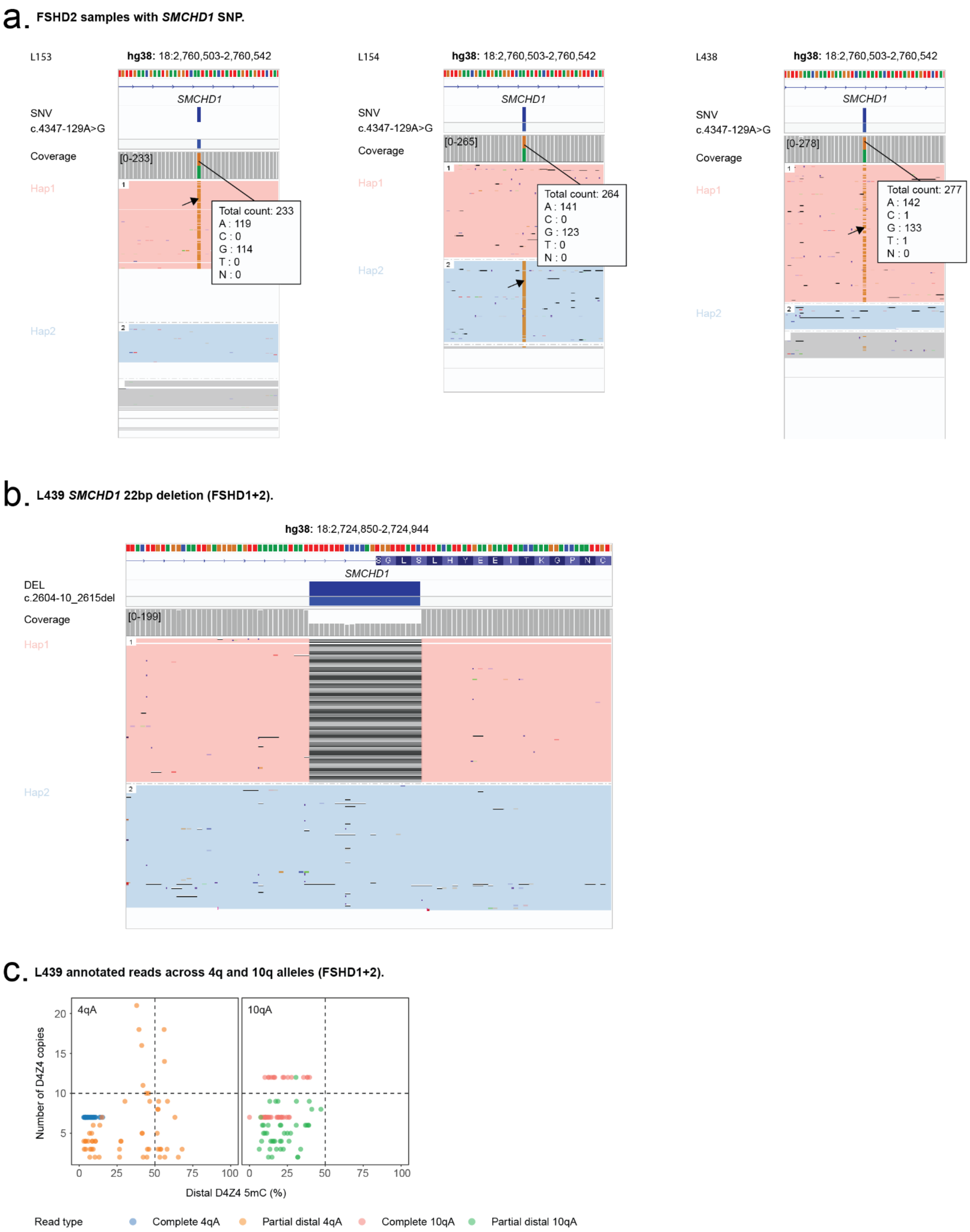

Supplementary Figure 6. *SMCHD1* variants in fascioscapulohumeral muscular dystrophy (FSHD) type 2 and type 1+2 cases and D4Z4 copy number and methylation analysis in FSHD1+2 case.
a In cases L438, L153 and L154 (known diagnoses of FSHD2), Oxford Nanopore Technologies long-read sequencing (ONT LRS) identified the known deep intronic *SMCHD1* variant (c.4347-129A>G) which had previously been shown to be pathogenic by an external research laboratory. **b** In case L439 (known diagnosis of FSHD1+2), ONT LRS identified the known likely pathogenic deletion in *SMCHD1* (c.2604-10_2615del). **c** Per-read 4qA and 10qA D4Z4 copy number and distal-most D4Z4 unit methylation fraction plot for case L439. Dashed lines represent an arbitrary 50% methylation value (vertical) and the 10-copy diagnostic threshold for FSHD1 (horizontal). Source data for (**c**) are provided in a Source Data file.

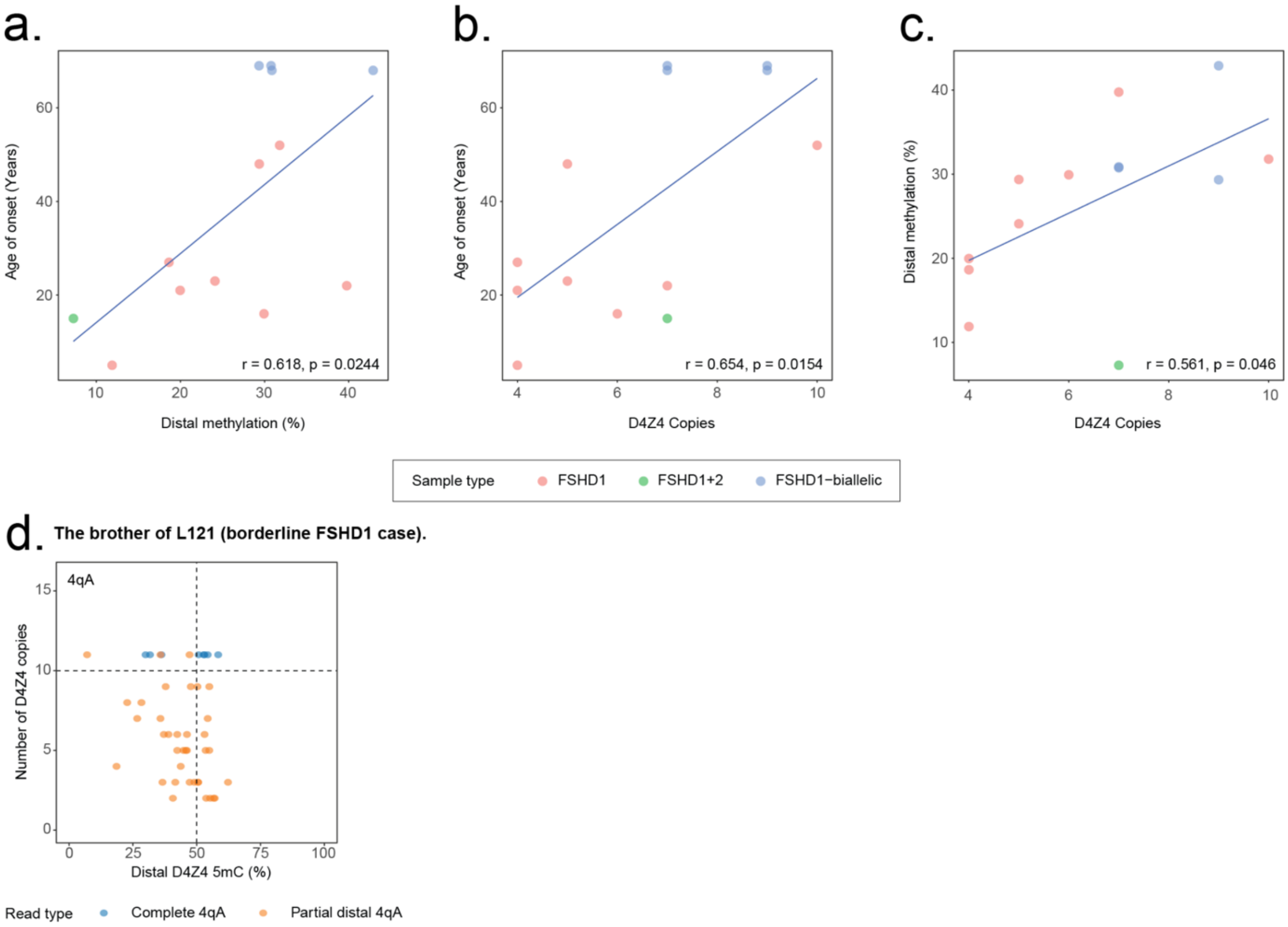

Supplementary Figure 7. **Correlation between age of onset, D4Z4 copy number and D4Z4 methylation fraction in fascioscapulohumeral muscular dystrophy (FSHD) type 1 and 1+2 patients and brother of 11-copy D4Z4 array case**

a Scatter plot of age of onset and mean distal-most 4qA D4Z4 unit methylation fraction. **b** Scatter plot of age of onset and 4qA D4Z4 copy number. **c** Scatter plot of mean distal-most 4qA D4Z4 unit methylation fraction and 4qA D4Z4 copy number. Pearson correlation coefficients were calculated to assess the linear association between variables. Corresponding p-values were derived using a two-sided test of no correlation. **d** Per-read 4qA D4Z4 copy number and distal-most D4Z4 unit methylation fraction plot for the brother of case L121. Dashed lines represent an arbitrary 50% methylation value (vertical) and the 10-copy diagnostic threshold for FSHD1 (horizontal). Source data are provided in a Source Data file.

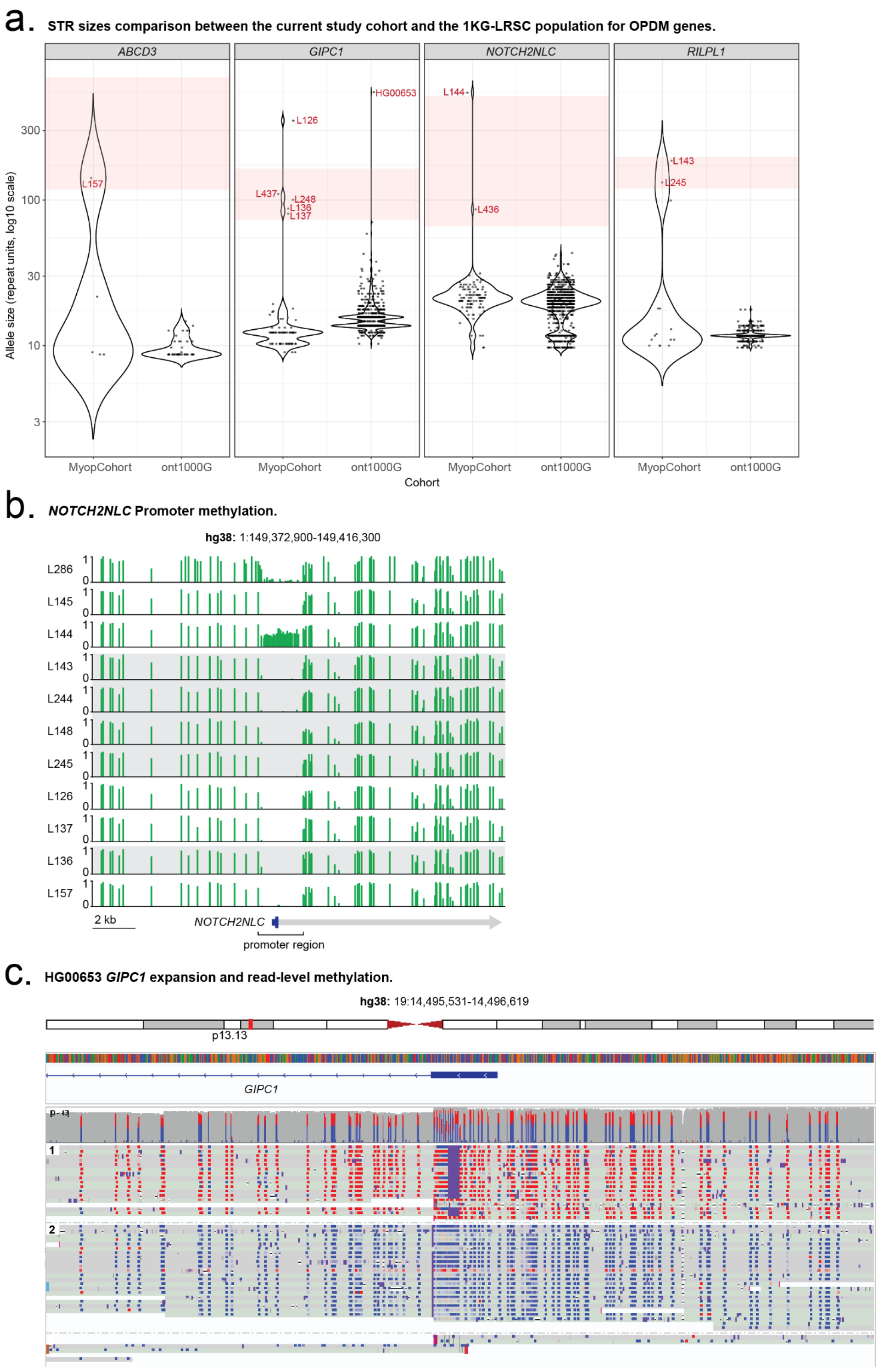

Supplementary Figure 8. **Oculopharyngodistal myopathy (OPDM) short tandem repeat (STR) expansion size and methylation assessment.**

a Dot plot of STR allele sizes for OPDM genes observed among LRS population reference data from the 1KGP-LRSC cohort (*n* = 500 individuals plotted; ont1000G) in comparison to individuals from the current myopathy cohort (MyopCohort). Pink shaded regions demarcate the reported pathogenic size ranges for each gene from STRchive.[8] **b** *NOTCH2NLC* methylation chart for OPDM participants in the current myopathy cohort. Each row corresponds to an individual, with vertical bars showing methylation levels at different CpG sites. Case L144, who harbours a $(GGC)_{273\text{–}905}$ STR expansion in *NOTCH2NLC* but is asymptomatic, demonstrates

hypermethylation of the *NOTCH2NLC* STR expansion site and promoter region. In comparison his daughter (L286), who harbours a $(GGC)_{130–621}$ STR expansion in *NOTCH2NLC* and has a diagnosis of OPDM2, as well as all other OPDM patients and their family members demonstrate little methylation in this region. **c** Genome browser view of *GIPC1* promoter region read-level methylation data in 1KGP-LRSC individual HG00653. CpG sites are coloured by methylation state (red = methylated, blue = unmethylated). Allele-specific hypermethylation is evident across the large expanded allele and the *GIPC1* promoter region for HG00653, who is assumed to be non-symptomatic. Source data for (**a**) are provided in a Source Data file.

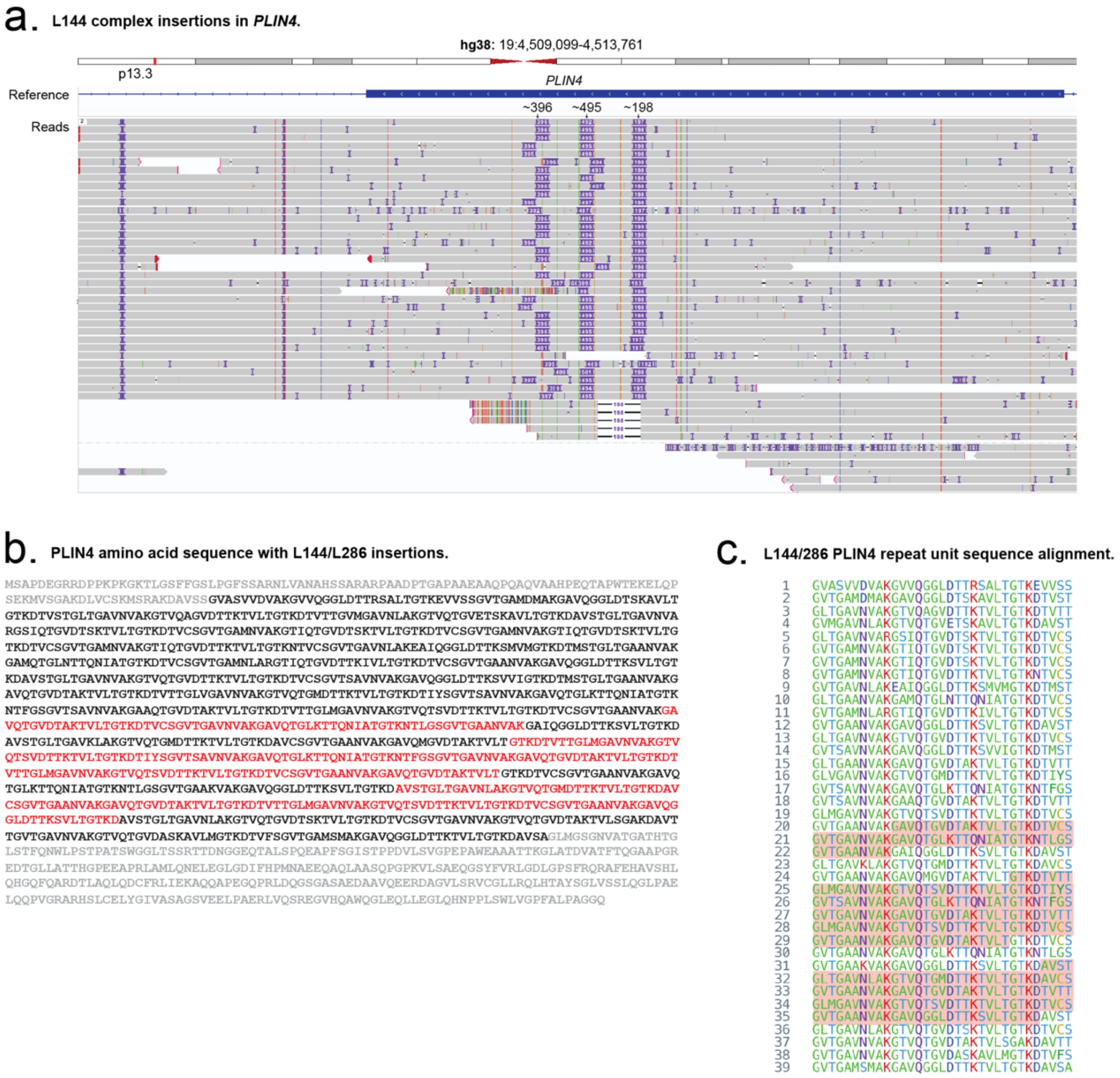

Supplementary Figure 9. **Complex *n* × 99 bp repeat insertions in the *PLIN4* exon 5 variable number tandem repeat (VNTR) in cases L286 and L144.**

a IGV browser view of the *PLIN4* exon 5 VNTR showing that, relative to the hg38 reference (which contains approximately 28 repeat units, about two fewer than most individuals), case L144 (and his daughter, case L286) carries 11 additional 99 bp repeat units, visible as three large insertions (4 × 99 bp = 396 bp, 5 × 99 bp = 495 bp and 2 × 99 bp = 198 bp). **b** Amino acid sequence of the expanded PLIN4 allele in cases L144 and L286 generated by adding the three large insertions (396 bp, 495 bp and 198 bp) into their precise positions in exon 5 of the *PLIN4* MANE Select coding sequence and scanning for the correct open reading frame. Amino acids encoded by the full 39 × 99 bp VNTR are shown in bold, and those encoded by the inserted repeat units are highlighted in red. **c** Alignment of the 39 × 33 aa repeat units encoded by the expanded VNTR in cases L144 and L286. Red shading denotes amino acids derived from the inserted repeat units.

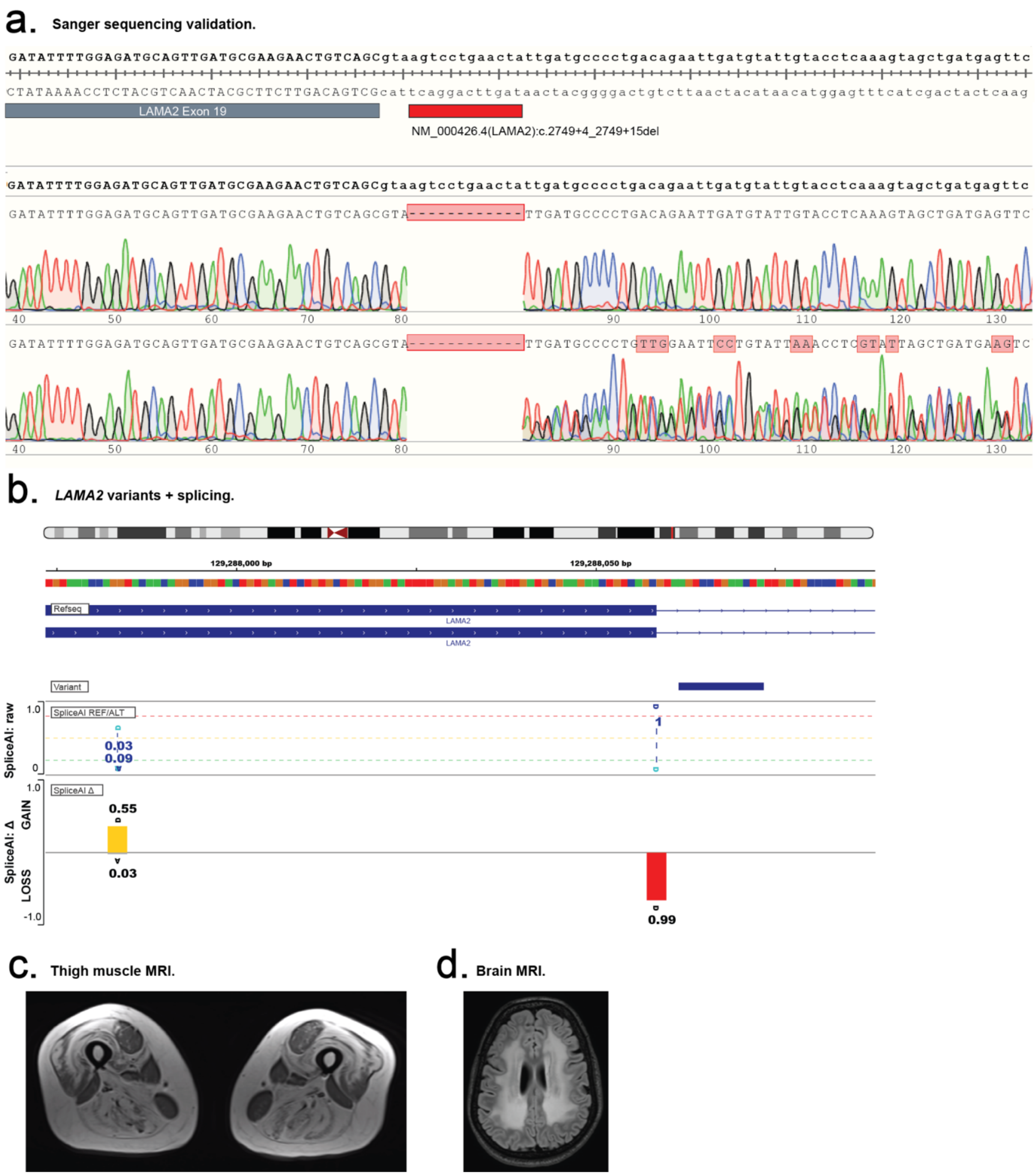

Supplementary Figure 10. New diagnosis of *LAMA2*-related limb-girdle muscular dystrophy (LGMD) R23.

a Sanger sequencing validation of the *LAMA2* c.2740+4_2749+15del variant in L236 (top track; homozygous) and her asymptomatic mother (bottom track; heterozygous carrier - indicated by apparent frameshift following the deletion). **b** SpliceAI predictions for *LAMA2*:c.2749+4_2749+15del indicate likely loss of the canonical splice donor site (Δ0.99) and use of a cryptic donor splice site (Δ0.55) that is 78 bp upstream, within exon 19. **c** Thigh MRI T1 sequence in L236 showing a pattern of muscle involvement consistent with LGMD R23, with severe fatty infiltration of multiple muscle compartments but relative sparing of the rectus femoris, gracilis, sartorius, and short head of the biceps femoris. **d** Brain MRI FLAIR sequence in L236 showing confluent white-matter hyperintensity, characteristic of LGMD R23.

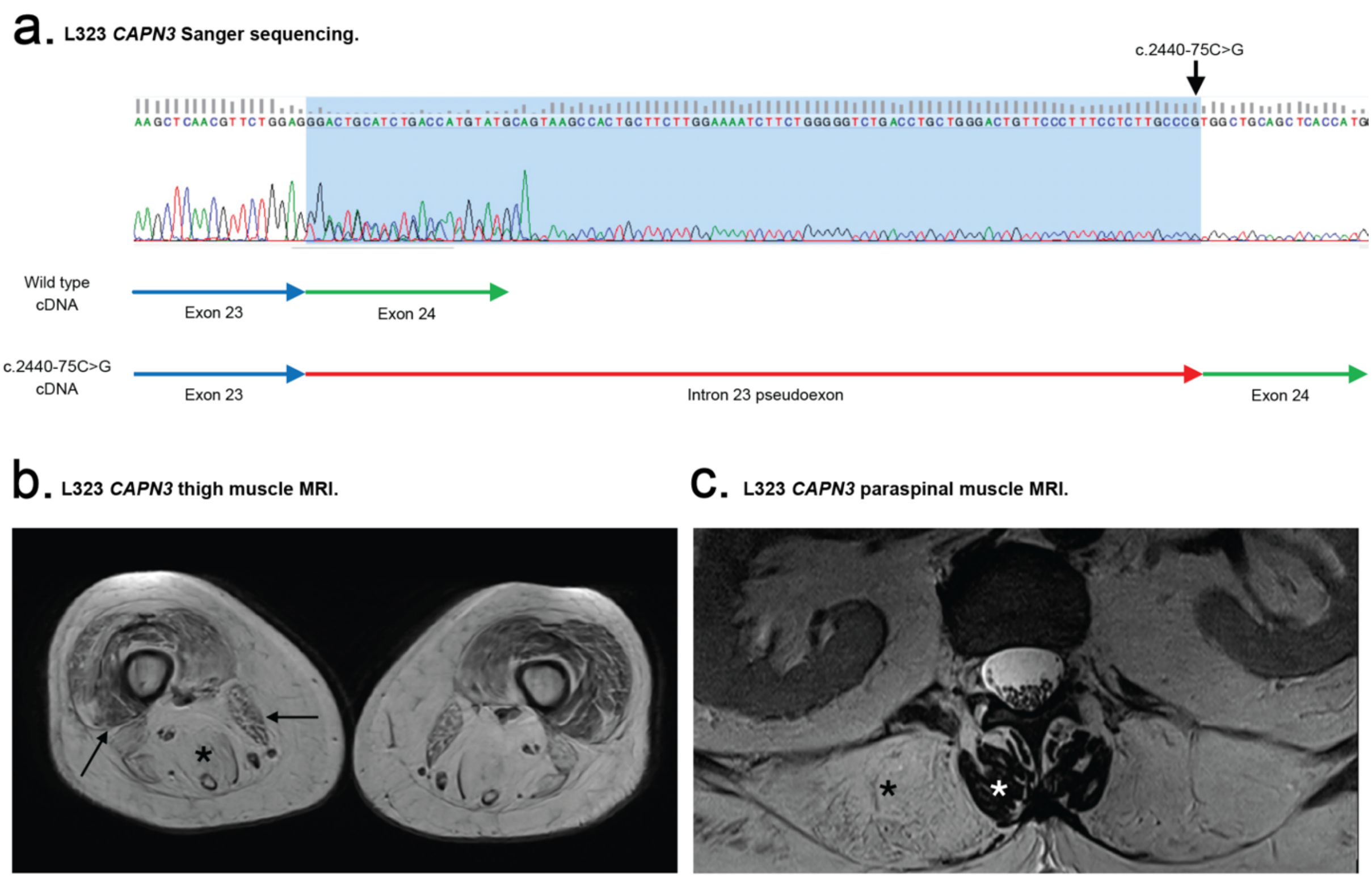

Supplementary Figure 11. New diagnosis of *CAPN3*-related limb-girdle muscular dystrophy (LGMD) R1. **a** Sanger sequencing chromatogram of reverse transcription polymerase chain reaction product from L323 focused on exon 23, intron 23 and exon 24 showed two products: 1) the native exon splice product (exon 23-exon 24 junction); and 2) a novel splice product wherein the *CAPN3* c.2440-75C>G variant resulted in incorporation of a 95 bp pseudoexon sequence from intron 23 into the transcript between exons 23 and 24: NC_000015.10 (NM_000070.3):r.2439_2440ins[2440-169_2440-76;G]. **b** Thigh MRI T1 sequence showing a pattern of muscle involvement consistent with LGMD R1 including severe fatty infiltration of the posterior and medial compartments and less severe fatty infiltration of anterior muscle groups and also the gracilis (arrows). **c** Lower thoracic spine MRI T2 sequence showing severe fatty infiltration of thoracic erector spinae muscles (black asterisk) but sparing of the immediately adjacent thoracic transversospinalis muscles (white asterisk), consistent with LGMD R1.

L155 whole blood mtDNA deletions.

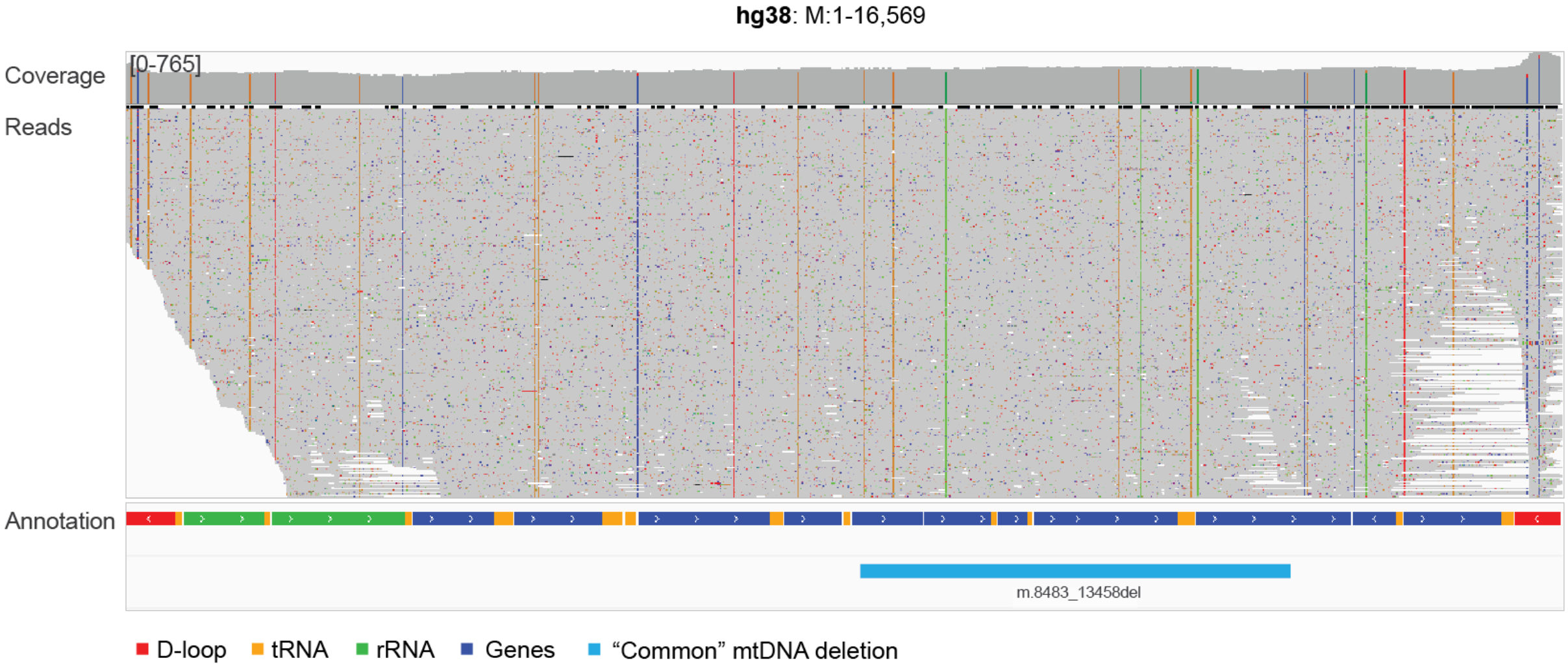

Supplementary Figure 12. Oxford Nanopore Technologies long-read sequencing mitochondrial DNA reads from blood in participant L155.

ONT LRS reads mapping to the mitochondrial genome revealed no large mtDNA deletions in blood-derived DNA from L155, in contrast to the multiple deletions that were identified in muscle-derived DNA from the same participant.

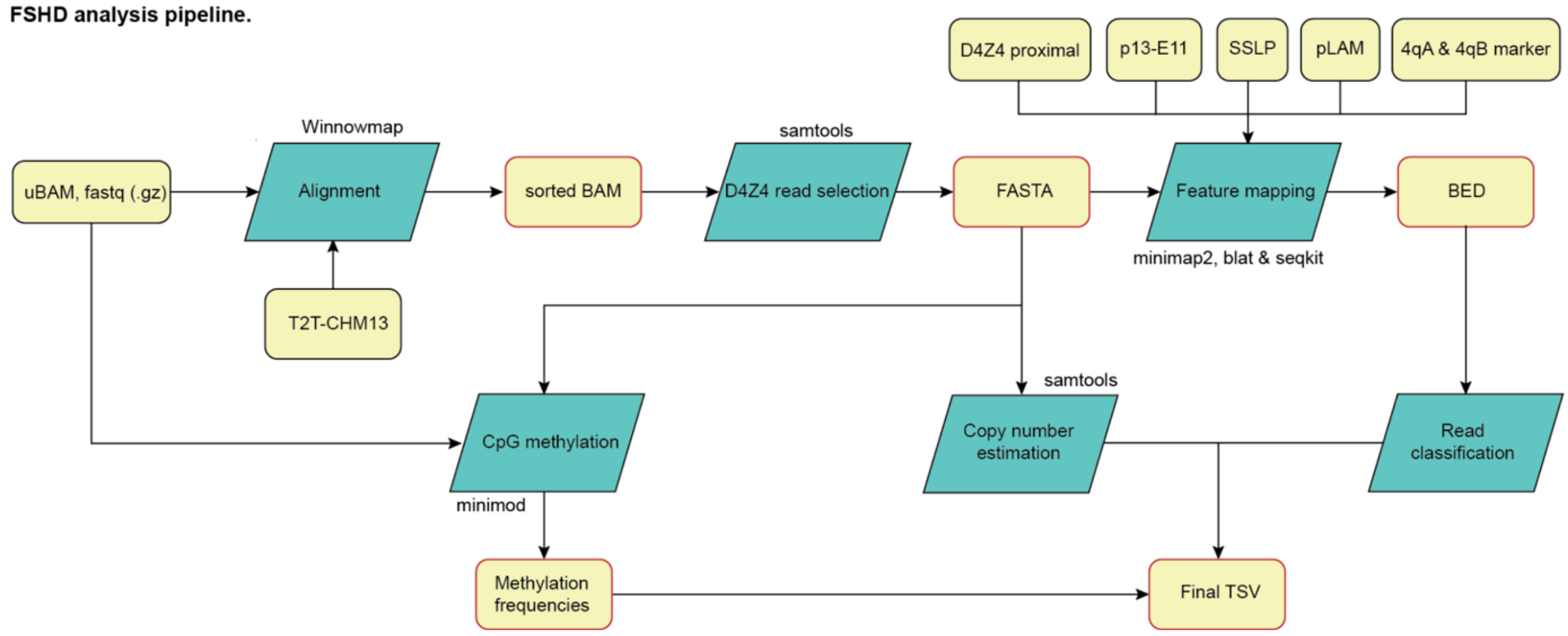

Supplementary Figure 13. **Targeted Oxford Nanopore Technologies long-read sequencing (ONT LRS) facioscapulohumeral muscular dystrophy (FSHD) analysis pipeline.**

Flowchart of the newly developed FSHD analysis pipeline that uses LRS data to resolve the genetic and epigenetic architecture of FSHD. Inputs/outputs are represented by a yellow rounded rectangle, and processes represented by a green parallelogram. The process begins with aligning uBAM against the T2T-chm13 (v2) reference genome using Winnowmap, resulting in a sorted BAM file. The D4Z4 regions of chr4 and chr10 are then selected from the file and converted to a FASTA format. Each read is annotated with key features, including proximal elements (SSLP, p13-E11), the distal pLAM region, D4Z4 repeat units, and 4qA/4qB haplotype-specific marker using minimap2, blat and samtools, allowing the copy number estimation and classification of individual reads. Additionally, the FASTA file is mapped back to the uBAM for CpG methylation analysis with minimod, yielding methylation frequencies. Finally, all the genetic and epigenetic information are integrated into one final TSV output.

Supplementary Methods

All PCR primers used in this study were synthesised by Sigma-Aldrich.

Skin Biopsy and Staining for INIs

For select participants and family members with OPDM-related STR expansions, a 3 mm punch biopsy of normal skin was performed. The specimens were fixed in 10% formalin solution, embedded in paraffin and cut into 4-mm thick sections. Sections were stained with hematoxylin and eosin. Immunohistochemistry was performed using antibodies against p62 (#610833, BD Sciences, 1:400) and ubiquitin (#Z0458, Dako, 1:1000).

LAMA2 Sanger Sequencing and Reverse Transcription PCR (RT-PCR)

Segregation of a *LAMA2* variant were performed in L236 and her mother using primers (5′-CTTGTCTGGCTCCTGTCTGA-3′; 5′-ATTTGGAAAACAGTGATGCTTCA-3′) designed to amplify exon 19 and intron 19 (chr6:129287952–129288286 [hg38]) to produce a 335 bp amplicon. Amplicons underwent Sanger sequencing using Applied Biosystems BigDye Terminator cycle sequencing protocols. Sequences were visualised and analysed using Snapgene Software V7.1.

Skin biopsy-derived primary fibroblast lines were established from case L236 and an unrelated non-myopathy control individual. Fibroblast cell lines were maintained in culture medium comprising Dulbecco's Modified Eagle Medium (DMEM), 10% (v/v) fetal bovine serum (FBS), 1% (v/v) penicillin-streptomycin and 1% (v/v) L-glutamine (Gibco, Life Technologies). Fibroblasts were maintained in 5% CO_2 humidified air at 37°C. RNA extraction from fibroblasts was conducted using RNEasy Mini Kit (Qiagen) and reverse-transcribed template was prepared using iScript cDNA Synthesis Kit (Bio-Rad). PCR amplification of cDNA was conducted using a forward primer designed to anneal to the exon-exon boundary between *LAMA2* exons 17 and 18 (5′-CCCATCCAATAACTTTAGCCCA-3′), and a reverse primer designed to anneal to exon 21 (5′-GCGGTCACATTTCTTCCCTG-3′). The RT-PCR amplicons were size fractionated using a 2% (w/v) agarose gel, gel-purified using Isolate II PCR and Gel Kit (Bioline) and then Sanger sequenced using Applied Biosystems BigDye Terminator cycle sequencing protocols. Sequences were visualised and analysed using Snapgene Software V7.1.

CAPN3 RT-PCR

Blood was collected into PAXgene Blood RNA Tubes (BD Biosciences) for case L323 and a non-myopathy control. RNA was isolated using the PAXgene Blood RNA Kit (PreAnalytix, Qiagen), before reverse-transcription to cDNA with random hexamers using the SuperScript IV First-Strand Synthesis System (Invitrogen). PCR amplification of cDNA was conducted using a forward primer designed to anneal to exon 19 (5′-GTCCTGCCGTAGCATGATTG-3′) and one of several reverse primers designed to anneal to a potential pseudoexon within intron 23 (5′-CAAGAAGCAGTGGCTTACTG-3′), exon 24 (5′-GCATACATGGTGAGCTGCAG-3′) or the 3′ untranslated region of *CAPN3* (5′-GAGCATGACTGATGAGGTGC-3′). RT-PCR amplicons were separated using a 1.5% (w/v) agarose gel in 0.5× Tris-borate EDTA buffer along with a 100 bp DNA ladder (Invitrogen) and visualised using GelRed (Biotium) staining. Amplicon bands were gel-purified using the QIAquick PCR Purification kit (Qiagen) and Sanger sequenced using Applied Biosystems BigDye Terminator cycle sequencing protocols.

Extraction of HMW DNA from Muscle

For case L155, who had previously undergone a muscle biopsy, HMW genomic DNA was extracted from approximately 20 mg of skeletal muscle using the Nanobind PanDNA Kit (PacBio, Cat# 102-260-000) following the 'Extracting DNA from animal tissue using the Nanobind PanDNA kit' protocol (PacBio, Cat# 102-574-600). This muscle-derived DNA then underwent targeted ONT LRS following the protocol described in Methods. The proportion of large mtDNA deletions (arbitrarily defined as deletions > 1,000 bp) was calculated by dividing the number of reads containing such deletions by the total number of reads > 1,000 bp in length that aligned to the mtDNA genome.

Supplementary References

1. Stender, S., Chakrabarti, R.S., Xing, C., Gotway, G., Cohen, J.C. & Hobbs, H.H. Adult-onset liver disease and hepatocellular carcinoma in S-adenosylhomocysteine hydrolase deficiency. *Mol Genet Metab*. **116,** 269-274 (2015).
2. Baric, I. et al. S-adenosylhomocysteine hydrolase deficiency in a human: a genetic disorder of methionine metabolism. *Proc Natl Acad Sci USA*. **101**, 4234-4239 (2004).
3. Ghaoui, R. et al. Use of whole-exome sequencing for diagnosis of limb-girdle muscular dystrophy: outcomes and lessons learned. *JAMA Neurol*. **72**, 1424-1432 (2015).
4. Sullivan, P.J., Quinn, J.M.W., Ajuyah, P., Pinese, M., Davis, R.L. & Cowley, M.J. Data-driven insights to inform splice-altering variant assessment. *Am J Hum Genet*. **112**, 764-778 (2025).
5. Ruggieri, A. et al. Multiomic elucidation of a coding 99-mer repeat-expansion skeletal muscle disease. *Acta Neuropathol*. **140**, 231-235 (2020).
6. Yang, K. et al. Expanding the phenotype and genotype spectra of PLIN4-associated myopathy with rimmed ubiquitin-positive autophagic vacuolation. *Acta Neuropathol*. **143**, 733-735 (2022).
7. Llansó, L., et al. Repeat expansions in PLIN4 cause autosomal dominant vacuolar myopathy with sarcolemmal features. *Ann Clin Transl Neurol*. **12**, 2136-2151 (2025).
8. Hiatt, L. et al. STRchive: a dynamic resource detailing population-level and locus-specific insights at tandem repeat disease loci. *Genome Med*. **17**, 29 (2025).
9. Carnazzi, A., et al. Genetic variability in the PLIN4 gene: A new sequence duplication causing autophagic vacuolar myopathy. *Genes Dis*. **13**, 101849 (2025).
10. Eura, N. et al. Pathogenic CGG expansions in oculopharyngodistal myopathy exhibit distinct characteristics of each causative gene on the flanking sequences as well as methylation status. *Genome Med* **18**, 33 (2026).
11. Keegan, N.P., Wilton, S.D. & Fletcher, S. Breakpoint junction features of seven DMD deletion mutations. *Hum Genome Var*. **6**, 39 (2019).
12. Startek, M. et al. Genome-wide analyses of LINE-LINE-mediated nonallelic homologous recombination. *Nucleic Acids Res*. **43**, 2188-2198 (2015).
