# Supplementary material for: Targeted long-read sequencing enables comprehensive analysis of the genetic and epigenetic landscape of inherited myopathies
Source: Nat Commun. 2026 Jul 4;17:8324. doi: 10.1038/s41467-026-75144-z (PMC13470197; doi:10.1038/s41467-026-75144-z)
Supplement: Supplementary file 2 [file 41467_2026_75144_MOESM2_ESM.pdf]

Description of Additional Supplementary Files

Supplementary Data 1. List of genetic loci for programmable targeted Oxford nanopore long-read sequencing myopathy panel

Supplementary Data 2. Structural variant summary for myopathy long-read sequencing cohort

Supplementary Data 3. Myopathy gene lists/panels from PanelApp Australia and selected external laboratories (accessed January 2023)
