# Supplementary material for: Targeted long-read sequencing enables comprehensive analysis of the genetic and epigenetic landscape of inherited myopathies
Source: Nat Commun. 2026 Jul 4;17:8324. doi: 10.1038/s41467-026-75144-z (PMC13470197; doi:10.1038/s41467-026-75144-z)
Supplement: Supplementary file 3 [file 41467_2026_75144_MOESM6_ESM.pdf]

nature portfolio

Corresponding author(s): Ira W Deveson & Kishore R Kumar

Last updated by author(s): 23 June 2026

Reporting Summary

Nature Portfolio wishes to improve the reproducibility of the work that we publish. This form provides structure for consistency and transparency in reporting. For further information on Nature Portfolio policies, see our Editorial Policies and the Editorial Policy Checklist.

Statistics

For all statistical analyses, confirm that the following items are present in the figure legend, table legend, main text, or Methods section.

n/a	Confirmed	
☐	☒	The exact sample size (*n*) for each experimental group/condition, given as a discrete number and unit of measurement
☒	☐	A statement on whether measurements were taken from distinct samples or whether the same sample was measured repeatedly
☒	☐	The statistical test(s) used AND whether they are one- or two-sided<br>*Only common tests should be described solely by name; describe more complex techniques in the Methods section.*
☒	☐	A description of all covariates tested
☒	☐	A description of any assumptions or corrections, such as tests of normality and adjustment for multiple comparisons
☐	☒	A full description of the statistical parameters including central tendency (e.g. means) or other basic estimates (e.g. regression coefficient) AND variation (e.g. standard deviation) or associated estimates of uncertainty (e.g. confidence intervals)
☒	☐	For null hypothesis testing, the test statistic (e.g. *F*, *t*, *r*) with confidence intervals, effect sizes, degrees of freedom and *P* value noted<br>*Give P values as exact values whenever suitable.*
☒	☐	For Bayesian analysis, information on the choice of priors and Markov chain Monte Carlo settings
☒	☐	For hierarchical and complex designs, identification of the appropriate level for tests and full reporting of outcomes
☒	☐	Estimates of effect sizes (e.g. Cohen's *d*, Pearson's *r*), indicating how they were calculated

Our web collection on statistics for biologists contains articles on many of the points above.

Software and code

Policy information about availability of computer code

Data collection	During ONT sequencing, Readfish (v0.0.10dev2) was used for live target selection or rejection utilising a custom panel of 331 loci associated with genetic myopathies (Supplementary Table 1).
Data analysis	A variety of external and internal open source software packages were used in this study.<br>Citations and/or links to Github repositories are provided in the METHODS section.<br>Specific code/scripts used to perform the analysis is available on Zenodo (https://doi.org/10.5281/zenodo.19601174).<br>We developed a purpose-built, open source software package, d4z4ling (https://github.com/neysa-15/d4z4ling) for this study.<br><br>Data analysis involved the following additional open source software:<br>Buttery-eel running model dna_r10.4.1_e8.2_400bps_sup@v5.2.0 or dna_r10.4.1_e8.2_400bps_sup@v4.1.0 for ONT basecalling.<br>Pipeface (v0.10.2) nextflow pipeline for executing data other data analysis tools, including:<br>Minimap2 (v2.28-r1209) for alignment.<br>Clair3 (v1.0.9) and Deepvariant (v1.80) for calling small variants.<br>sniffles2 (v2.6.0) and cuteSV67 (v2.1.1) for calling SVs.<br>WhatsHap (v2.3) for phasing.<br>minimod (v0.3.0) for DNA methylation profiling.<br>variant effect predictor (VEP) (v112) for functional annotation of small variants and SVs.<br>LongTR (v1.0) for STR genotyping.<br>IGV Genome browser (2.19 or later) for data visualisation.<br>bcftools (v1.20 or later) for interacting with VCF files.

ExPASy web app (https://www.expasy.org/) for DNA to protein translation.
Mitoverse mtDNA-server (v2) for mitochondrial variant calling.

For manuscripts utilizing custom algorithms or software that are central to the research but not yet described in published literature, software must be made available to editors and reviewers. We strongly encourage code deposition in a community repository (e.g. GitHub). See the Nature Portfolio guidelines for submitting code & software for further information.

Data

Policy information about availability of data

All manuscripts must include a data availability statement. This statement should provide the following information, where applicable:

- Accession codes, unique identifiers, or web links for publicly available datasets
- A description of any restrictions on data availability
- For clinical datasets or third party data, please ensure that the statement adheres to our policy

Human participant data may be shared under restricted access to protect participant privacy. Anonymised data is available in dbGaP (accession: phs004623.v1.p1); enquiries for data access should be addressed to the corresponding authors K.R.K. and I.W.D. Enquiries will generally be reviewed within 2-4 weeks.

Research involving human participants, their data, or biological material

Policy information about studies with human participants or human data. See also policy information about sex, gender (identity/presentation), and sexual orientation and race, ethnicity and racism.

Reporting on sex and gender	In Supplementary Figure 1, we have reported on sex assigned at birth of a subset of our cohort based on participant self-reporting. No biologic sex- or gender-based statistical analyses or comparison were conducted in this study as they were not relevant. Where relevant, sex was considered during genetic analysis in this study e.g. analysis of the DMD gene which is located on the X chromosome. Sex of participants was determined by self report. Comparison of methylation within the cohort was not disaggregated for sex or gender because of the small size within each diagnostic category (FSHD1, FSHD2, FSHD1+2) in this study.
Reporting on race, ethnicity, or other socially relevant groupings	Where relevant, we have reported on participant self-reported ancestry for select participants/participant families. Participant self-reported ancestry informed haplotype analyses but no race-, ethnicity- or social group-based stastical analysis/comparisons were performed in this study.
Population characteristics	Participants were adults (> or = 18 years of age) with known (previously genetically diagnosed) genetic myopathy or had a myopathy that was suspected to be genetic in aetiology.
Recruitment	Adult (age ≥ 18 years) participants were recruited from patients referred to Concord Repatriation General Hospital and Neuroscience Research Australia from March 2023 to September 2025. A positive control cohort, Group 1, consisted of patients with genetically confirmed myopathies specifically selected to represent a range of different genetic and epigenetic variant types. An undiagnosed cohort, Group 2, consisted of consecutively recruited participants that met the following criteria: 1) history, examination and investigations (creatine kinase, neurophysiology, muscle imaging and/or muscle biopsy) suggestive of and concordant for a myopathic process; 2) myopathy was the predominant neurologic phenotype; 3) the treating/referring clinician assessed that a genetic cause for the myopathy was more likely that an acquired aetiology; and 4) the participant remained genetically unsolved (or incompletely solved) after appropriate conventional clinical genetic testing as determined by the treating/referring clinician. Where relevant, family members of probands were recruited for segregation studies.<br><br>Patients seen at or referred to tertiary referral centres have typically already undergone extensive investigations and diagnostic workup - therefore, in this population, most 'straightforward' genetic diagnoses have already been made, but also mimics of genetic myopathy (e.g. acquired myopathies, motor neuropathies, etc.) have likely been more thoroughly filtered out. Therefore, the performance of our assay/approach, in terms of overall diagnostic yield, may vary in other settings (e.g. in primary/secondary healthcare settings). Similarly, the diagnostic yield may vary in different age groups - for example in patients < 18 years of age, where genetic aetiologies for myopathy are likely more prevalent than acquired aetiologies for myopathy, diagnostic yield may be even better than in our adult cohort.
Ethics oversight	The protocols for this study were approved by the St Vincent's Hospital Human Research Ethics Committee (HREC/2019/ETH12538) and Royal Children's Hospital Melbourne Human Research Ethics Committee (HREC/95179/RCHM-2023).

Note that full information on the approval of the study protocol must also be provided in the manuscript.

Field-specific reporting

Please select the one below that is the best fit for your research. If you are not sure, read the appropriate sections before making your selection.

☒ Life sciences ☐ Behavioural & social sciences ☐ Ecological, evolutionary & environmental sciences

Life sciences study design

All studies must disclose on these points even when the disclosure is negative.

Sample size	We recruited 22 participants with a known diagnosis of genetic myopathy and 31 participants with a undiagnosed but suspected genetic myopathy over a period of ~2.5 years (2023-2025). Our study was exploratory and we did not perform any statistical analyses - therefore we did not perform any power calculations to determine target sample size.
Data exclusions	No data was excluded from analysis
Replication	Our was an exploratory study to determine the utility of our assay - therefore we performed sequencing with our assay and genetic analysis on all participants recruited to the study. We did not recruit a second validation cohort for replication. However, within our cohort, we demonstrated the reproducibility of our assay's ability to identify standard SNV/indel variants, structural variants, mtDNA variants, STR expansions and FSHD-related genetic & epigentic variants in multiple participants.<br><br>Skin biopsy immunohistochemistry and FSHD Southern blot results were taken from test performed in the course of patient clinical care and so these were not performed in replicate. Each RT-PCR experiment was repeated at least once and using combinations of different primers, - these yielded similar results, supporting validity and reproducibility of these results.
Randomization	Randomisation is not relevant to our study design as sequencing and genetic analysis was performed on all participants recruited to the study.
Blinding	Our investigators were not blinded during genetic analysis as knowledge of clinical features of each participant's myopathy (e.g. age of onset, pattern of distribution, family history/inheritance pattern, biochemical results, etc.) informs filtering and prioritisation of genetic variants.

Reporting for specific materials, systems and methods

We require information from authors about some types of materials, experimental systems and methods used in many studies. Here, indicate whether each material, system or method listed is relevant to your study. If you are not sure if a list item applies to your research, read the appropriate section before selecting a response.

Materials & experimental systems

n/a	Involved in the study	
☐	☒	Antibodies
☐	☒	Eukaryotic cell lines
☒	☐	Palaeontology and archaeology
☒	☐	Animals and other organisms
☐	☒	Clinical data
☒	☐	Dual use research of concern
☒	☐	Plants

Methods

n/a	Involved in the study	
☒	☐	ChIP-seq
☒	☐	Flow cytometry
☒	☐	MRI-based neuroimaging

Antibodies

Antibodies used	Antibodies against p62 (#610833, BD Sciences, 1:400) and ubiquitin (#Z0458, Dako, 1:1000) were used for staining of skin biopsy samples in a subset of participants with GGC expansion related disorders (i.e. OPDM) to identify the presence of intranuclear inclusions.
Validation	Anti-p62 (BD-Biosciences, #610833): https://www.bdbiosciences.com/en-eu/products/reagents/microscopy-imaging-reagents/immunofluorescence-reagents/purified-mouse-anti-p62-ick-ligand.610833<br>- validated by manufacturer for use in Immunohistochemistry<br>Anti-ubiquitin (#Z0458, Dako, 1:1000): https://www.citeab.com/antibodies/3382935-z0458-ubiquitin<br>- validated by manufacturer for use in immunohistochemistry<br><br>Additionally these antibodies have been used by multiple studies to detect intranuclear inclusions in skin biopsy of GGC expansion related disorders such as neuronal intranuclear inclusion disorder (NIID) which has clinicaly and genetic overlap with OPDM (e.g. PMID: 31178126, 24039026).

Eukaryotic cell lines

Policy information about cell lines and Sex and Gender in Research

Cell line source(s)	We generated a fibroblast line from one female participant in the study in order to perform RT-PCR on fibroblast RNA to validate a potential splice altering variant in LAMA2.
Authentication	Cell line authentication was not performed as the cell line was generated in-house

Mycoplasma contamination	Mycoplasma contamination of this cell line was not performed as the cell line was generated in-house
Commonly misidentified lines (See ICLAC register)	N/A

Clinical data

Policy information about clinical studies

All manuscripts should comply with the ICMJE guidelines for publication of clinical research and a completed CONSORT checklist must be included with all submissions.

Clinical trial registration	This study was not a clinical trial and so no clinical trial registration was performed
Study protocol	This study was not a clinical trial and so no clinical trial protocol was made publically available
Data collection	Clinical data was collected from participants at two neuromuscular/neurogenetics centres in Sydney, Australia (Concord Repatriation General Hospital and Neuroscience Research Australia) fomr March 2023 to September 2025.
Outcomes	Diagnostic accuracy (ability to identify/confirm previous known genetic findings; and also ability to newly identify previously unidentified causative genetic/epigenetic variants) was our overall outcome. We reported proportion of previously undiagnosed patients who received a new genetic diagnosis following our assay as a major outcome of our study. New genetic diagnosis were informed through use of American College of Medical Genetics criteria for variant interpretation and clinician consensus on match between genetic findings and phenotype.

Plants

Seed stocks	N/A
Novel plant genotypes	N/A
Authentication	N/A
