# Supplementary material for: Targeted long-read sequencing enables comprehensive analysis of the genetic and epigenetic landscape of inherited myopathies
Source: Nat Commun. 2026 Jul 4;17:8324. doi: 10.1038/s41467-026-75144-z (PMC13470197; doi:10.1038/s41467-026-75144-z)
Supplement: Supplementary file 4 [file 41467_2026_75144_MOESM8_ESM.pdf]

nature portfolio

Peer Review File

Targeted long-read sequencing enables comprehensive analysis of the genetic and epigenetic landscape of inherited myopathies

Corresponding Author: Dr Ira Deveson

This file contains all reviewer reports in order by version, followed by all author rebuttals in order by version.

Version 0:

Reviewer comments:

Reviewer #1

(Remarks to the Author)
Review of Targeted long-read sequencing enables comprehensive analysis of the genetic
and epigenetic landscape of inherited myopathies by Yeow and colleagues. The authors describe a targeted LRS assay and integrated bioinformatics framework to assess all known genetic/epigenetic causes of inherited myopathies. They provide a strong example of a single assay for evaluating genetic myopathies.

I assume Table 1 is prior diagnoses excluding FSHD1 and 2, which are in table 2. It may be helpful to the reader to be more explicit about this in the label of the table.

Regarding the insertion/expansion identified in exon 5 of PLIN4, did the authors check for the expansion in any of the population-level LRS datasets that are being generated? Colors, HGSVC/HPRC, and/or the 1KGP-LRSC may have data that could be used to help interpret this expansion. Looking at the first 500 1KGP-LRSC samples I see at least one individual who has 6 copies of the 99bp repeat. I'm happy to point the authors to this data (I'll sign the review).

Why did the authors use Readfish instead of adaptive sampling within minknow? Broad uptake of this method would be limited if adaptive sampling isn't working as well as readfish does. It would be helpful to the general reader to discuss why readfish was used and/or how minknow could be modified to perform similar to readfish. If there is data showing an improvement of readfish over adaptive sampling within minknow I think it should be provided to support the use of readfish here.

Within the methods the authors should provide context for why BLOW5 is used and that the workflow doesn't require conversion of pod5 files to blow5 for implementation/use of this method.

It's not clear why ethnicity is included in table 3. I don't see what it adds to the manuscript as the impact of ethnicity is not discussed in text (only mentioned for a couple of cases).

Minor comments:

I really like Figure 1A—it's a very nice way to show the characteristics of the group and variants. It would be helpful to note the cases that had a VUS identified in this figure.

Figure S1: I would suggest the authors use biologic sex or sex assigned at birth rather than gender given that those may not be concordant.

Figure S2C: we see lots of variability in coverage and N50 similar to what the authors show, but I'm not sure I've ever seen off-target read N50's so high as in L121 and L159. Can the authors comment on what happened with those samples?

Figure S3A: it may be best to color the reads by phase set (PS tag) instead of by haplotype (which I assume is what they're colored by). This would demonstrate that the variatns are on different haplotypes.

Danny Miller

(Remarks on code availability)
I was able to access and review the code.

Reviewer #2

(Remarks to the Author)
Summary

This manuscript presents a method to genetically diagnose myopathy patients using an adaptive sampling targeted long-read sequencing (LRS) approach and a curated myopathy panel combined with a bioinformatics pipeline. The authors introduce new methods, including d4z4ling for FSHD analysis, which integrates homologous region information and methylation data. The study demonstrates that targeted LRS outperforms gold-standard testing in several cases, enabling phasing, accurate sizing of tandem repeat expansions, and CNV breakpoint determination. Notably, the approach yielded new diagnoses in 29% of cases (9/31), primarily repeat expansions, with one D4Z4 contraction and one short indel. These new diagnoses often resolved long diagnostic odysseys (mean 14 years).

The targeted LRS panel is designed for flexibility and can be readily expanded to include new genes or loci as they are identified. This approach is also broadly applicable to other genetic disorders, particularly those with multiple causative genes, known tandem repeat or structural variant contributions, or methylation involvement—such as ataxia and epilepsy. Consequently, its potential impact extends well beyond the myopathies described here. By focusing sequencing and analysis on clinically relevant loci while leveraging the advantages of long reads for complex variant detection, this methodology has the potential to reduce both testing costs and time to diagnosis for inherited myopathies.

Overall Assessment

This is a strong and innovative study that demonstrates the clinical utility of long-read sequencing for neuromuscular disorders with the potential for broader impact in genetic disease diagnosis. The authors demonstrate strong expertise in both long-read sequencing methodology and the clinical genetics of myopathies. The adaptive sampling approach and integration of methylation analysis are particularly impactful. However, additional detail on methods and comprehensive data/code availability are essential to ensure reproducibility and transparency.

Major comments

1. Data Availability
The sequencing data is described as available upon request. This limits reproducibility and secondary analysis. There is also concern that the data will be lost over time, despite the research team's best efforts to prevent this. We strongly recommend depositing raw or processed data in a controlled-access repository such dbGAP, the European Genome-phenome Archive (EGA), or a suitable equivalent. Also, any such data repository should indicate the expected timeline for data-access requests.

2. Validation Cohort
Were the same samples used to develop d4z4ling and validate the pipeline? If so, please note this in the relevant results/methods section and note the limitation in the discussion.

3. Code availability and method detail
Is there an end-to-end pipeline available, or was an existing pipeline (e.g., pipeface) modified? Please clarify and provide code covering all stages. In particular, the later stages of variant annotation and interpretation do not appear to be covered by the linked code, and there is insufficient detail to reproduce this step (see also below).

4. SV/CNV variant discovery
It would be helpful for the authors to clarify the performance of the ONT long-read assay for detecting CNVs/SVs that span multiple genes—particularly events that extend beyond the targeted regions but still encompass one or more genes included in the panel. What is the largest SV/CNV that you can reliably detect and report using Sniffles and cuteSV with your current read length/coverage and alignment settings? What are the types of SVs analyzed?

5. SV/CNV variant prioritization
The methods paragraph describing “variant prioritization” needs additional methodological detail and a clearer scope. It is currently ambiguous whether “variants” refers to SNPs/indels only or includes SVs/CNVs as well, and if both are included, which criteria apply to which variant class. We reviewed the PipeFace pipeline but could not locate documentation describing the allele-frequency annotation procedure, particularly for SVs.
In addition, the process used to add allele frequency (AF) annotations should be described explicitly, including: how AF was computed, whether AF was computed separately for SNPs/indels and SVs. Did you consider additional, larger allele frequency annotation sources for SVs, such as TopMed and CCDG, or tools like AnnotSV or SVAFotate that combine AF annotations from different sources? Finally, the statement “SVs intersecting protein-coding genes were prioritised” is

underspecified. Is the predicted gene impact used in SV filtering/prioritization beyond gene intersection? For example, do you explicitly filter or rank SVs by categories such as predicted pLoF, copy gain, intragenic exonic duplications, or SVs affecting promoters/enhancers/UTRs? If so, please describe the annotation tools/criteria and how these impact categories are applied in filtering.

6. Methylation analysis
The integration of methylation with allele size is valuable, especially for interpreting asymptomatic carriers, as seen in your case vignettes. Outside of d4z4ling, is methylation analysis automated or manually reviewed for specific loci? Could you please clarify the workflow.

7. D4Z4 read-level methylation analysis
In Figure 2b, read-level methylation data appears to be averaged across the entire read. This likely dilutes the signal as reads could span various regions in addition to D4Z4. Could you please clarify if this is the case? If that is the case, we suggest an alternative approach. Your method already identifies the D4Z4 region in each read, so you could use that to identify the methylation level in that specific region. Consider refining the methylation analysis/visualization by reporting % methylation within the relevant target region(s) (e.g., the defined D4Z4 methylation region) rather than per read across the entire read.

8. d4z4ling GitHub reference
The d4z4ling GitHub repository references a separate preprint (https://www.medrxiv.org/content/10.1101/2025.04.24.25326320v1). Could you please clarify the relationship between the current manuscript and this publication? Did the data inform the development of the current method?

9. Citation(s) missing for genomic coordinates
“a custom panel of 331 loci associated with genetic myopathies (Table S1). These loci were chosen based on a literature review performed in January 2023, focusing on genetic myopathic disorders, supplemented by review of existing myopathy gene panels in PanelApp and from diagnostic genomic laboratories (Invitae, Blueprint Genetics and PathWest).”
The source(s) should be provided for the genomic coordinates in Supp Table 1, pathogenic thresholds for repeat loci, and any other similar information used as input into the panel creation. We suggest providing a citation/source column for Supp Table 1 if the sources were distinct papers/resources. Alternatively, if most regions were from the same or a small number of sources, you could simply cite them in the methods section with links if relevant.

10. Genomic Coordinates
Please add genomic coordinates to all relevant figures or their legends, e.g., Figs 2a, 4c, 4d S8. If there are relevant genomic features within the figure, the exact positions of those would also be useful. For example, the specific coordinates of the FSHD locus features. Also, add the coordinates for all methylation regions identified by you or pulled from the literature, ideally in an easy-to-parse format, similar to Table S1. There is also a mixture of hg38 and T2T-chm13 coordinates used throughout the paper. So, double-check that the reference genome is clear for every coordinate, or choose a single coordinate system.

Minor Comments

1. Confirm whether the pipeline was tested on WGS data. Is it specific to ONT (not PacBio)?

2. Define “pseudodominance” (multiple definitions exist in the literature).

3. In Figure S1, the stacked bar plot for “age at onset” and “disease duration” implies that the sum equals current age, but this is not stated. Please clarify. If the intent is not to display the current age, consider an alternative visualization (e.g., separate bars or onset-to-current age segment plots).

4. On page 6, the paragraph about cases L246 and L247. I found this challenging to conceptualize. Could you please provide a sentence or two overall summary and/or a supplementary figure showing why the previous testing was incorrect.

5. Figure 2 has a lot of colours, resulting in a need to check the legend multiple times when interpreting it. You could simplify by using labelled boxes rather than background colour for the sample categories in panels c and e.

6. Is 50% methylated significant? Why was this value chosen to highlight in Figures 2b and similar? Can you provide some general guidance on how to read these plots? If most points fall in a specific quadrant, is that important?

7. On page 6, final paragraph, are you implying that the D4Z4 contraction on 10A176T observed in L117 is possibly pathogenic but with a different phenotype? Could you please clarify/elaborate?

8. Page 6, could you please define “permissive”? It is somewhat implied by context, but a more explicit definition would be helpful.

9. What proportion of the curated panel regions achieved sufficient sequencing depth across samples? The current analysis (Fig S2b) considers depth at the per-sample level, not per region. Were certain regions consistently undersampled? It is useful to know if specific regions are undersampled; the overall sequencing may have to be increased to ensure all relevant

regions are sequenced to the required depth.

10. In Supplementary Table 1, PLIN4 is listed under “other genetic myopathies”. It may fit more naturally grouped with the tandem repeat disorders, as it is a VNTR locus.

This review was co-authored by Harriet Dashnow and Elbay Aliyev

(Remarks on code availability)
See "Comments for Author"

Reviewer #3

(Remarks to the Author)
I co-reviewed this manuscript with one of the reviewers who provided the listed reports. This is part of the Nature Communications initiative to facilitate training in peer review and to provide appropriate recognition for Early Career Researchers who co-review manuscripts.

(Remarks on code availability)
See comments from author.

Reviewer #4

(Remarks to the Author)
The study by Yeow and Reis et. al. explores the utility of using Oxford Nanopore long read sequencing in unsolved neuromuscular diseases (NMD). They developed an optimization protocol for DNA sample preparation and then targeted sequencing of a panel of NMD genes. In addition, specialized software called d4z4ling was developed to accurately determine number of D4Z4 repeats associated with FSHD. Lastly, methylation of these regions were also explored. They were able to achieve 29% increase in diagnosis rate among myopathy patients that were undiagnosed from clinical genetic testing. The majority of these discoveries were repeat expansions in OPMD genes and FSHD cases with D4Z4 repeat contraction and/or changes surrounding in methylation.

The manuscript is very well written and figures were put together very nicely. The github repo looks well organized, which is extremely rare for most academic papers!

Major comments

1. The methods proposed in this manuscript would receive wider adoption if it was applied on a control group of data of unaffected individuals. There should be a set of publicly available data this could be applied to show the size distribution of STR in genes, common deletions in introns known in the population and also D4Z4 repeat distribution in the population. If there is also trio data available to look at transmission of these events. This would add to the downsampling result that was presented. Or does this approach require high coverage sequencing only achieveable through a targeted panel?

2. There are a few very feasible missed opportunities that this study did not explore with the data generated:
a. Sequencing data offers more information than just repeat size, which is really the only thing that southern blotting and PCR methods can provide. It is hypothesized by others that sequence diversity/heterogenity in the repeat units may contribute to pathogenicity.
b. Any interesting methylation pattern changes in genes specially in unsolved cases. It may be implied that there were none to comment but at least explicitly mention this.
c. Large structural variants detected. Its assumed that no overlapping coding regions were detected as they would have been explicitly mentioned. However, it would be good to mention any non-coding structural variants detected and whether they were common in the population. This highlights the utility of this approach to detect SV even though for this cohort none were considered causal.

3. It is clearly demonstrated in the manuscript that ONT long read sequencing can accurately detect STRs in OPMD genes and also FSHD D4Z4 repeats. The (?)additional new diagnostic finding in LAMA2 should discuss why it was missed by clinical sequencing as this may be valuable for readers to learn.
a. The small intronic deletion in LAMA2 is close enough to an exon (Figure 5C) that it should have been detected by exome or panel sequencing. It would be good for the authors to show what the existing NGS data looks like for this sample.

4. The variant filtering thresholds used could be considered very permissive and would be good for the authors to comment/justify on the following:
a. gnomAD allele fequency threshold of 0.01
b. SpliceAI threshold of 0.2. The candidate splice variants in L323 within CAPN3 have quite low prediction values. Noted that the authors did mention it will require further validation.

5. Distinguishing between 4qA, 4qB and 10qA haplotypes is important for determining pathogenicity of D4Z4 contractions. However, there is little explicit information on how this classification is achieved in the context of LRS through using d4z4ling. Mentioning restriction sites in Figure 3I lacks details. Shouldn't the tool be looking for a set of unique sequence or patten of sequences rather than just a short restriction enzyme site/sequence occurring by chance all over the genome?

6. Case L121 was presented to not have FSHD1 due to having 11 copies of D4Z4 instead of the magical threshold of 10 copies. How well established is the 10 copy pathogenicity threshold and the underlying biological mechanism that disease is caused when this threshold is crossed? Note this is different to the point made in the manuscript, which related to incorrectly counting 11 repeats (via SB) when the true number is 10 repeats. Putting this in perespective of the patient to miss out on exciting therapies in clinical trials because no one dared to challenge the 10 copy threshold and the underlying evidence!
a. Is Figure 3L mislabelled/typo as it references L147, should it be L121 instead?

Minor comments
1. Can the d4z4ling be used on PacBio long read sequencing or easily adapted? This could increase it's more wide usage.

2. The concept of on target and off target presented in Figure S2a may need to be clearly explained to readers. How can off targets occur in the context of using ONT and Readfish as many readers may be unfamiliar.

3. Coverage presented in Figure S2b is quite variable. Are the samples with coverage below 100x correlated to any DNA QC metric? Run time or adjusted software settings?

4. The authors should consider discussing and giving their thoughts/opinion on the value of targeted long read sequencing compared to short read WGS and 30x (PacBio) long read sequencing to increase the diagnostic yield on exome/panel negative samples. For example can the things detected in this study be detected using short read WGS or on a 30x PacBio sequenced genome? Or are the higher targeted coverages necessary? In addition, there is mention of cost effectiveness of this approach but no comparison with short read WGS, is it about the same price or cheaper? Most people would not know tis.

5. The authors should consider discussing using hypomethylation in combination with any variants detected in known FSHD2 associated genes to resolve VUS.

6. The authors should consider mentioning some of the challenges there may be to interpret variants/changes detected by this approach where there is no good population reference to compare against. This would be a challenge within known and novel disease genes.

7. Figure panels labels are in lower case letters, while the figure caption text uses capitalized letters.

(Remarks on code availability)
As noted in the intro summary there is a great github repo for d4z4ling.

1. Looking at the README it is extremely confusing without context regarding being a NCI user and the IF89. It makes absolutely no sense at the moment. It may be irrelevant to running it but authors should clarify.
2. There are still hard coded lines in the code that hasn't been corrected. For example: https://github.com/neysa-15/d4z4ling/blob/main/fshd1_annotate_fasta_script.sh#L155
3. Although the code for d4z4ling is available, there is no reference to additional repository containing the code used to generate figures and analyses presented in the manuscript. Noted that the underlying patient sequencing data can't be shared but the code used to process the data should be along with any processed data that can be shared.

Reviewer #5

(Remarks to the Author)
I co-reviewed this manuscript with one of the reviewers who provided the listed reports. This is part of the Nature Communications initiative to facilitate training in peer review and to provide appropriate recognition for Early Career Researchers who co-review manuscripts.

(Remarks on code availability)
This paper combines targeted long-read sequencing (ONT LRS) with Oxford nanopore technology to achieve comprehensive genetic and epigenetic characterization of hereditary myopathy. This study addresses an unmet need in myopathy diagnosis by overcoming the limitations of next-generation sequencing (NGS) and traditional methods (such as Southern Imprinting [SB] and Multiple Linkage-Dependent Probe Amplification [MLPA]) in resolving repetitive regions, structural variations, and epigenetic modifications. The development of the d4z4ling software for analysis of cheek-shoulder suprahumeral muscular dystrophy (FSHD) and its significant advantage of increasing the diagnostic rate by 29% in genetically unresolved cases are also noteworthy. However, several issues remain to be addressed to validate these

findings, support their innovation, and confirm clinical transferability.

1. The selection basis for the 331 loci in the target panel is not clearly stated. It is necessary to clarify whether these loci cover all known pathogenic genes for hereditary myopathy as of March 2023 and to provide evidence of consistency with authoritative databases such as OMIM and PanelApp. This will help confirm the rationality and comprehensiveness of the panel design.

2. Performance validation of the self-developed d4z4ling software, the core innovation of this manuscript in D4Z4 copy counting, haplotype identification, and methylation analysis, is not yet sufficient for clinical application. Large-scale blind comparison data with existing gold standards (such as pulsed-field gel electrophoresis (PFGE-SB) and optical genome localization) are not provided. Specifically, it is not stated whether the specificity for identifying rare haplotypes (such as 10A176T) has been validated in independent cohorts or experiments.

3. The manuscript mentions that the panel can be updated by adding genomic coordinates (e.g., newly added ABCD3 sites), but the detection performance of the updated panel for new sites is not validated. During the study, only 26 of the 53 newly added ABCD3 sites were sequenced, and data on their detection sensitivity and specificity are lacking.

4. The manuscript reports two cases of misdiagnosis/missed diagnosis of SB: L117 (10qA, 10-fold shrinkage) and L147 (4qA, 4-fold shrinkage), but the underlying technical reasons are not analyzed in depth. Regarding the interference mechanism of the 10A176T haplotype leading to false positives in restriction endonuclease sites (SB), it is currently unclear whether it has been verified through in vitro restriction enzyme digestion experiments. Furthermore, the false positive/false negative rate of SB in the FSHD cohort was not statistically analyzed, therefore the generalizability of the corrective effect of LRS in real-world clinical scenarios remains unexplained.

5. The hypothesis that "11-copy 4qA D4Z4 binding to hypomethylation may be pathogenic" is based on only one undiagnosed case, and the chain of evidence is extremely weak. Although hypomethylation is a common feature of FSHD1 and FSHD2, the authors still need to clarify whether the hypomethylation level (median ± 2×/2/MAD) in this case falls entirely within the range defined for FSHD1 in the manuscript. Simultaneously, functional experiments should be added to support pathogenicity (e.g., detection of DUX4 expression levels in muscle tissue or fibroblasts). Without these data, the pathogenicity of the 11-copy 4qA D4Z4 array remains speculative.

6. The authors reported that the FSHD2 family (L153/L154/L438) exhibits 5-copy 4qA cis D4Z4 amplification combined with the SMCHD1 variant, but failed to analyze the synergistic effect between the two. According to the empirical formula of Lemmers et al., this 5-copy amplification requires the SMCHD1 variant to be pathogenic, but the authors did not verify whether this amplification is pathogenic only in the absence of the SMCHD1 variant, thus the pathogenicity threshold for 5-copy amplification could not be determined.

7. Only one case of FSHD1+2 (L439) was reported, and the combined effect of "7-copy 4qA contraction combined with SMCHD1 deletion" was not analyzed in detail. It is currently unclear whether the D4Z4 methylation level in this case (more severe hypomethylation in the contraction allele) is related to the severity of the clinical phenotype (e.g., age of onset, muscle strength score). Furthermore, there is a lack of laboratory evidence to confirm whether this compound variant leads to a significantly higher DUX4 expression level than in FSHD1/2 cases alone.

8. The FSHD cohort sample size is small, including 10 FSHD1 cases, 3 FSHD2 cases, and 1 FSHD1+2 case. All FSHD2 cases belong to the same family, making it impossible to verify the applicability of this technology to sporadic FSHD2 cases. Furthermore, it is unclear whether these 10 FSHD1 cases encompass different clinical subtypes (e.g., mild cases with only facial and shoulder involvement, and severe cases with proximal limb involvement), thus the applicability of the software to different phenotypes cannot be confirmed.

9. The manuscript mentions that d4z4ling can achieve allele-specific methylation detection, but does not explain the method by which the software calculates methylation frequencies (e.g., the threshold for methylation calls per read). The lack of consistency data with the gold standard bisulfite sequencing (BSS) makes it impossible to confirm the reliability of the defined FSHD1 methylation range (12.42–45.58%).

10. The evidence chain for inferring a GIPC1-related OPDM2 association based on 7 SNPs is weak. It is necessary to supplement the linkage disequilibrium coefficient (D' value) between this SNP haplotype and GIPC1 STR amplification. If the linkage is not strong, haplotype association is meaningless.

11. The paper emphasizes that LRS can simultaneously detect FSHD1/2/1+2 and other myopathies, but fails to address the core obstacles to clinical adoption. The most important issue is the lack of a direct comparison of cost, cycle time, and diagnostic rate between LRS assays and combined SB+NGS protocols. These data are crucial for clinical decision-making and technology adoption.

Version 1:

Reviewer comments:

Reviewer #1

(Remarks to the Author)
The authors have addressed my comments, I have no additional comments.

Danny Miller

(Remarks on code availability)

Reviewer #2

(Remarks to the Author)
Thank you for thoroughly addressing all of our comments. In particular, we appreciate your attention to reproducibility by uploading genomic data to dbGAP (I assume this will be released upon publication?) and providing computational workflows.

We have no additional comments.

This review was co-authored by Harriet Dashnow and Elbay Aliyev

(Remarks on code availability)

Reviewer #3

(Remarks to the Author)
I co-reviewed this manuscript with one of the reviewers who provided the listed reports. This is part of the Nature Communications initiative to facilitate training in peer review and to provide appropriate recognition for Early Career Researchers who co-review manuscripts.

(Remarks on code availability)

Reviewer #4

(Remarks to the Author)
The authors went above and beyond to address all the comments. The work to include results from control/reference is a large additional piece of work and appreciate them adding this extra information. In addition, including a separate repo with analysis/plotting code associated with figures is now aligned with gold standard for human genetics publications. Also, in preparing this they put placeholders for file paths that needed to be edited so showed they learned from the initial feedback!

(Remarks on code availability)
The authors have made all the required changes from the initial review.

Open Access This Peer Review File is licensed under a Creative Commons Attribution 4.0 International License, which permits use, sharing, adaptation, distribution and reproduction in any medium or format, as long as you give appropriate credit to the original author(s) and the source, provide a link to the Creative Commons license, and indicate if changes were made.
In cases where reviewers are anonymous, credit should be given to 'Anonymous Referee' and the source.
The images or other third party material in this Peer Review File are included in the article's Creative Commons license, unless indicated otherwise in a credit line to the material. If material is not included in the article's Creative Commons license and your intended use is not permitted by statutory regulation or exceeds the permitted use, you will need to obtain permission directly from the copyright holder.
To view a copy of this license, visit https://creativecommons.org/licenses/by/4.0/

Response to Reviewer Comments

We thank all reviewers for their constructive feedback. In addition to point-by-point responses below, we have completed additional experimental work needed to upgrade two cases with variants of uncertain significance to provisional genetic diagnoses. This has been added to our revised manuscript as follows:

- RT-PCR studies in L323 to confirm pathogenicity of *CAPN3* c.2440-75C>G variant
- ONT LRS of muscle derived-DNA in L155 (who harboured biallelic variants in *DGUOK*) to confirm the participant has multiple large mtDNA deletions in muscle DNA but not blood DNA, supporting a diagnosis of multiple mtDNA deletion myopathy, likely related to the *DGUOK* variants
- Accordingly, the overall diagnostic rate in Group 2 of our cohort has been updated to 35% (11/31)

Actions: Additional data regarding L323 and L155 added to **Results** and **Table 3**, diagnostic rate updated in **Abstract**, **Results** & **Discussion** and new **Figure 5** subpanels and new **Supplementary Figures S11-12** added. **Supplementary Methods** updated.

Reviewer 1

1.0. Review of Targeted long-read sequencing enables comprehensive analysis of the genetic and epigenetic landscape of inherited myopathies by Yeow and colleagues. The authors describe a targeted LRS assay and integrated bioinformatics framework to assess all known genetic/epigenetic causes of inherited myopathies. They provide a strong example of a single assay for evaluating genetic myopathies.

We thank the reviewer, Dr Miller, for their constructive review of our manuscript. Responses to Dr Miller's specific comments are provided below.

1.1. I assume Table 1 is prior diagnoses excluding FSHD1 and 2, which are in table 2. It may be helpful to the reader to be more explicit about this in the label of the table.

To clarify this, we have changed the title of **Table 1** from "ONT LRS results in myopathy patients with prior known genetic diagnoses" to "**ONT LRS results in myopathy patients with prior known genetic diagnoses (excluding FSHD)**".

Actions: Title of **Table 1** modified.

1.2. Regarding the insertion/expansion identified in exon 5 of PLIN4, did the authors check for the expansion in any of the population-level LRS datasets that are being generated? Colors, HGSVC/HPRC, and/or the 1KGP-LRSC may have data that could be used to help interpret this expansion. Looking at the first 500 1KGP-LRSC samples I see at least one individual who has 6 copies of the 99bp repeat. I'm happy to point the authors to this data (I'll sign the review).

We thank the reviewer for this good suggestion. We examined the first 500 samples from the 1KGP-LRSC cohort to identify individuals with *PLIN4* expansions. The hg38 reference contains 29 × 99bp repeat units at the *PLIN4* locus. In the TRGT VCF aligned to hg38, we identified four individuals with expansions larger than 6 × 99bp additional copies (i.e. >=35 total copies): HG0335 (594bp, +6 copies); HG02884 (693bp, +7 copies); HG03072 (967bp, +9 copies); HG04076 (2087bp, +21 copies). Inspection of the corresponding BAM files confirmed the presence of these insertions, although the very large insertion in HG04076 was supported by only a single read, suggestive of an error. These data indicate that *PLIN4* expansions, such as those observed in L144 and L286 (+10 copy; 39 × 99 bp), are rare but do occur among the 'normal' population. STRchive reports a benign range of 29 to 31 repeat units and a pathogenic range of 37 to 50 units. Given that the father is asymptomatic, this suggests that expansions near the lower end of the pathogenic range may show incomplete penetrance. At the reviewer's suggestion, we have added this analysis and further discussed the relevance of the observed *PLIN4* VNTR expansions in our revised manuscript.

Actions: Analysis of 1KGP-LRSC cohort added to **Supplementary Note 2**; further discussion on the relevance of *PLIN4* expansion included in **RESULTS** section and elaborated in **Supplementary Note 2**.

1.3. Why did the authors use Readfish instead of adaptive sampling within minknow? Broad uptake of this method would be limited if adaptive sampling isn't working as well as readfish does. It would be helpful to the general reader to discuss why readfish was used and/or how minknow could be modified to perform similar to readfish. If there is data showing an improvement of readfish over adaptive sampling within minknow I think it should be provided to support the use of readfish here.

We used ReadFish in this study rather than MinKNOW 'adaptive sampling' because: (i) we prefer to use open source academic software wherever possible; and (ii) our lab has been using ReadFish for a long time and have stuck with it out of familiarity. ReadFish and adaptive sampling are now effectively equivalent and the user could choose either option without significantly impacting their results. We have clarified this in the **METHODS** section and suggested the user can choose either software, depending on their preference.

Actions: Sentence added to the **METHODS** section noting the equivalence of ReadFish vs MinKNOW adaptive sampling.

1.4. Within the methods the authors should provide context for why BLOW5 is used and that the workflow doesn't require conversion of pod5 files to blow5 for implementation/use of this method.

It is not compulsory to convert raw ONT data files from POD5 to BLOW5 format in order to use our downstream analysis workflow or other standard ONT analysis pipelines. We do this to take advantage of the significant, enduring performance advantages of the BLOW5 format, especially when running ONT basecalling on HPC or Cloud computing environments (see PMID:41100284). Users should be aware that running live, high- or super-accuracy basecalling on their PromethION while performing adaptive sampling may hurt the performance, so it's best to perform basecalling offline once the experiment is complete. We have clarified both these points in our revised manuscript.

Actions: Sentences added to **METHODS** section noting POD5 to BLOW5 data conversion is optional; reasons for doing so; reasons to avoid live basecalling during experiment.

1.5. It's not clear why ethnicity is included in table 3. I don't see what it adds to the manuscript as the impact of ethnicity is not discussed in text (only mentioned for a couple of cases).

We have now removed ethnicity information from **Table 3**. We refer to ethnicity in the main text for several cases where this is relevant, e.g. cases with *NOTCH2NLC* expansions, which are predominantly found in people of East Asian ancestry. We agree this is not useful info to include for all cases.

Actions: Ethnicity information removed from **Table 3**, some of which has been moved to text in the **RESULTS** section.

1.6. Minor comments:
I really like Figure 1A—it's a very nice way to show the characteristics of the group and variants. It would be helpful to note the cases that had a VUS identified in this figure.

Thank you for the feedback on **Figure 1A**. After some consideration we decided not to add VUSs to the figure. We have highlighted the strongest VUS candidates in **Supplementary Note 1**. However, at least one VUS was detected in every participant (with varying degrees of significance). Therefore, we feel it wouldn't improve the figure.

Actions: None.

1.7. Figure S1: I would suggest the authors use biologic sex or sex assigned at birth rather than gender given that those may not be concordant.

Thank you for this suggestion; we have made this change.
Actions: 'Gender' replaced with 'sex assigned at birth' in **Supplementary Figure 1**.

1.8. Figure S2C: we see lots of variability in coverage and N50 similar to what the authors show, but I'm not sure I've ever seen off-target read N50's so high as in L121 and L159. Can the authors comment on what happened with those samples?

For those two samples there was a temporary issue that caused ReadFish to stall for several hours during the experiment. During this time sequencing proceeded without rejection of off-target reads. The issue was identified and rectified during the experiment but the resulting libraries have a portion of off-target reads that are much longer than usual (because they weren't subject to rejection). This is now noted in the figure legend.

Actions: Supplementary Figure 2C legend updated to explain issue affecting L121 and L159.

1.9. Figure S3A: it may be best to color the reads by phase set (PS tag) instead of by haplotype (which I assume is what they're colored by). This would demonstrate that the variants are on different haplotypes.

Thank you for this suggestion; we have made this change.

Actions: Supplementary Figure 3A updated with reads coloured by PS tag.

1.10. (Remarks on code availability)
I was able to access and review the code.

We thank the reviewer for reviewing the code.

Actions: None.

Reviewers 2 & 3

2.0. Overall Assessment
This is a strong and innovative study that demonstrates the clinical utility of long-read sequencing for neuromuscular disorders with the potential for broader impact in genetic disease diagnosis. The authors demonstrate strong expertise in both long-read sequencing methodology and the clinical genetics of myopathies. The adaptive sampling approach and integration of methylation analysis are particularly impactful. However, additional detail on methods and comprehensive data/code availability are essential to ensure reproducibility and transparency.

We thank the co-reviewers Dr Dashnow and Dr Aliyev for their constructive review of our manuscript. Responses to their specific comments are provided below.

2.1. Data Availability
The sequencing data is described as available upon request. This limits reproducibility and secondary analysis. There is also concern that the data will be lost over time, despite the research team's best efforts to prevent this. We strongly recommend depositing raw or processed data in a controlled-access repository such dbGAP, the European Genome-phenome Archive (EGA), or a suitable equivalent. Also, any such data repository should indicate the expected timeline for data-access requests.

We thank the reviewers for drawing our attention to these repositories, which we agree are beneficial for reproducibility. While we cannot share patient sequencing data publicly, controlled access through dbGAP is consistent with our ethics protocol. We have therefore deposited all sequencing data to dbGAP under accession number phs004623.v1.p1. We have noted this in the **Data Availability** statement.

Actions: Patient sequencing data deposited to dbGAP under controlled access; **Data Availability** statement updated.

2.2. Validation Cohort
Were the same samples used to develop d4z4ling and validate the pipeline? If so, please note this in the relevant results/methods section and note the limitation in the discussion.

FSHD positive control samples used in this study were independently validated by Southern blot, and these same samples were used during the development of *d4z4ling*. We agree that this is a limitation of the current study. For this reason we did not report performance metrics such as sensitivity or specificity for d4z4ling, as a proper assessment would require an external, naive validation cohort. Unfortunately, the number of available positive controls was limited, so we could not meaningfully split the cohort into independent training and validation sets. Rigorous analytical validation is the next step for us. We have now clarified this in the **METHODS** and noted it explicitly as a limitation in the **DISCUSSION**.

Actions: Limitations regarding FSHD positive control cohort acknowledged in the **METHODS** and **DISCUSSION**.

2.3. Code availability and method detail
Is there an end-to-end pipeline available, or was an existing pipeline (e.g., pipeface) modified? Please clarify and provide code covering all stages. In particular, the later stages of variant annotation and interpretation do not appear to be covered by the linked code, and there is insufficient detail to reproduce this step (see also below).

The analysis framework used in this study consists of two primary pipelines, together with a downstream visualisation and interpretation layer. We have provided additional information in the **METHODS** section to clearly describe each component and how data flow between them. Links to the repositories, including full documentation, are now provided in the **METHODS** and **CODE AVAILABILITY** sections.

Pipeface is the core analysis pipeline and performs generic read alignment, small variant and structural variant calling, STR genotyping, phasing, and basic functional annotation of SNVs/indels and SVs. Pipeface comprises a range of open source software packaged into a Nextflow pipeline. The Pipeface repository is publicly available and referenced in the **METHODS** section.

Pipeface produces various standard output files (VCF, BAM, etc) for downstream analysis and interpretation, with whatever platform the user prefers. To facilitate variant visualisation, filtering, and interpretation, we use an interactive R Shiny application that we have developed, called ***PuzzleApp***. This does not perform primary analysis or annotation itself, but provides an interface for prioritizing variants called by Pipeface. PuzzleApp is operated through a GUI with all parameters/filters adjusted flexibly on a case-by-case basis. We cannot provide generic code for this process because every case is different. However, we have now referenced it in the **METHODS** section (github link to be added prior to publication), and explained the logic and basic initial filtering criteria that we followed in this study.

We note that PuzzleApp is under active development and the Github repo is not yet public. We will make the repo public at the time of publication and add a link to the **METHODS** section. If the reviewers would like to access PuzzleApp prior to that, please contact us by email so we can arrange private access.

d4z4ling is a separate specialised pipeline intended only for FSHD analysis. It operates independently to Pipeface and PuzzleApp and includes both analytical and visualisation components to perform D4Z4 repeat sizing, haplotyping, and single-molecule methylation profiling. The d4z4ling repository is likewise publicly available and referenced.

Actions: Use of *PuzzleApp* for variant interpretation described in the **METHODS**; new sentences added to the **METHODS** to clarify the components of the bioinformatics workflow.

2.4. SV/CNV variant discovery
It would be helpful for the authors to clarify the performance of the ONT long-read assay for detecting CNVs/SVs that span multiple genes—particularly events that extend beyond the targeted regions but still encompass one or more genes included in the panel. What is the largest SV/CNV that you can reliably

detect and report using Sniffles and cuteSV with your current read length/coverage and alignment settings? What are the types of SVs analyzed?

Our current target panel is ~30 Mbase in size and includes complete gene bodies plus ±50 kb flanking regions. We typically detect on the order of ~200–300 SVs per sample overlapping the targeted regions. SVs called by Sniffles and cuteSV include all standard classes (INS, DEL, DUP, INV and BND), with deletions and insertions comprising the majority of calls. Although Sniffles and cuteSV do call occasional SVs that are fully or partially outside of the target regions, these are almost always filtered out due to insufficient evidence. This effectively limits us to detection of SVs fully-contained within a single target region. While this imposes an upper size limit for some genes, large genes like *DMD* (>2 Mbase target size) permit fully contained large SVs, even on the scale of 100s of kb. Such events are rare and almost all SVs detected fall below ~50 kb. The restriction of variant detection to target regions is now clarified in our **METHODS** section and noted as a limitation in the **DISCUSSION**.

In this study, we have not used an additional CNV-specific caller beyond Sniffles and cuteSV. While such tools could in principle detect larger copy-number events affecting targeted genes, the highly variable depth of coverage inherent to targeted enrichment is likely to violate the assumptions of read-depth–based CNV detection methods, limiting their usefulness in this context.

Actions: limitations in detection of SVs is noted in the **DISCUSSION** and **METHODS** sections.

2.5. SV/CNV variant prioritization
The methods paragraph describing “variant prioritization” needs additional methodological detail and a clearer scope. It is currently ambiguous whether “variants” refers to SNPs/indels only or includes SVs/CNVs as well, and if both are included, which criteria apply to which variant class. We reviewed the PipeFace pipeline but could not locate documentation describing the allele-frequency annotation procedure, particularly for SVs.
In addition, the process used to add allele frequency (AF) annotations should be described explicitly, including: how AF was computed, whether AF was computed separately for SNPs/indels and SVs. Did you consider additional, larger allele frequency annotation sources for SVs, such as TopMed and CCDG, or tools like AnnotSV or SVAFotate that combine AF annotations from different sources? Finally, the statement “SVs intersecting protein-coding genes were prioritised” is underspecified. Is the predicted gene impact used in SV filtering/prioritization beyond gene intersection? For example, do you explicitly filter or rank SVs by categories such as predicted pLoF, copy gain, intragenic exonic duplications, or SVs affecting promoters/enhancers/UTRs? If so, please describe the annotation tools/criteria and how these impact categories are applied in filtering.

Variant prioritisation strategy
We refer the reviewers to response **2.3** above, which clarifies the use of *Pipeface* for variant calling and functional annotation and *PuzzleApp* for variant prioritisation. We have attempted to explain our variant priorisation strategy in more detail within the **METHODS** section, but also emphasise that every case is different, with filtering parameters and other criteria being flexibly adjusted as appropriate for a given case. Please let us know if there is still insufficient detail to understand the prioritization process.

Allele frequency annotation
As the reviewers likely appreciate, reliable allele-frequency annotation for SVs remains challenging due to heterogeneity in SV calling methodologies, breakpoint variability, reference representations, and differences in how population SV datasets are generated and normalised. As a result, there is currently no universally accepted approach for assigning population allele frequencies to SVs.
At the time this study was conducted, we explored several approaches and implemented SV annotation using Ensembl VEP against available ClinVar and gnomAD SV databases. These steps are executed within pipeface. In parallel, we performed cohort-level SV merging with Jasmine and comparison against available individuals from the 1KGP-ONT project, to deprioritise common and/or likely artefactual events not reliably captured by short-read datasets. This deprioritisation of common events is performed within PuzzleApp. We evaluated AnnotSV during development; however, we found that the resulting annotations were difficult to interpret at scale. We did not evaluate SVAFotate in this study.

SV functional annotation

SV functional impact annotation / prioritisation was based primarily on gene intersection and the consequence annotations provided by Ensembl VEP. SVs intersecting protein-coding genes were annotated with predicted transcript-level consequences (e.g. exon overlap, intronic, UTR, upstream/downstream effects), and these annotations were used to guide downstream filtering and review of candidate variants within PuzzleApp.

Actions: Additional detail on SV annotation process added to the **METHODS** section; usage of PuzzleApp and further description of variant prioritization strategy described in the **METHODS**; PuzzleApp to be public at time of publication.

2.6. Methylation analysis

The integration of methylation with allele size is valuable, especially for interpreting asymptomatic carriers, as seen in your case vignettes. Outside of d4z4ling, is methylation analysis automated or manually reviewed for specific loci? Could you please clarify the workflow.

Our 'Pipeface' pipeline produces phased 5mC methylation frequencies across target regions in BED format and .wig files for browser visualisation. Specific gene promoter regions with known epigenetic signatures of disease (such as the OPDM genes) are then assessed separately, using a custom script (included in our available code) and manually visualised in the browser where relevant. We hope to add a more generic, global analysis of epigenetic signatures to Pipeface in the future. This information has been added to the **METHODS** section.

Actions: further description of epigenetic analysis of gene promoter regions added to **METHODS**.

2.7. D4Z4 read-level methylation analysis

In Figure 2b, read-level methylation data appears to be averaged across the entire read. This likely dilutes the signal as reads could span various regions in addition to D4Z4. Could you please clarify if this is the case? If that is the case, we suggest an alternative approach. Your method already identifies the D4Z4 region in each read, so you could use that to identify the methylation level in that specific region. Consider refining the methylation analysis/visualization by reporting % methylation within the relevant target region(s) (e.g., the defined D4Z4 methylation region) rather than per read across the entire read.

The methylation values shown in **Fig. 2b** are not averaged across the entire read. Within *d4z4ling*, each read is first annotated with the full D4Z4 repeat structure and methylation is computed for all CpG sites along the read. However, for the analyses shown in **Fig. 2b** and related figures, we specifically report methylation levels restricted to the distal D4Z4 repeat unit only, which is the region most relevant to *DUX4* expression and FSHD pathophysiology. Therefore, the values labelled as "Distal D4Z4 5mC (%)" reflect methylation within this defined region. Values are averaged across all CpGs within the distal-most D4Z4 copy, but are not influenced by methylation in other portions of the read. We have clarified this in the legend for **Fig. 2**.

Actions: further detail on methylation values added to legend of **Fig. 2**.

2.8. d4z4ling GitHub reference

The d4z4ling GitHub repository references a separate preprint (https://www.medrxiv.org/content/10.1101/2025.04.24.25326320v1). Could you please clarify the relationship between the current manuscript and this publication? Did the data inform the development of the current method?

The *d4z4ling* software was developed independently of the preprint referenced in the GitHub repository. After our initial development was complete, the *D4Z4End2End* preprint became available and reported an alternative 4qB marker sequence. We noted that using this marker sequence improved specificity, compared to the sequence we were using at the time. We therefore adopted this marker sequence into *d4z4ling.* No data, analytical results or component of the algorithm from *D4Z4End2End* were used to develop our method. Because we adopted their marker sequence, we referenced the pre-print in the features table in the GitHub documentation. We have tried to clarify this in the GitHub repo where the

D4Z4End2End preprint is cited. We have also added a reference to our own current pre-print at the top of the *d4z4ling* repo and will update this should our manuscript progress to formal publication.

Actions: pre-print citations updated for clarity in the Github repo.

2.9. Citation(s) missing for genomic coordinates
"a custom panel of 331 loci associated with genetic myopathies (Table S1). These loci were chosen based on a literature review performed in January 2023, focusing on genetic myopathic disorders, supplemented by review of existing myopathy gene panels in PanelApp and from diagnostic genomic laboratories (Invitae, Blueprint Genetics and PathWest)."
The source(s) should be provided for the genomic coordinates in Supp Table 1, pathogenic thresholds for repeat loci, and any other similar information used as input into the panel creation. We suggest providing a citation/source column for Supp Table 1 if the sources were distinct papers/resources. Alternatively, if most regions were from the same or a small number of sources, you could simply cite them in the methods section with links if relevant.

We have added additional columns to **Supplementary Table 1** in which we provide OMIM Phenotype ID and an example PMID/reference linking each locus with a myopathy/muscle disorder. We have also created a new **Supplementary Table 4** in which we provide the individual external laboratory gene panel lists that were reviewed in the process of curating our own panel. Not all loci used by these external laboratories were incorporated into our panel for a number of reasons e.g. some loci were felt to be cause of neurogenic disorders rather than myopathic disorders, some loci were felt to produce conditions where myopathy was not the predominant phenotype (one of the inclusion criteria for our cohort), etc. We have updated the text in the **Methods** section to reflect this.

Actions: additional columns with reference sources added to **Supplementary Table 1** and new **Supplementary Table 4** created. Wording of **METHODS** section modified.

2.10. Genomic Coordinates
Please add genomic coordinates to all relevant figures or their legends, e.g., Figs 2a, 4c, 4d S8. If there are relevant genomic features within the figure, the exact positions of those would also be useful. For example, the specific coordinates of the FSHD locus features. Also, add the coordinates for all methylation regions identified by you or pulled from the literature, ideally in an easy-to-parse format, similar to Table S1. There is also a mixture of hg38 and T2T-chm13 coordinates used throughout the paper. So, double-check that the reference genome is clear for every coordinate, or choose a single coordinate system.

All variant detection and downstream analyses in this study are reported using hg38 coordinates. The T2T-chm13 reference is used internally within the d4z4ling pipeline to identify reads overlapping the D4Z4 locus and to differentiate between 4q and 10q, as the D4Z4 region is better assembled in the T2T reference genome. T2T-chm13 is also used as the reference sequence during ReadFish / adaptive sampling experiments, with enrichment targets specified relative to this assembly. To avoid confusion, we have added the reference genome build and relevant genomic coordinates to all applicable figures, including **Figs. 1d, 1e, 1f, 4c, 4d** and **Supplementary Figs. 3, 5a, 5b, 8, 9**. Note, **Figure 2a** is a stylised representation of the D4Z4 region, rather than a real browser view, so we have not added genomic coordinates to this panel.

Actions: genome build and genomic coordinates stated in all relevant figures.

2.11. Minor Comments
Confirm whether the pipeline was tested on WGS data. Is it specific to ONT (not PacBio)?
Two separate pipelines are used in this study: Pipeface for variant analysis and d4z4ling for FSHD-specific repeat and methylation analysis (see **2.3**). Pipeface has been tested on both whole-genome sequencing and adaptive sampling data, and is fully compatible with both Oxford Nanopore and PacBio long-read sequencing platforms. In contrast, d4z4ling was evaluated in this study using adaptive sampling (ReadFish) ONT data only. While there is no technical limitation in the pipeline that would prevent processing of whole-genome or PacBio data, the primary practical limitation for WGS is achieving sufficient coverage at the D4Z4 locus, which is more reliably obtained with targeted enrichment (either adaptive

sampling or cas9-based enrichment used elsewhere). In addition, the ~20kb read length of PacBio sequencing is likely to be insufficient for effective analysis of the D4Z4 region.
Actions: compatibility of pipeface and dz4zling pipelines with alternative data types clarified in the **DISCUSSION** and **METHODS** section.

2.12. Define "pseudodominance" (multiple definitions exist in the literature).

A definition for pseudodominance has been added to the text, as follows: "a phenomenon where an autosomal recessive disorder appears to follow an autosomal dominant pattern of inheritance".

Actions: 'Pseudodominance' defined where mentioned in the **RESULTS** section.

2.13. In Figure S1, the stacked bar plot for "age at onset" and "disease duration" implies that the sum equals current age, but this is not stated. Please clarify. If the intent is not to display the current age, consider an alternative visualization (e.g., separate bars or onset-to-current age segment plots).

Thank you for this feedback. There was no intent to conceal the current age. We have attempted to modify the plot, relevant labels and legend in order to clarify this point. Please let us know if it's still confusing.

Actions: Supplementary Figure 1A updated for clarity.

2.14. On page 6, the paragraph about cases L246 and L247. I found this challenging to conceptualize. Could you please provide a sentence or two overall summary and/or a supplementary figure showing why the previous testing was incorrect.

As requested, we have added the following additional description regarding L246 and L247: "The discrepancy in D4Z4 repeat number on SB between the siblings presumedly relates to the known error margin of SB. The misdiagnosis of L247 as having FSHD2 by BSS and the discrepancy between the BSS results of the siblings likely reflects the inability of BSS to assess allele-specific methylation levels in the presence of two 4qA alleles and/or that the diagnostic methylation thresholds of the previous BSS assay were not calibrated for the uncommon occurrence of biallelic FSHD1 D4Z4 contractions. Complex cases such as these highlight the utility of our assay, which is able to concurrently assess allele-specific D4Z4 contraction size, 4q haplotype and D4Z4 methylation, compared to typical SB or BSS assays that provide only partial genetic or epigenetic characterisation of the D4Z4 locus."

Actions: Additional sentences added to **RESULTS** section to clarify findings for L246 and L247.

2.15. Figure 2 has a lot of colours, resulting in a need to check the legend multiple times when interpreting it. You could simplify by using labelled boxes rather than background colour for the sample categories in panels c and e.

We thank the reviewer for this suggestion. We intentionally used a consistent colour scheme across all figures in the manuscript to facilitate interpretation of sample categories throughout the paper. While using labelled boxes instead of background colours in **Figure 2** panels **c** and **e** could be an alternative, there is insufficient space to include readable labels in these panels without substantially cluttering the figure. In our view, background colour therefore provides the clearest and most practical way to indicate sample categories in these panels, while maintaining consistency with the rest of the figures.

Actions: None.

2.16. Is 50% methylated significant? Why was this value chosen to highlight in Figures 2b and similar? Can you provide some general guidance on how to read these plots? If most points fall in a specific quadrant, is that important?

The 50% methylation dashed line is not intended as a statistical threshold, but rather as a visual reference point to aid interpretation of the plots, and we do not assign any statistical significance to this value. We empirically estimated methylation ranges using FSHD1 positive controls and non-FSHD samples and observed non-overlapping ranges (defined as median ± 2 × MAD) of 12.42–45.58% for contracted

pathogenic alleles in FSHD1 positive controls and 52.84–83.16% for non-pathogenic alleles in non-FSHD samples. Based on this, pathogenic alleles are expected to have most reads below 50% methylation (first quadrant), whereas non-pathogenic alleles tend to have most reads above 50% (second quadrant). We display all empirical thresholds as coloured lines in **Fig. 2e** and describe their meaning there, but felt that doing so for every figure would substantially clutter the plots. We therefore opted to include a single intuitive reference at 50%, while explicitly describing the statistically derived thresholds and their interpretation in the figure legends and text (e.g. **Fig. 2e**).

Actions: None.

2.17. On page 6, final paragraph, are you implying that the D4Z4 contraction on 10A176T observed in L117 is possibly pathogenic but with a different phenotype? Could you please clarify/elaborate?

Contractions on a 10A176T haplotype are not considered pathogenic for FSHD. To the contrary, our results show the presence of a D4Z4 contraction on this 10A176T allele appears to have resulted in a false-positive SB finding in case L117 and is not pathogenic. Reassessment of the phenotype also identified that there were features atypical for FSHD, such that the diagnosis of FSHD could be revoked. An alternative cause for this patient's clinical features was not identified. This paragraph has been rewritten to make this clearer.

Actions: Relevant paragraph revised for improved clarity.

2.18. Page 6, could you please define "permissive"? It is somewhat implied by context, but a more explicit definition would be helpful.

As requested, the relevant intro paragraph has been rewritten to more clearly reference/introduce the concept of permissive and non-permissive D4Z4 haplotypes in FSHD: "Genetic diagnosis of FSHD may involve a combination of SB to identify chromosome 4 D4Z4 macrosatellite repeat contractions and detect the FSHD-permissive 4qA haplotype (cf. non-permissive 4qB, 10qA, 10qB haplotypes) haplotyping (A or B) that contribute to FSHD type 1 (FSHD1), NGS to identify SNVs or indels in genes that cause FSHD type 2 (FSHD2), and/or bisulphite sequencing (BSS) to detect D4Z4 hypomethylation, seen in both FSHD1 and FSHD2."

Actions: Introductory paragraph re-written to better introduce concept of permissive D4Z4 haplotype.

2.19. What proportion of the curated panel regions achieved sufficient sequencing depth across samples? The current analysis (Fig S2b) considers depth at the per-sample level, not per region. Were certain regions consistently undersampled? It is useful to know if specific regions are undersampled; the overall sequencing may have to be increased to ensure all relevant regions are sequenced to the required depth.

We assessed coverage at the per-region level across all samples and found that, on average, 96.8% of samples per targeted region achieved ≥30x coverage, indicating that the majority of panel regions are consistently well covered. To assess whether specific regions were systematically undersampled, we compared coverage distributions across panel regions by ranking targets by median depth and plotting the bottom 40 and top 40 regions (**Supplementary Fig. 2**). Median coverage for the bottom 40 regions was 72.4x, compared to 94.7x for the top 40, indicating relatively modest variation across targets and no distinct subset of consistently poorly covered regions. The ABCD3 target region is a notable outlier in this analysis because it was added to the panel during the course of the study, and therefore approximately half of the samples did not include this target. Together, these data suggest that insufficient coverage in target regions is unlikely to be a major contributor to missed variants in this cohort, and that increasing overall sequencing depth would be expected to yield only marginal improvements in coverage uniformity across the panel.

Actions: additional per-target coverage analysis added to **Supplementary Fig. 2**.

2.20. In Supplementary Table 1, PLIN4 is listed under "other genetic myopathies". It may fit more naturally grouped with the tandem repeat disorders, as it is a VNTR locus.

The *PLIN4* gene has been moved to be grouped with the STR loci and the group renamed to "Short and variable number tandem repeat loci". Several other myopathy genes have also been moved within **Supplementary Table 1** to more accurate categories.

Actions: *PLIN4* and several other loci moved within **Supplementary Table 1.**

Reviewer #4 (Remarks to the Author)

4.0. The study by Yeow and Reis et. al. explores the utility of using Oxford Nanopore long read sequencing in unsolved neuromuscular diseases (NMD). They developed an optimization protocol for DNA sample preparation and then targeted sequencing of a panel of NMD genes. In addition, specialized software called d4z4ling was developed to accurately determine number of D4Z4 repeats associated with FSHD. Lastly, methylation of these regions were also explored. They were able to achieve 29% increase in diagnosis rate among myopathy patients that were undiagnosed from clinical genetic testing. The majority of these discoveries were repeat expansions in OPMD genes and FSHD cases with D4Z4 repeat contraction and/or changes surrounding in methylation.
The manuscript is very well written and figures were put together very nicely. The github repo looks well organized, which is extremely rare for most academic papers!

We thank the co-reviewers for their constructive review of our manuscript. Responses to their specific comments are provided below.

4.1. Major comments
The methods proposed in this manuscript would receive wider adoption if it was applied on a control group of data of unaffected individuals. There should be a set of publicly available data this could be applied to show the size distribution of STR in genes, common deletions in introns known in the population and also D4Z4 repeat distribution in the population. If there is also trio data available to look at transmission of these events. This would add to the downsampling result that was presented. Or does this approach require high coverage sequencing only achieveable through a targeted panel?

We agree that reference data on a population of unaffected individuals is useful to interpret the potential pathogenicity of genomic variants seen in myopathy patients. Some suitable, publicly available long-read sequencing datasets are now available. At the reviewers' suggestion, we have taken advantage of these where possible:

FSHD
Reliable analysis of the D4Z4 locus requires high read-depth and read-length, beyond what is generally obtained using whole-genome long-read seq with either ONT or PacBio (see response **2.11** above). Hence, there is no suitable population reference data available for us to use in this context. However, all pathogenic haplotypes seen in our study have been previously reported. Population reference data would be more essential if our study identified any novel haplotypes, not previously linked to disease, but this was not the case.

STR expansions
To assist in the interpretation of pathogenic STR expansions, we analysed data for 500 individuals currently available from the 1KGP-LRSC project (https://millerlaboratory.com/1KGP-LRS.html). This is helpful to define the scope of 'normal' allelic variation for STR sites where we have detected potentially pathogenic expansions in our cohort.

For example, the new figure below shows STR sizes detected among our cohort, compared to the 1KGP-LRSC population for OPDM genes *NOTCH2NLC*, *GIPC1*, *RILPL1* and *ABCD3*. Pathogenic ranges for these genes, taken from STRchive (https://strchive.org/), are shaded in pink. This analysis identifies all patients found to have OPDM (and their family members) as clear outliers from the 'normal' population. Interestingly, the 1KGP-LRSC population contains one individual (HG00653) with a very large *GIPC1* expansion (549 copies), exceeding any individuals in our cohort. Notably, this extreme expansion exhibits allele-specific hypermethylation, which has been reported to silence expression and prevent disease (see IGV screenshot below).

Overall, the 1KGP-LRSC cohort provided useful population reference data which further strengthened the evidence for pathogenicity of STR expansions causing OPDM in patients in our cohort. These analyses have been included in **Supplementary Figure 8** and referred to at several places in the main text.

b. **STR sizes comparison between the Myopathy cohort and the 1KG-LRSC population for OPDM genes.**

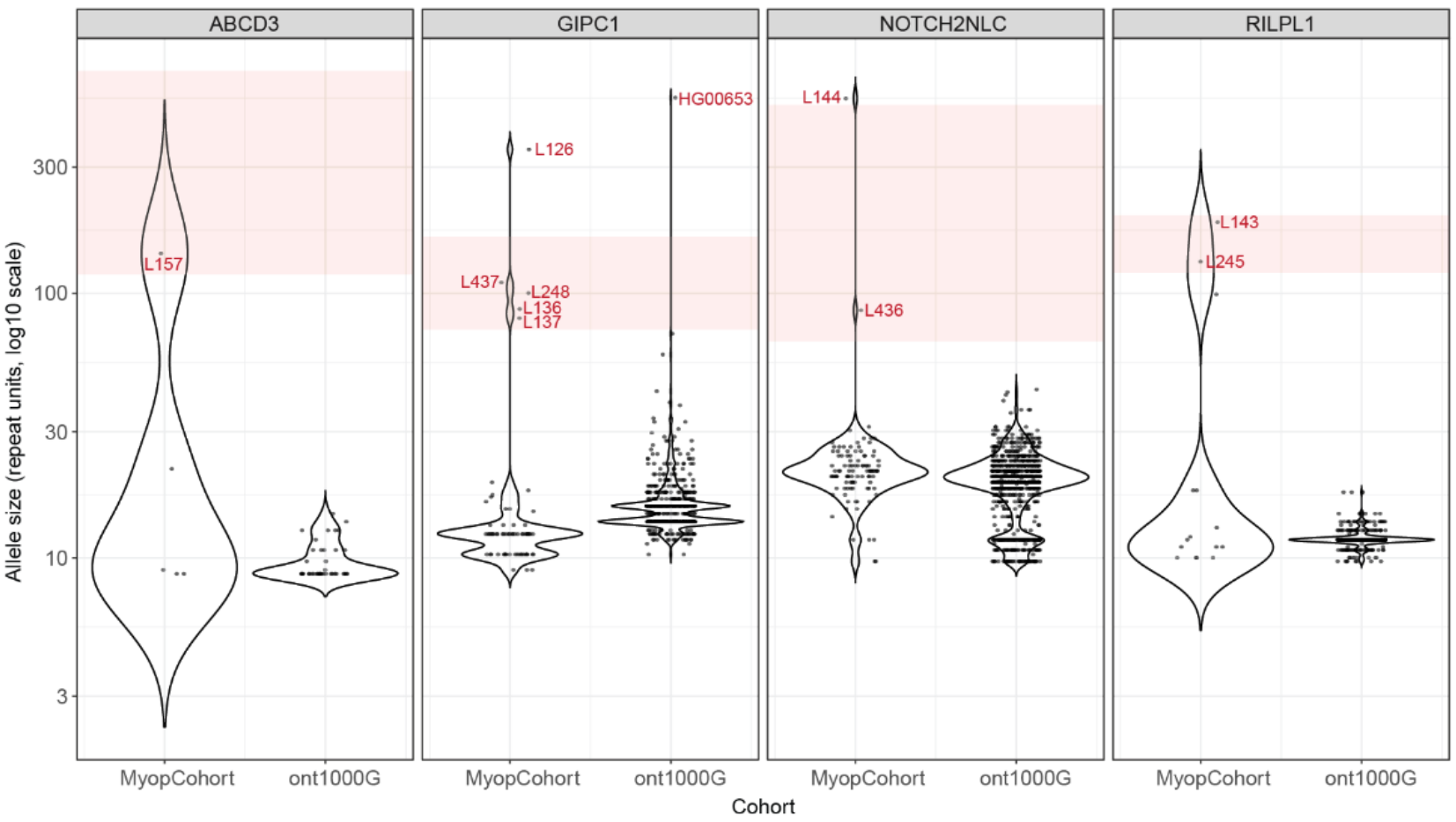

c. **HG00653 GIPC1 expansion and read-level methylation.**

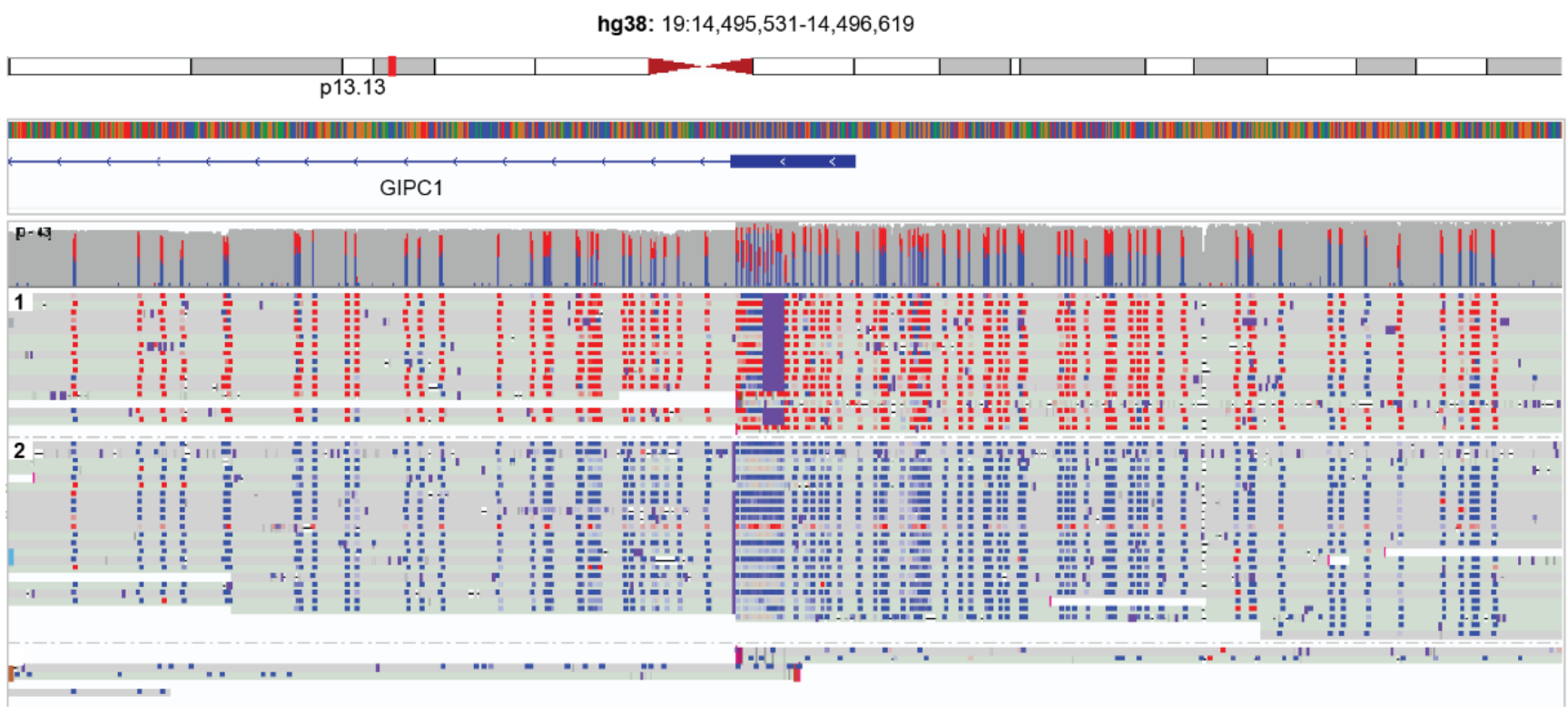

VNTR expansions

Similar to STRs, we have also now used the 1KGP-LRSC population data to interpret the pathogenicity of the *PLIN4* VNTR expansion detected in one individual (see response **1.2**, above)

Structural variants

We also utilised the 1KGP-LRSC data to help assess the allele frequency of SV candidates detected among our cohort. All candidates were filtered against the 1KGP-LRSC population using all available data to de-prioritise likely common SVs, as well as recurrent sequencing/alignment errors. In this context, we are not specifically concerned with the size or type of a given SV, but rather whether it is seen in multiple unaffected individuals, and is hence likely benign. We have added additional details to the **METHODS** section to clarify the essential role population reference data played in interpreting/prioritising SV candidates.

Transmission of variants

Almost all of the cases in our study were singletons, with family members either not available or not recruited. Therefore a general analysis of transmission for SVs, STRs, etc would not be meaningful. However, we have included information on the transmission of STR expansions in *NOTCH2NLC*, *GIPC1* and *RILPL1*, among OPMD cases where family members were available (see OPDM section and **Figure 4)**.

Actions: Analysis of population reference data from 1KGP-LRSC added to **Supplementary Fig. 8** (OPDM STRs) and **RESULTS** section (OPDM STRs; PLIN4 VNTR; etc); use of population reference data during SV prioritisation described in the **METHODS** section; examples of STR transmission within OPDM families described in **RESULTS** and **Fig 4**.

4.2. There are a few very feasible missed opportunities that this study did not explore with the data generated:
a. Sequencing data offers more information than just repeat size, which is really the only thing that southern blotting and PCR methods can provide. It is hypothesized by others that sequence diversity/heterogenity in the repeat units may contribute to pathogenicity.
b. Any interesting methylation pattern changes in genes specially in unsolved cases. It may be implied that there were none to comment but at least explicitly mention this.
c. Large structural variants detected. Its assumed that no overlapping coding regions were detected as they would have been explicitly mentioned. However, it would be good to mention any non-coding structural variants detected and whether they were common in the population. This highlights the utility of this approach to detect SV even though for this cohort none were considered causal.

a. Though not mentioned in our original manuscript, our STR analysis can identify the presence of non-canonical STR motifs and/or interruptions, which may be relevant to the pathogenicity of a given repeat. However, in this case all STR expansions (seen in *NOTCH2NLC*, *GIPC1*, *RILPL1*, *ABCD3*) were canonical 'GGC' repeats and no non-canonical motifs or interruptions were observed. Therefore, our analysis focused on STR sizes and methylation patterns. To avoid confusion, we have now explicitly stated this in the relevant **RESULTS** section and legend of **Figure 4**.

b. Our DNA methylation analysis involved an unbiased 'outlier detection' analysis, in which we considered the methylation state of all promoter regions within our gene panel, across the entire cohort (visualised in the heatmap below). This analysis identified the differential methylation of the *GIPC1* and *NOTCH2NLC* promoters seen in specific individuals, relevant to OPMD, as summarised in **Figure 4**. The analysis also identified a small number of promoter-methylation outliers in other genes, such as hypermethylation of the *SGCD* promoter in one individual. Although these events are interesting, we are unable to make any inferences about their pathogenicity, as they are seen in only single individuals and have no previous known epigenetic signatures associated with disease. At the reviewer's suggestion we have explicitly mentioned this in the **METHODS** section.

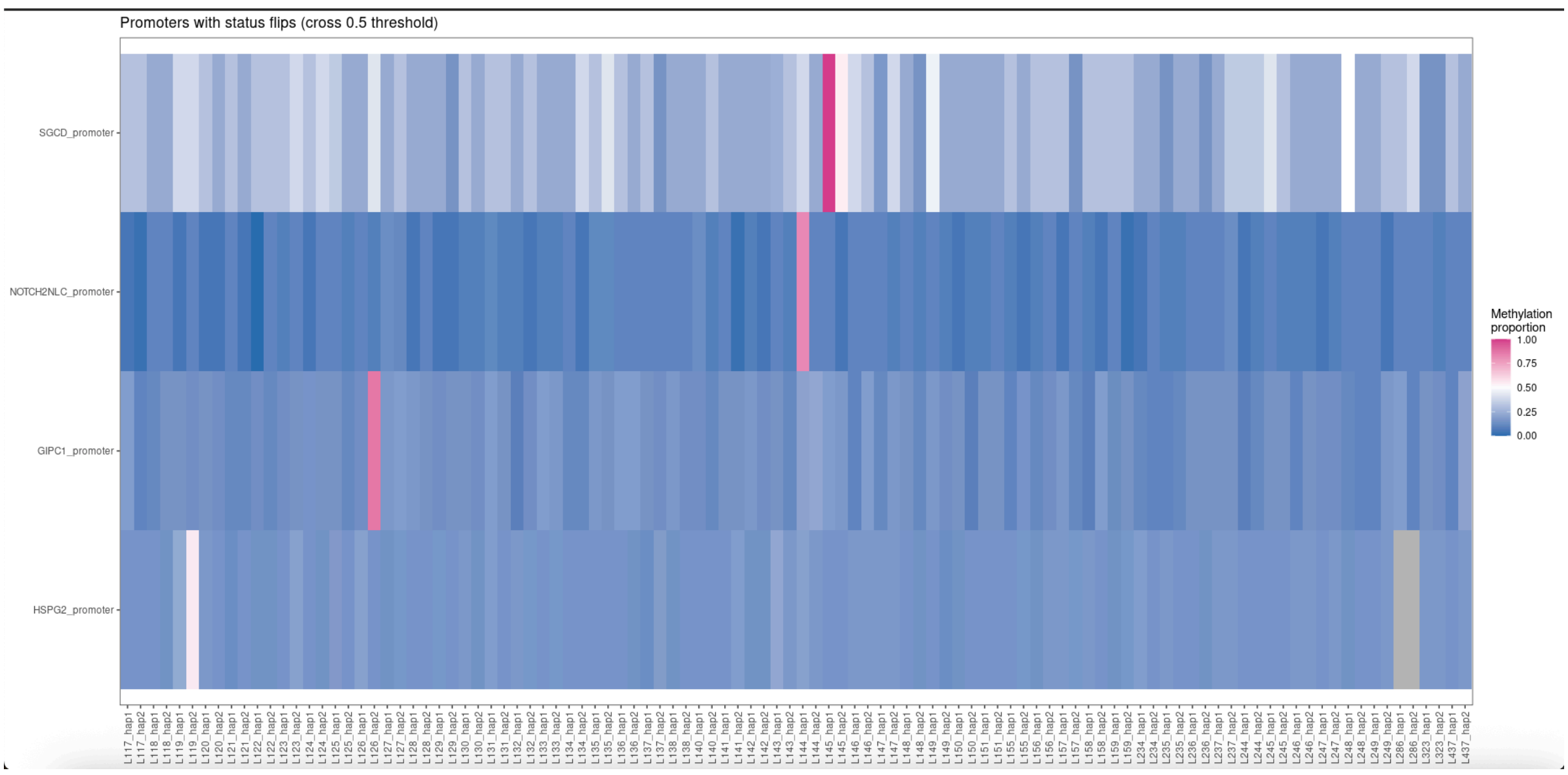

c. Because our sequencing assay is targeted to gene loci, we are less likely to detect intergenic non-coding SVs. However, we do detect some such events, in addition to SVs within introns and UTR regions of protein-coding genes. All of these are considered during SV prioritisation, but they were de-prioritised if they are seen commonly within the population (1KGP-LRSC population reference; see **4.1** above). We have included an additional supplementary table (**Supplementary Table 3**) summarising the detection and prioritisation of SVs, and the reviewer is correct that none were considered causal.

Actions: statement that non-canonical STR motifs and/or interruptions were not detected among STR expansions added to **RESULTS**; additional detail on methylation analysis and detection of novel differentially methylated genes in **METHODS** section; new **Supplementary Table 3** summarises detection of non-coding SVs detected per individual.

4.3. It is clearly demonstrated in the manuscript that ONT long read sequencing can accurately detect STRs in OPMD genes and also FSHD D4Z4 repeats. The (?)additional new diagnostic finding in LAMA2 should discuss why it was missed by clinical sequencing as this may be valuable for readers to learn.
a. The small intronic deletion in LAMA2 is close enough to an exon (Figure 5C) that it should have been detected by exome or panel sequencing. It would be good for the authors to show what the existing NGS data looks like for this sample.

We agree that small intronic deletion in *LAMA2* is close enough to the exon that it could foreseeably have been identified by the participant's previous NGS targeted gene panel. The reporting clinical laboratory was contacted on numerous occasions and did not respond to our requests for more information about why this variant was not reported and/or detected. We suspect low coverage in the exon edge region could have caused the variant to be missed or filtered out, or that the variant was detected but not reported as it would have remained a VUS without the splicing studies performed in our study. However, we can only speculate because we have not seen the original NGS data.

Actions: None.

4.4. The variant filtering thresholds used could be considered very permissive and would be good for the authors to comment/justify on the following:
a. gnomAD allele fequency threshold of 0.01
b. SpliceAI threshold of 0.2. The candidate splice variants in L323 within CAPN3 have quite low prediction values. Noted that the authors did mention it will require further validation.

The reviewer is correct that our initial filtering thresholds of 0.01 allele frequency and 0.2 SpliceAI score (PMID: 34267211) could be considered overly permissive. However, these are appropriate for our study design. Our analysis considers only a fairly small number of genes included in our target panel (n=331) and our cohort size is also relatively small. This means we can afford to use relatively permissive starting criteria to maximise sensitivity, followed by more careful review of all candidate variants, and secondary validation where this is called for (e.g. for the *LAMA2* variants). For genome-wide analysis or larger cohorts, this strategy may not be feasible, in which case we would adjust these and other criteria to increase filtering stringency. In our revised manuscript, we have elaborated on these filtering criteria and emphasised that these are flexible parameters that users should adjust for the specific nature of their assay, cohort and available resources.

The *CAPN3* c.2440-75C>G variant in L323 is an example of the utility of using a SpliceAI threshold of 0.2. The predicted SpliceAI scores for the variant were only Δ0.28 for donor gain and Δ0.33 for accept gain. However, our RT-PCR experiments here (**Fig. 5f** and **Supplementary Fig. 11A**) showed the variant does, in fact, disturb splicing. More strict SpliceAI thresholds during variant filtration may have resulted in this variant being filtered out.

Actions: sentences added to **METHODS** justifying variant filtering thresholds and encouraging users to adjust for their own context.

4.5. Distinguishing between 4qA, 4qB and 10qA haplotypes is important for determining pathogenicity of D4Z4 contractions. However, there is little explicit information on how this classification is achieved in the context of LRS through using d4z4ling. Mentioning restriction sites in Figure 3I lacks details. Shouldn't the tool be looking for a set of unique sequence or patten of sequences rather than just a short restriction enzyme site/sequence occurring by chance all over the genome?

We agree that 4qA, 4qB and 10qA haplotype assignment cannot rely on the presence of a single short motif in isolation. We have added additional detail to the **METHODS** section explaining how the assignments are made. To summarise:

Our *d4z4ling* software first considers the alignment of each sequencing read to the T2T-chm13 reference, which typically provides unambiguous mapping to either chromosome 4 or chromosome 10 D4Z4 loci. This establishes whether the read is '4q' or '10q' and constrains the haplotype search space.
Each read is then annotated for a set of haplotype-specific sequence features, including: (i) p13-E11 (5′) and pLAM (3′) flanking sequences; (ii) SSLP length polymorphisms; (iii) sequence of the distal-most D4Z4 unit; (iv) presence of 4qA/4qB-specific probe sequences; (v) diagnostic restriction enzyme recognition sites (BlnI and XapI); and (vi) proximal SNVs and indels. Haplotype assignments are made using concordant evidence across multiple independent features on the same read or phased haplotype, rather than relying on any single motif. The BlnI and XapI restriction sites are used as supporting evidence within this multi-feature framework, primarily as a consistency check to flag reads that may have been misclassified between the 4q and 10q loci, minimising the risk of misclassifying reads.

Figure 3I illustrates the importance of using multiple concordant features rather than relying on restriction motifs alone. In case L117, we observed chimeric reads with alignments spanning both the chromosome 4 and chromosome 10 D4Z4 loci. These reads can appear "4q-like" based on restriction enzyme sites that are consistent with 4q sequence, and in isolation could be misinterpreted as supporting a 4qA allele. However, they lack the expected 4qA distal features, in particular the pLAM polyadenylation signal (PAS). By anchoring interpretation to the T2T-chm13 alignment context and requiring concordant evidence across distal markers (including PAS) and other haplotype features, flags these reads for inconsistency, supporting correct classification in the presence of 4-like restriction patterns.

Actions: Further information on the process of haplotype assignment for individual reads within d4z4ling has been added to the **METHODS** section.

4.6. Case L121 was presented to not have FSHD1 due to having 11 copies of D4Z4 instead of the magical threshold of 10 copies. How well established is the 10 copy pathogenicity threshold and the underlying biological mechanism that disease is caused when this threshold is crossed? Note this is different to the point made in the manuscript, which related to incorrectly counting 11 repeats (via SB) when the true number is 10 repeats. Putting this in perespective of the patient to miss out on exciting therapies in clinical trials because no one dared to challenge the 10 copy threshold and the underlying evidence!
a. Is Figure 3L mislabelled/typo as it references L147, should it be L121 instead?

The 10-copy D4Z4 pathogenicity threshold is widely accepted and endorsed by international guidelines for FSHD diagnosis (PMID: 38685133). However, D4Z4 size assessment and therefore diagnostic threshold setting has historically relied on assessment by SB, which is associated with an error margin that increases with longer fragment lengths (e.g. ~10 repeats or above). If 11 unit D4Z4 arrays could, in some rare cases, cause FSHD, it is possible these cases could have been previously mis-assessed as being 10-copy D4Z4 units due to the error margin of SB. Furthermore, it has been more recently appreciated that a hard cut-off of ≤ 10 copies for pathogenicity is not completely accurate; the penetrance of 8-10 copy contractions on 4qA haplotypes is now known to be incomplete, such that some carriers of these contractions have FSHD while others remain healthy and do not develop FSHD. Therefore, it is possible that 11-copy D4Z4 unit contractions could potentially also demonstrate incomplete penetrance, perhaps with the majority of 11 D4Z4 unit 4qA carriers remaining healthy (explaining the large amount of historical data suggesting that 11 repeat contractions do not cause FSHD) and only occasional individuals (possibly such as L121 here) developing FSHD.

In order to further investigate this, we have now sequenced the affected brother of L121 and identified he also harbours a 11 unit D4Z4 array on a 4qA allele. However, the link between 11 unit D4Z4 arrays and FSHD1 remains very speculative. Our report of two siblings with an 11-copy D4Z4 contraction on a permissive 4qA allele and clinical examinations consistent with a FSHD-pattern of muscle involvement is insufficient, by itself, to challenge the current consensus diagnostic threshold of ≤ 10. However, we agree with the reviewer that this result should be highlighted and explored in future work. We have therefore added additional material to the **DISCUSSION** outlining the implications and caveats around this result. The typographical error of L147 has been corrected to L121 in the Fig 3 label.

Actions: new sentences in **RESULTS** regarding finding of a 11-copy D4Z4 array in the brother of L121, new sentences in **DISCUSSION** on potential for pathogenicity of 11-copy D4Z4 haplotypes, context and caveats to this result; L147 to L121 typo corrected in **Fig3L** label/caption.

4.7. Minor comments
Can the d4z4ling be used on PacBio long read sequencing or easily adapted? This could increase it's more wide usage.

Please see response **2.11** above.

Actions: compatibility of pipeface and dz4zling pipelines with alternative data types clarified in the **METHODS** section.

4.8. The concept of on target and off target presented in Figure S2a may need to be clearly explained to readers. How can off targets occur in the context of using ONT and Readfish as many readers may be unfamiliar.

The ReadFish / Adaptive sampling method is not 100% efficient, so some off-target read always make it through. In addition, those reads that are rejected during sequencing typically still show up in the analysis, because the first ~100-500bp has been sequenced prior to a rejection signal being sent. Many of these short, rejected reads will appear in off-target regions. We have clarified this in the legend for **Supplementary Figure 2**.

Actions: legend for **Supplementary Figure 2** updated to give more context on off-target reads.

4.9. Coverage presented in Figure S2b is quite variable. Are the samples with coverage below 100x correlated to any DNA QC metric? Run time or adjusted software settings?

While it is true that DNA quality may have an important impact on ONT sequencing yields, especially when using adaptive sampling, this was not a big factor in our study because all samples were extracted from blood samples with minimal delays between collection and extraction, using a well-practiced protocol for HMW DNA extraction. Therefore, the DNA we were working with was of generally very high quality.

The biggest driver of the variable coverage seen in **Supplementary Figure 2** was how many samples were multiplexed on a given flow cell. Multiplexing was not standardised across the study, because sequencing was performed on patient samples as they were recruited and collected, meaning some were sequenced alone whereas others were batched up to 4 samples per flow cell. We have clarified this is the **METHODS** section and figure legend.

While there is variability in observed coverage, all samples were sequenced to an excess of coverage, meaning this variability should have negligible impact on the downstream analysis.

Actions: detail on variable multiplexing clarified in the **METHODS** section and legend of **Supplementary Fig 2**.

4.10. The authors should consider discussing and giving their thoughts/opinion on the value of targeted long read sequencing compared to short read WGS and 30x (PacBio) long read sequencing to increase the diagnostic yield on exome/panel negative samples. For example can the things detected in this study

be detected using short read WGS or on a 30x PacBio sequenced genome? Or are the higher targeted coverages necessary? In addition, there is mention of cost effectiveness of this approach but no comparison with short read WGS, is it about the same price or cheaper? Most people would not know tis.

There's a bit to unpack here. Firstly, short-read WGS is not suitable for FSHD analysis or properly resolving STR expansions, which are central to diagnosis of myopathies. While clinical exome sequencing is useful for myopathy diagnosis, we don't see much value in proceeding to short-read WGS for undiagnosed cases. Long-read WGS with either PacBio or ONT could deliver most of the diagnoses we have achieved using our targeted approach. The exception is for FSHD analysis, where higher read depth and length are necessary, making target-enriched ONT sequencing preferable (see **2.11**). The other drawback for long-read WGS is cost: targeted sequencing reduces the cost be ~2-3X per sample, on average. However, our targeted approach suffers from one key limitation: it is not suitable for discovering new disease genes/variants outside of the target panel. Given this, long-read WGS may be preferable for certain contexts and cohorts, and indeed many of our patients who remain undiagnosed after targeted ONT sequencing will be followed up with long-read WGS with a view to gene discovery.

Actions: additional commentary on pros and cons of different sequencing methods for different contexts has been added to the **DISCUSSION.**

4.11. The authors should consider discussing using hypomethylation in combination with any variants detected in known FSHD2 associated genes to resolve VUS.

We agree that our assay, which can concurrently assess the genetic and epigenetic structure of the D4Z4 locus, is well suited to helping resolve potential VUS in FSHD2-related genes in a single assay. As suggested, we have added the following sentence: "The ability to concurrently sequence FSHD2-related genes and characterise the genetic and epigenetic structure of the D4Z4 locus uniquely allows our single assay to detect and then, by identifying a pattern of D4Z4 hypomethylation consistent with FSHD2, help resolve VUS in FSHD2-related genes".

Actions: New sentence on resolving VUS in FSHD genes added to the **DISCUSSION.**

4.12. The authors should consider mentioning some of the challenges there may be to interpret variants/changes detected by this approach where there is no good population reference to compare against. This would be a challenge within known and novel disease genes.

We agree that the relative lack of population reference data generated with long-read sequencing (ONT or PacBio) is an important limitation, which hinders the interpretation of previously unreported SVs, STR expansions, etc. Fortunately, there has been some recent progress in this area, with the availability of raw ONT data for ~500 healthy individuals via the 1KGP-LRSC resource (see **4.1**, above), plus processed Pacbio HiFi data on >1000 individuals via the colorsDB resource (https://colorsdb.org/), among other sources. As suggested, we have added some commentary on this issue to our **DISCUSSION**.

Actions: sentences added to the **DISCUSSION** noting challenges caused by lack of long-read reference data, and highlighting some recent progress in this area.

4.13. Figure panels labels are in lower case letters, while the figure caption text uses capitalized letters.

We have updated the figure references in the main text so that the panel labels match the figures and are consistently in lower case, as per *Nature Comms* style.

Actions: Figure references in text updated to match figures.

Reviewer #4 (Remarks on code availability):

4.14. As noted in the intro summary there is a great github repo for d4z4ling.

Thank you for the positive feedback on the d4z4ling github.

Actions: None required.

4.15. Looking at the README it is extremely confusing without context regarding being a NCI user and the IF89. It makes absolutely no sense at the moment. It may be irrelevant to running it but authors should clarify.

We apologise for the confusion. NCI refers to Australia's National Computational Infrastructure, a supercomputing facility used by researchers across Australia. We recognise that this context is not relevant or clear for users outside Australia. To address this, we have removed this from the main README and created a separate, dedicated README describing the NCI-specific setup and usage as a specialised use case.

Actions: separate README created for specific usage on Australia's NCI supercomputer.

4.16. There are still hard coded lines in the code that hasn't been corrected. For example: https://github.com/neysa-15/d4z4ling/blob/main/fshd1_annotate_fasta_script.sh#L155

We thank the reviewer for flagging this. We have corrected such issues in the code and updated the github repository.

Actions: hard-coded scripts in github repo have been corrected.

4.17. Although the code for d4z4ling is available, there is no reference to additional repository containing the code used to generate figures and analyses presented in the manuscript. Noted that the underlying patient sequencing data can't be shared but the code used to process the data should be along with any processed data that can be shared.

We have now created an additional public GitHub repository (https://github.com/neysa-15/myopathy_lrs_plots) containing the scripts and workflows used to generate the analyses and figures presented in the manuscript. The repository is referenced in our revised **CODE AVAILABILITY** section: https://doi.org/10.5281/zenodo.19601174.

We also refer the reviewer to response **2.1** above, regarding availability of patient sequencing data, which is now hosted on dbGAP.

Actions: Github repo and Zenodo reference created for code used during manuscript production.
